# Cold-induced expression of a truncated adenylyl cyclase 3 acts as rheostat to brown fat function

**DOI:** 10.1038/s42255-024-01033-8

**Published:** 2024-04-29

**Authors:** Sajjad Khani, Hande Topel, Ronja Kardinal, Ana Rita Tavanez, Ajeetha Josephrajan, Bjørk Ditlev Marcher Larsen, Michael James Gaudry, Philipp Leyendecker, Nadia Meincke Egedal, Aylin Seren Güller, Natasa Stanic, Phillip M. M. Ruppert, Isabella Gaziano, Nils Rouven Hansmeier, Elena Schmidt, Paul Klemm, Lara-Marie Vagliano, Rainer Stahl, Fraser Duthie, Jens-Henning Krause, Ana Bici, Christoph Andreas Engelhard, Sabrina Gohlke, Peter Frommolt, Thorsten Gnad, Alvaro Rada-Iglesias, Marta Pradas-Juni, Tim Julius Schulz, Frank Thomas Wunderlich, Alexander Pfeifer, Alexander Bartelt, Martin Jastroch, Dagmar Wachten, Jan-Wilhelm Kornfeld

**Affiliations:** 1 Institute for Genetics, University of Cologne, Cologne, Germany; 2 Max Planck Institute for Metabolism Research, Cologne, Germany; 3 Department for Biochemistry and Molecular Biology, University of Southern Denmark, Odense, Denmark; 4 Novo Nordisk Foundation Center for Adipocyte Signaling (Adiposign), University of Southern Denmark, Odense, Denmark; 5 Institute of Innate Immunity, Medical Faculty, University of Bonn, Bonn, Germany; 6 Department of Molecular Biosciences, The Wenner-Gren Institute, Stockholm University, Stockholm, Sweden; 7 Institute for Cardiovascular Prevention (IPEK), Ludwig-Maximilians-University, Munich, Germany; 8 Centre for Physical Activity Research, Department of Infectious Diseases, Rigshospitalet, Faculty of Health Sciences, University of Copenhagen, Copenhagen, Denmark; 9 Department of Adipocyte Development and Nutrition, German Institute of Human Nutrition Potsdam-Rehbrücke, Nuthetal, Germany; 10 Institute of Human Genetics, University Medical Center Hamburg-Eppendorf, Hamburg, Germany; 11 Institute of Pharmacology and Toxicology, University Hospital, University of Bonn, Bonn, Germany; 12 Institute of Biomedicine and Biotechnology of Cantabria (IBBTEC), CSIC/University of Cantabria, Santander, Spain; 13 Novo Nordisk Foundation Center for Basic Metabolic Research (CBMR), Copenhagen, Denmark; 14 German Center for Diabetes Research (DZD), München-Neuherberg, Germany; 15 Institute for Diabetes and Cancer (IDC), Helmholtz Center Munich, German Research Center for Environmental Health, Neuherberg, Germany; 16 German Center for Cardiovascular Research (DZHK), Partner Site Munich Heart Alliance, Munich, Germany; 17 Department of Molecular Metabolism and Sabri Ülker Center for Metabolic Research, Harvard T.H. Chan School of Public Health, Boston, MA USA

**Keywords:** Metabolic diseases, Molecular medicine, Metabolism, Fat metabolism, Enzyme mechanisms

## Abstract

Promoting brown adipose tissue (BAT) activity innovatively targets obesity and metabolic disease. While thermogenic activation of BAT is well understood, the rheostatic regulation of BAT to avoid excessive energy dissipation remains ill-defined. Here, we demonstrate that adenylyl cyclase 3 (AC3) is key for BAT function. We identified a cold-inducible promoter that generates a 5′ truncated AC3 mRNA isoform (*Adcy3-at*), whose expression is driven by a cold-induced, truncated isoform of PPARGC1A (PPARGC1A-AT). Male mice lacking *Adcy3-at* display increased energy expenditure and are resistant to obesity and ensuing metabolic imbalances. Mouse and human AC3-AT are retained in the endoplasmic reticulum, unable to translocate to the plasma membrane and lack enzymatic activity. AC3-AT interacts with AC3 and sequesters it in the endoplasmic reticulum, reducing the pool of adenylyl cyclases available for G-protein-mediated cAMP synthesis. Thus, AC3-AT acts as a cold-induced rheostat in BAT, limiting adverse consequences of cAMP activity during chronic BAT activation.

## Abstract (web summary)

Truncated version of adenylyl cyclase 3 acts as a cold-induced rheostat during long-term brown adipose tissue activation.

## Main

Most mammals harbour two morphologically and functionally distinct types of adipocytes (fat cells): White adipocytes (WAs) consist of a single lipid droplet^1^ and predominantly ensure energy storage, whereas brown adipocytes (BAs) are multilocular and possess the ability to convert (diet-derived) macronutrients, like carbohydrates and lipids, into heat in a molecular process termed non-shivering thermogenesis (NST)^2^. Interest in BAT arose after the seminal discovery that (i) BAT exists in small animals like rodents and in human infants, and that adult humans can possess active brown fat^3–5^ and that (ii) cold ambient temperature exposure or stimulation of beta-adrenergic G-protein-coupled receptors (GPCRs) activates BAT, which positively correlates with energy expenditure^6^ and inversely correlates with body mass indices^7,8^.

GPCRs regulate a plethora of biological processes and are pharmacological targets for many drugs in clinical use^9^. GPCRs play a central role in adipose tissue homeostasis and, conversely, adipocyte metabolism is regulated by GPCRs that are linked to different functional classes of heterotrimeric G proteins^10^. The main physiological stimulus for NST in brown and thermogenically activated brown-in-white (‘brite’) fat is cold temperature, which activates the sympathetic nervous system and releases the neurotransmitter nordrenaline^11^. Noradrenaline (NE) activates beta-adrenergic GPCRs^12^, which then stimulate synthesis of the second messenger cAMP by transmembrane adenylyl cyclases (ACs)^13,14^. In turn, this activates protein kinase A (PKA), thus inducing lipolysis. The ensuing generation of free fatty acids stimulates uncoupling protein 1 (UCP1), a mitochondrial protein whose expression and activation drives NST in brown and brite fat^2^. In addition to these acute effects, chronic cAMP signalling is required for adipocyte precursor differentiation^2^. AC3 is expressed in different somatic tissues like adipose tissue, kidney, pancreas and liver as well as in olfactory sensory and hypothalamic neurons^15^. AC3 received attention due to loss-of-function variants in human *ADCY3* that correlate with enhanced susceptibility to increased body mass index^16^. Furthermore, *hADCY3* loss-of-function mutations cause severe obesity in human populations^17–19^. Mouse studies confirmed that *Adcy3* loss impairs energy homeostasis^20–24^, yet the precise role of AC3 in thermogenic fat has not been addressed yet.

Pre-mRNA splicing and alternative promoter commissioning are two fundamental, gene-regulatory mechanisms to increase proteome complexity from finite amounts of exonic information. These processes are important for fat function, cold tolerance and metabolic health^25–27^. Recent full-length RNA-sequencing (RNA-seq) studies suggest that other alternative promoter events contribute to metabolic regulation in thermogenic fat^28^. For instance, peroxisome proliferator activated receptor gamma (PPARG) coactivator 1 alpha (PPARGC1A), a key transcriptional co-regulator, can use alternative 5′ promoters, thereby generating C-terminally truncated proteins, which drive distinct transcriptional programmes in human muscle^29,30^. Here, we describe a new molecular rheostat, a truncated AC3 protein termed AC3-AT, that is selectively induced after cold and after beta-adrenergic stimulation and controls brown fat function. AC3-AT limits cAMP synthesis by controlling the plasma membrane (PM) localization of other AC isoforms and, thereby, curbing energy dissipation. Truncated AC3-AT protein isoforms are evolutionarily conserved, found in BAs from rodents to humans, and part of a conserved PPARGC1A–AC3-AT network that links thermogenic activation to adaptive protein isoform changes.

## Transcriptional regulation of cAMP biosynthesis during cold adaptation of BAT

**Fig. 1:**
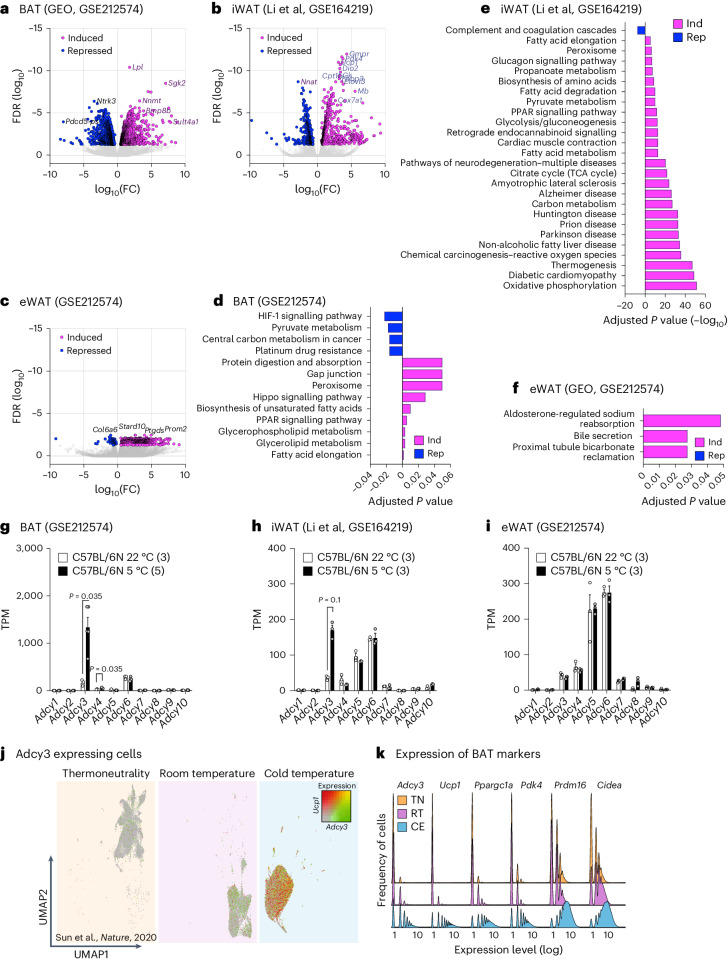
Transcriptional regulation of cAMP biosynthesis during cold adaptation of adipose tissue. **a**–**c**, Volcano plots of significantly upregulated (magenta) and downregulated (blue) genes in BAT (**a**; *n* = 2,290; 1,086/1,204 upregulated/downregulated differentially expressed genes (DEGs)), iWAT (**b**; *n* = 1,252; 804/448 upregulated/downregulated DEGs) and eWAT (**c**; *n* = 655 upregulated/downregulated DEGs) in 20-week-old C57BL/6N (**a** and **c**) or C57BL/6J (**b**) male mice at RT (22 °C) or after 24 h (**a** and **c**) and 72 h (**b**) of 5 °C CE, determined by mRNA-seq. Statistically significant DEGs were defined as false discovery rate (FDR) ≤ 0.05 and log transcripts per million (TPM) > 0. FC, fold change. *N* = 3–5 animals per temperature condition were analysed. **d**–**f**, KEGG pathway enrichments (Benjamini & Hochberg-corrected *P* value ≤ 0.05) for DEGs shown in **a**–**c**. **g**–**i**, mRNA expression of AC (*Adcy*) isoforms in BAT (**g**), iWAT (**h**) and eWAT (**i**), determined by mRNA-seq and depicted in TPM. *N* = 3–5 animals per temperature condition were analysed. Bar graphs represent the mean + s.e.m. with all data points plotted. Unpaired, two-tailed and non-parametric Mann–Whitney tests were performed to assess statistical significance. *P* values are indicated. **j**,**k**, Analysis of public snRNA-seq data (E-MTAB-8562) from Adipoq-tdTomato-positive adipocyte nuclei isolated from male mice housed at TN, RT and after CE at 8 °C for 4 days. The colours in **j** depict expression of *Adcy3* (green), *Ucp1* (red) or both (yellow). **k**, Ridgeline plots depicting frequency (in nuclei) of expression of BAT identity genes at indicated temperatures. Source data

**Extended Data Fig. 1:**
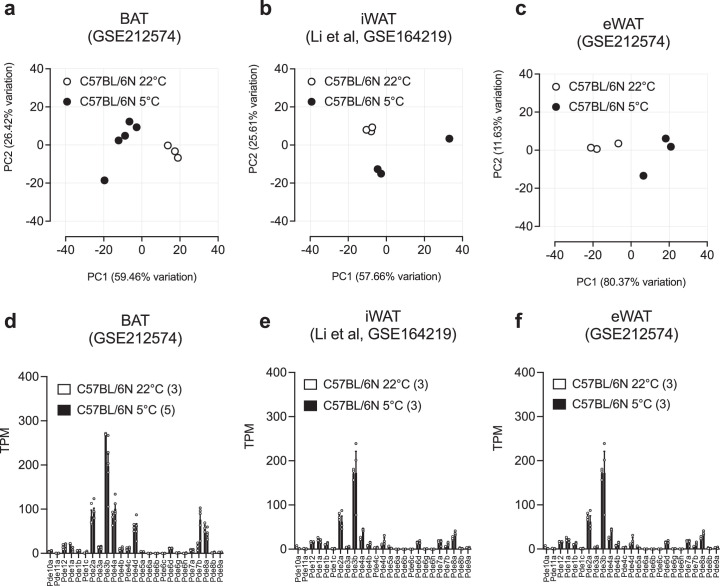
cAMP biosynthesis in adipose tissue after cold. Transcriptional regulation of cAMP biosynthesis in adipose tissue after cold exposure. **a**–**c**, Principal component analysis (PCA) of transcriptomic changes in BAT (**a**), iWAT (**b**), and eWAT (**c**) after 24 h (**a**,**c**) or 72 h (**b**) of 5 °C cold exposure (n = 3–5 per condition). Dots represent individual, chow diet fed, male C57BL/6N animals with n = 3–5 animals per condition. **d**–**f**, mRNA expression of phosphodiesterases (Pde) in BAT (**d**), iWAT (**e**) and eWAT (**f**) as determined by mRNA-seq and depicted in transcripts per million (TPM). Bar graphs represent mean + SEM with all data points plotted. Unpaired, two-tailed, and non-parametric Mann-Whitney tests were performed to assess statistical significance. Source data

To identify transcriptional processes controlling cAMP production in cold-activated BAT as well as inguinal and epididymal white adipose tissue (iWAT and eWAT, respectively), we exposed chow diet-fed, male C57BL/6N mice for 24 h to 5 °C cold exposure (CE) and analysed transcriptome-wide gene expression changes using mRNA-seq. We combined our analysis with public mRNA-seq data from 72-h cold-exposed iWAT^31^ and observed significantly (false discovery rate ≤ 0.05) upregulated and downregulated genes in BAT, iWAT and eWAT (1,086 and 1,204, 804 and 448, and 584 and 71 genes, respectively; Fig. 1a–c, Extended Data Fig. 1a–c and Supplementary Table 1) were differentially regulated between cold and ambient room (22 °C) temperatures. Kyoto Encyclopedia of Genes and Genomes (KEGG) Gene Ontology (GO) analysis revealed that PPARG signalling and metabolic processes were induced, whereas HIF-1-dependent hypoxic signalling was repressed in cold-activated BAT (Fig. 1d). Canonical thermogenic and brown fat-associated GO terms like oxidative phosphorylation, thermogenesis, tricarboxylic acid cycle and carbon metabolism were upregulated in iWAT (Fig. 1e), whereas only a few GO terms were differentially regulated in eWAT (Fig. 1f), likely reflecting the limited thermogenic capacity in this depot. We next investigated the expression of cAMP-degrading and synthesizing enzymes: While expression of negative regulators of cyclic nucleotides, namely 3′,5′-cyclic nucleotide phosphodiesterases^32^, remained unchanged across all depots investigated (Extended Data Fig. 1d–f), mRNA levels of AC isoforms 3 and 4 (*Adcy3* and *Adcy4*) were induced in cold-exposed BAT (Fig. 1g), whereas only *Adcy3* expression was induced in iWAT (Fig. 1h), and *Adcy* isoform expression remained unaltered in eWAT (Fig. 1i). This pointed towards a specific role of mouse AC3 (mAC3) in thermogenic adipocytes and regulation of energy expenditure, as proposed previously^33,34^.

Analysis of public single-nuclei RNA-sequencing (snRNA-seq) datasets^35^ from BAs of male C57BL/6N mice revealed that *Adcy3* is lowly expressed under conditions of thermoneutrality (TN), which do not exhibit substantial NST. However, *Adcy3* expression is induced when lowering temperatures from TN to room temperature (RT; 22 °C) to CE in BAs, parallelling the increased expression of known BAT markers^36^, for example, *Ucp1*, *Ppargc1a*, pyruvate dehydrogenase kinase 4 (*Pdk4*), PR/SET domain 16 (*Prdm16*) and cell death inducing DFFA like effector A (*Cidea*; Fig. 1k). Thus, cold induces *Adcy3* expression in BAs and inguinal white adipocytes (iWAs).

## AC3 is required for cAMP biosynthesis in BAT and cold adaptation in obesity

**Fig. 2:**
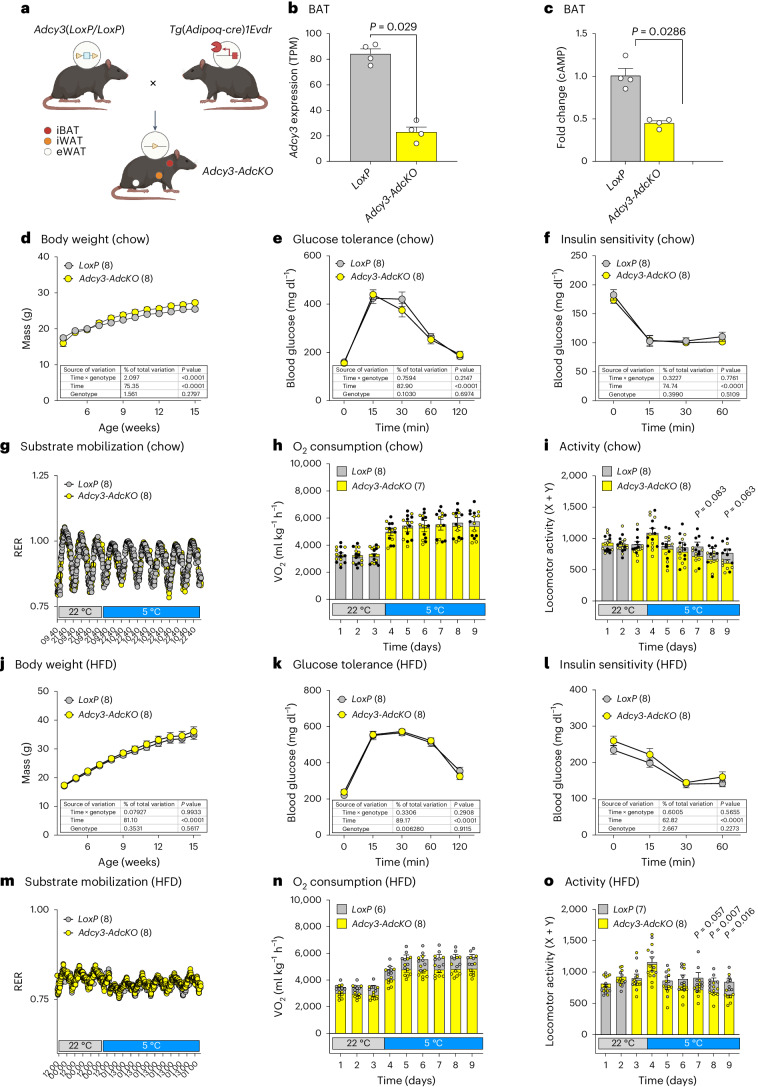
mAC3 is required for cAMP biosynthesis in BAT and required for cold adaptation in obesity. **a**, Breeding strategy to obtain pan-adipocyte-specific *Adcy3* knockout mice (*Adcy3-AdcKO*) and *LoxP* controls. Created with BioRender.com**b**, Quantification of *Adcy3* expression in BAT of chow diet-fed, male *LoxP* and *Adcy3-AdcKO* mice, determined by RNA-seq. **c**, Determination of cAMP levels in BAT of chow diet-fed, male *LoxP* and *Adcy3-AdcKO* mice at 22 °C, determined by ELISA and plotted as fold change. **b**,**c**, Bar graphs represent the mean + s.e.m. with all data points plotted. Unpaired, two-tailed, and non-parametric Mann–Whitney tests were performed to assess statistical significance. *P* values are indicated; *n* = 4. **d**, BW of chow diet-fed, male *LoxP* and *Adcy3-AdcKO* mice. **e**,**f**, Blood glucose levels during intraperitoneal glucose tolerance tests (**e**) and insulin tolerance tests (**f**) in chow diet-fed, male *LoxP* and *Adcy3-AdcKO* mice. **d**–**f**, Graphs represent the mean + s.e.m. Statistical significance was determined by performing two-way analysis of variance (ANOVA) with repeated measurements (mixed models). Post hoc *P*-value correction to account for multiple testing was performed using Bonferroni adjustment. The source of variation, percentage of the variation and exact *P* values are given in table insets; *n* = 8. Genotype *P* = 0.28 (**d**), *P* = 0.67 (**e**), *P* = 0.511 (**f**). **g**, Indirect calorimetry quantification of RERs in chow diet-fed, male *LoxP* and *Adcy3-AdcKO* mice (*n* = 8). **h**, Indirect calorimetry measurement of oxygen (O_2_) consumption in chow diet-fed, male *LoxP* (*n* = 8) and *Adcy3-AdcKO* (*n* = 7) mice. **i**, Quantification of mean daily locomotor activity in chow diet-fed, male *LoxP* (*n* = 8) and *Adcy3-AdcKO* (*n* = 8) mice. Bar graphs represent the mean + s.e.m. with all data points plotted. Parametric, unpaired, two-tailed Student’s *t*-tests were performed to assess statistical significance. *P* values are indicated. **j**, BW trajectories in HFD-fed, male *LoxP* and *Adcy3-AdcKO* mice. **k**,**l**, Blood glucose levels during intraperitoneal glucose tolerance tests (**k**) and insulin tolerance tests (**l**) in HFD-fed, male *LoxP* and *Adcy3-AdcKO* mice. **j**–**l**, Graphs represent the mean + s.e.m. Statistical significance was determined by performing two-way ANOVA with repeated measurements for *x* values (mixed models). Post hoc *P*-value correction to account for multiple testing was performed using Bonferroni adjustment. The source of variation, percentage of the variation and exact *P* values are given in table insets; *n* = 8. Genotype *P* = 0.562 (**j**), *P* = 0.912 (**k**) and *P* = 0.223 (**l**). **m**, Indirect calorimetry quantification of RERs in HFD-fed, male *LoxP* and *Adcy3-AdcKO* mice; *n* = 8. **n**, Indirect calorimetry measurement of oxygen consumption in HFD-fed, male *LoxP* and *Adcy3-AdcKO* mice; *n* = 8. **o**, Indirect calorimetry determination of mean daily locomotor activity in HFD-fed, male *LoxP* (*n* = 7) and *Adcy3-AdcKO* (*n* = 8) mice. Bar graphs represent the mean + s.e.m. with all data points plotted. Parametric, unpaired, two-tailed Student’s *t*-tests were performed to assess statistical significance. *P* values are indicated. Source data

**Extended Data Fig. 2:**
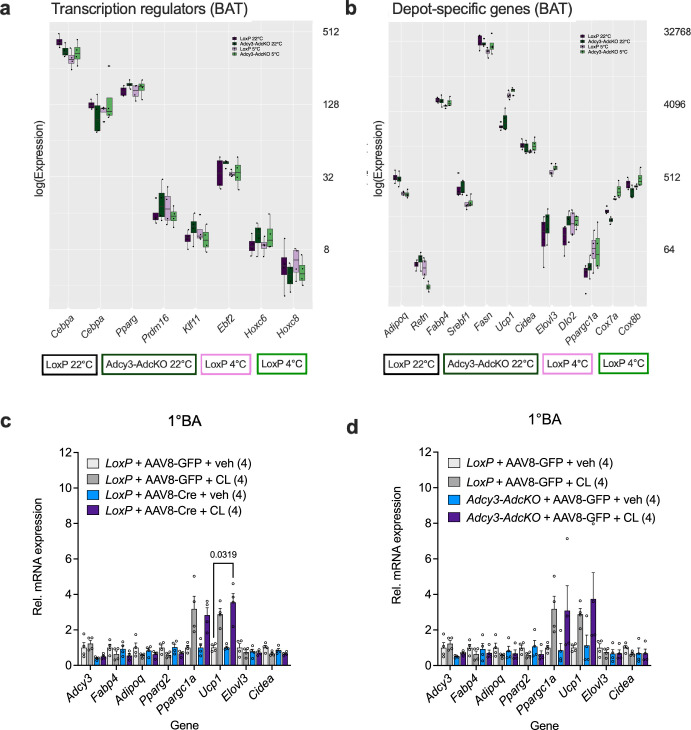
mAC3 is required for cAMP biosynthesis in BAT. mAC3 is required for cAMP biosynthesis in BAT and required for cold adaptation in cold. **a**,**b**, mRNA expression of transcriptional BAT regulators (**a**) and BAT-specific genes (**b**) in BAT from LoxP and Adcy3-AdcKO mice (at 22 °C or after 24 h of 5 °C cold exposure). Expression has been determined by mRNA-seq and depicted in transcripts per million (TPM); n = 4. The upper/lower whiskers represent largest/smallest observation less/greater than upper/lower hinge+1.5 × interquartile range (IQR). Central median represents 50% quantile. **c**,**d**, qPCR analysis of indicated BAT mRNA markers measured in 1°BA derived from SVF of LoxP or Adcy3-AdcKO mice, injected with 100,00 multiplicity of infection (MOI) of AAV8-CMV-Cre (AAV8-Cre) or AAV8-CMV-GFP (AAV-GFP), and stimulated for 6 h with 10 µM CL316,243 (CL), n = 4. Bar graphs represent mean + SEM with all data points plotted. To test for statistical significance, non-parametric (ranked) Kruskal–Wallis one-way analysis of variance (ANOVA) tests with Dunn’s correction for multiple testing was performed. Source data

To decipher the adipocyte-intrinsic roles of AC3 in vivo, we crossed mice harbouring *LoxP*-flanked *Adcy3* alleles^24^ with animals expressing adiponectin (*Adipoq*) promoter-driven Cre recombinase^37^ (*Adipoq*-cre) to obtain adipocyte-deficient *Adcy3* knockout mice (*Adcy3*^*LoxP/LoxP*^, *Adipoq*-cre^+/cre^; termed *Adcy3*-*AdcKO*) and cre-negative littermates (*Adcy3*^*LoxP/LoxP*^, *Adipoq*-cre^+/+^; termed *LoxP*; Fig. 2a). RNA-seq demonstrated a 60–70% reduction of *Adcy3* expression in *Adcy3-AdcKO* BAT with remaining *Adcy3* expression likely representing non-adipocyte cell populations in BAT^35^ (Fig. 2b). cAMP levels in BAT from mice housed at RT were reduced by around 50%, suggesting that mAC3 constitutes a pivotal cAMP regulator in BAT (Fig. 2c). To investigate the role of mAC3 in energy metabolism, we first analysed metabolic parameters in lean, chow diet-fed *Adcy3-AdcKO* and *LoxP* male mice. Although cAMP levels were reduced, we observed no discernible difference in body weight (BW; Fig. 2d), glucose tolerance (Fig. 2e) and insulin sensitivity (Fig. 2f). Also, indirect calorimetry revealed no differences in substrate mobilization (Fig. 2g) and oxygen consumption (Fig. 2h) between lean mice from both genotypes, and also the locomotor activity was not different (Fig. 2i). In line with these findings, gene expression of the transcriptional regulators of BAT formation and BAT-specific genes also remained largely unchanged (Extended Data Fig. 2a,b).

We next investigated thermogenic activation in mature, in vitro differentiated *LoxP* or *Adcy3*-deficient BAs from male mice, transduced with adeno-associated virus 8 encoding Cre recombinase (AAV8-Cre), followed by beta3-adrenergic agonist CL316,243 stimulation. This allowed us to compare BAs from *Adcy3-AdcKO* mice and acutely generated *Adcy3*-deficient BAs after Cre transduction. As control, we transduced the cells with AAV8-GFP virus. *Adcy3*-deficient and *LoxP* cells showed no difference in the expression of adipocyte identity markers, such as PPARG transcriptional isoform 2 (*Pparg2*) and fatty acid binding protein 4 (*Fabp4*), neither under basal conditions nor after beta-adrenergic treatment, despite *Adcy3* silencing in both (Extended Data Fig. 2c,d), suggesting unaltered BA formation and activation. Thus, adipocyte-specific loss of *Adcy3* does not affect thermogenesis and metabolic function in lean mice, despite controlling cAMP levels in BAT.

**Extended Data Fig. 3:**
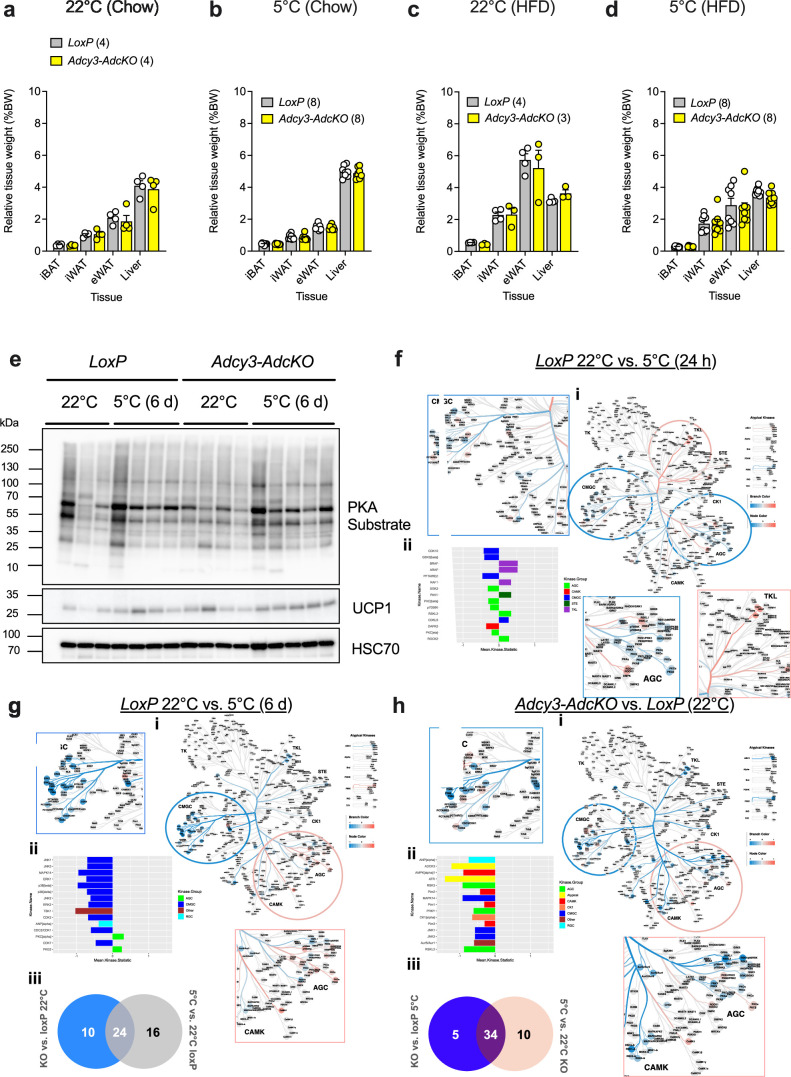
mAC3 is required for cAMP biosynthesis in BAT. mAC3 is required for cAMP biosynthesis in BAT and required for cold adaptation in cold. **a**–**d**, Relative (body-weight corrected) tissue weights from indicated adipose tissue depots and liver of LoxP (n = 4 for 22 °C, n = 8 for 5 °C) and Adcy3-AdcKO (n = 4 for 22 °C, n = 8 for 5 °C) mice either fed chow diet (**a**,**b**) or HFD (**c**,**d**) at 22 °C (**a**,**c**) or exposed for 24 h to 5 °C (**b**,**d**). Bar graphs represent mean + SEM with all data points plotted. Unpaired, two-tailed, and non-parametric Mann-Whitney tests were performed to assess statistical significance between both genotypes within each tissue. **e**, Western blot analysis of BAT from chow-diet fed, male LoxP (n = 3 for 22 °C, n = 4 for 5 °C) and Adcy3-AdcKO mice (n = 4 for 22 °C, n = 5 for 5 °C) at 22 °C or exposed to 5 °C for 6 days. Anti-phospho-PKA substrate antibodies were used for detection of PKA phosphorylation substrates. Anti-UCP1 antibodies probed for UCP1 protein levels. anti-HSC70 antibody served as loading control. **f**–**h**, Analysis of STK signaling activity in mouse BAT. Kinome trees depict the mean kinase statistic of differentially activated STKs measured by Pam Gene Array technology in LoxP at 22 °C compared to 5 °C for 24 h (**f**), LoxP at 22 °C compared to 5 °C for 6 days (**g**) and Adcy3-AdcKO compared to LoxP at 22 °C (**h**). **i**, Inserts show zoom-ins of relevant areas of the kinome tree (blue box: down-regulated kinases; red box: up-regulated kinases). **ii**, Mean kinase statistic of 15 most significantly differentially activated kinases is represented in bar graphs. **iii**, Venn diagram depicting differences in differentially activated kinases of the respective comparison. Data represents the mean of n = 4 biological replicates for all conditions. Source data

We and others reported that impediment of BAT function predisposes mice to increased BW gain and metabolic deterioration when feeding an obesogenic high-fat diet (HFD)^38–42^. Thus, we next tested if diet-induced obesity (DIO) could render *Adcy3*-*AdcKO* mice more susceptible to metabolic complications. However, even after HFD feeding, BW (Fig. 2j), adipose tissue weights at 22 °C and 5 °C (Extended Data Fig. 3a–d), glucose tolerance (Fig. 2k) and insulin sensitivity (Fig. 2l) remained unchanged. When performing indirect calorimetry, obese *Adcy3-AdcKO* mice displayed a reduced respiratory exchange ratio (RER; Fig. 2m) compared to lean mice (Fig. 2g), reflecting a transition in substrate mobilization from carbohydrate to lipid oxidation in DIO, yet no genotype-specific differences were observed. Of note, oxygen consumption (Fig. 2n) and locomotor activity (Fig. 2o) were reduced in obese *Adcy3*-*AdcKO* mice compared to *LoxP* controls. Thus, adipocyte-specific loss of *Adcy3* reduces energy expenditure in obese mice, particularly after additional cold stress.

## AC3 loss causes precocious activation of PKA-independent signalling pathways

Intrigued by the lack of thermogenic and metabolic dysfunction in lean *Adcy3-AdcKO* mice, we next asked whether *Adcy3* loss in adipocytes could cause an adaptative rewiring of cAMP signalling. To test if canonical cAMP-dependent kinase activities were altered in *Adcy3*-*AdcKO* BAT, we performed immunoblot activity profiling using PKA substrate-specific phospho-antibodies and quantified UCP1 protein expression. In line with lack of thermogenic dysfunction, PKA substrate phosphorylation and UCP1 levels in BAT after chronic cold were upregulated to the same degrees in *LoxP* and *Adcy3-AdcKO* mice (Extended Data Fig. 3e). To test more broadly the effects of *Adcy3* deficiency on signalling responses, we carried out serine/threonine kinase (STK) profiling using PamGene peptide arrays to globally infer differential STK kinase activities in *Adcy3-AdcKO* BAT. In *LoxP* mice, acute CE (24 h) elicited only mild overall STK signalling responses (Extended Data Fig. 3f), whereas chronic cold stimulation for 6 days decreased the activity of members of the CMGC kinase family (including cyclin-dependent kinases, glycogen synthase kinases and MAP kinases), and activity of AGC family members (including protein kinases A, C and G) was increased (Extended Data Fig. 3g). The CMGC family includes important hubs of cAMP-dependent signalling like the MAP kinases p38 (ref. ^43^), ERK^44^ and JNK^45^, which are typically engaged during acute thermogenic activation^46^. Strikingly, STK signatures in *Adcy3*-AdcKO BAT at 22 °C already resembled chronic CE in *LoxP* mice, except that PKA activity remained unchanged (Extended Data Fig. 3h). Thus, kinase responses regulating BAT activation were indeed altered in *Adcy3-AdcKO* mice but independent of PKA signalling.

## Active thermogenic adipocytes express a truncated AC3 (AC3-AT) transcript and protein isoform

**Fig. 3:**
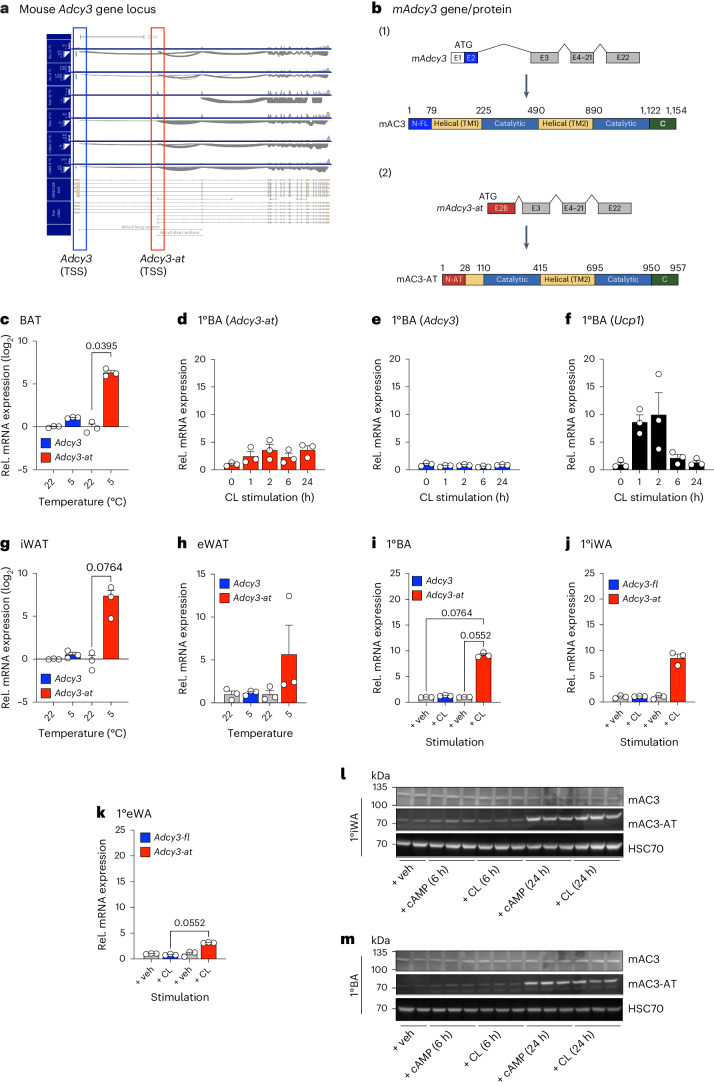
Activated thermogenic adipocytes express a truncated Adcy3 (Adcy-AT) transcript and protein isoform. **a**, Sashimi plots visualizing splicing junctions from aligned RNA-seq data in BAT in *Adcy3* from 20-week-old male C57BL/6N mice at 22 °C or after 24 h of 5 °C CE; *n* = 3–5. Illu, Illumina short-read RNA-seq; Telo, TeloPrime full-length cDNA-seq; cDNA, direct cDNA-seq. Reads were aligned against GENCODE M29 annotation and transcript reassembly using Illumina short-read and full-length RNA-seq using FLAIR75. **b**, Schematic of (1) canonical mouse *Adcy3* mRNA and mAC3 protein structure and (2) *Adcy3-at* transcript and mAC3-AT protein structure. Created with BioRender.com. **c**, Relative expression of *Adcy3* and *Adcy3-at* determined by qPCR analysis of BAT of male C57BL/6N mice housed at 22 °C or exposed to 5 °C for 24 h; *n* = 3. **d**–**f**, Relative expression of *Adcy3-at* (**d**), *Adcy3* (**e**) and *Ucp1* (**f**), determined by qPCR analysis, of 1°BAs stimulated for 1–24 h with 10 µM CL316,243 (CL, *n* = 3). **g**,**h**, Relative expression of *Adcy3* and *Adcy3-at* determined by qPCR analysis of iWAT (**g**) and eWAT (**h**) of male C57BL/6N mice housed at 22 °C or exposed to 5 °C for 24 h; *n* = 3. **i**–**k**, Relative expression of *Adcy3* and *Adcy3-at*, determined by qPCR analysis, in differentiated primary adipocytes derived from SVF cells isolated from BAT (1°BAs) (**i**), iWAT (1°iWAs) (**j**) and eWAT (1°eWAs) (**k**), and stimulated for 6 h with 10 µM CL. Replicates represent primary adipocytes isolated from individual mice (*n* = 3 mice). **c**–**k**, Bar graphs represent the mean + s.e.m. with all data points plotted. **c**,**g**–**k**, To test for statistical significance, non-parametric, ranked Kruskal–Wallis one-way ANOVA tests with Dunn’s correction for multiple testing were performed. *P* values are indicated. **l**,**m**, Western blot analysis of 1°BAs and 1°iWAs stimulated for 6 h and 24 h with 1 mM dibutyryl cAMP or 10 µM CL and analysed using a pan-AC3 antibody. Bands corresponding to mAC3 and mAC3-AT are from the same membrane but represent different exposure times. Heat shock protein 70 (HSC70) antibody was used as the loading control. The blots shown are representative of two independent experiments. Source data

**Extended Data Fig. 4:**
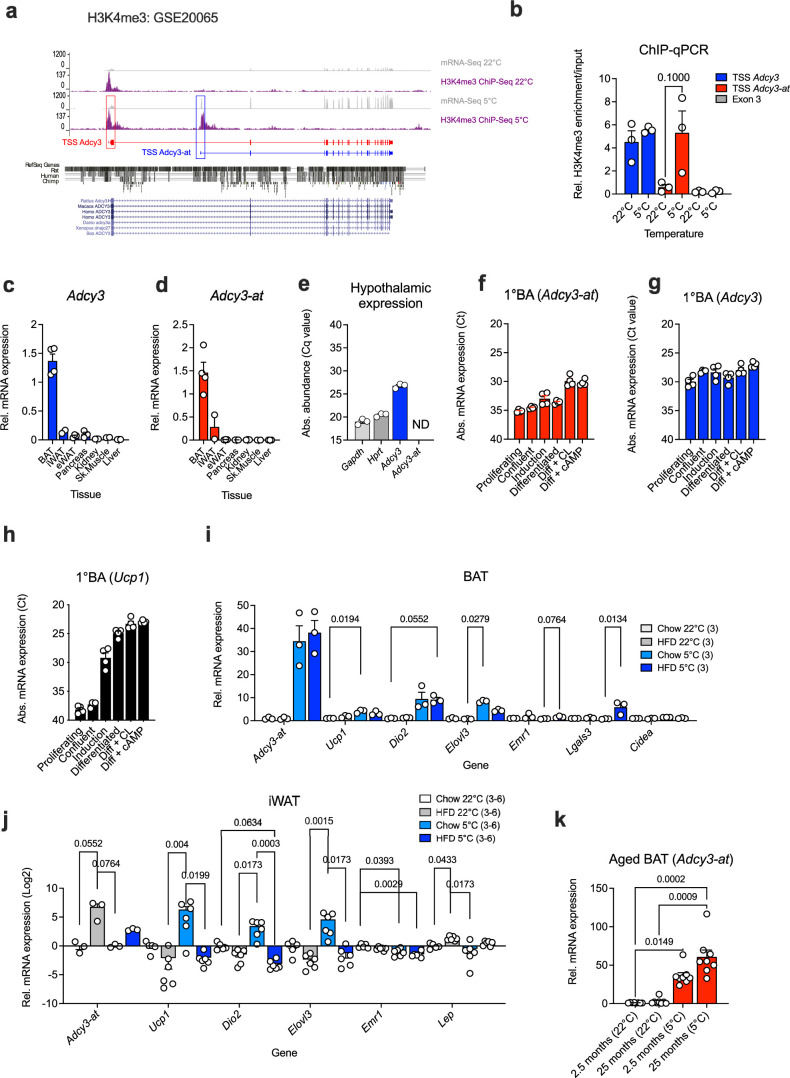
Activated adipocytes express truncated Adcy3. Activated thermogenic adipocytes express a truncated Adcy3 (Adcy-AT) transcript and protein isoform. **a**, UCSC genome browser tracks depicting changes in mRNA expression (grey) and H3K4me3 (magenta) in BAT from chow diet-fed, male C57BL/6N mice housed either at 22 °C or after 24 h of 5 °C cold exposure as determined by RNA-Seq (grey) and H3K4me3 ChiP-Seq (magenta). Red and blue transcripts depict contiguous Adcy3 and Adcy3-at transcripts, respectively, and Adcy3 isoform specific transcriptional start sites (TSS). **b**, ChIP-qPCR of H3K4me3 at the TSS of Adcy3 (blue), Adcy3-at (red) and exon 3, which is shared by Adcy3 and Adcy3-at (grey). Replicates represent H3K4me3 immunoprecipitations perform in individual animals (n = 3). Bar graphs represent mean + SEM with all data points plotted and unpaired, two-tailed, and non-parametric Mann-Whitney tests were performed to assess statistical significance. *P* values are indicated within the panel. **c**,**d**, Expression of Adcy3 (**c**) and Adcy3-at (**d**) in indicated tissues of chow diet fed, male C57BL/6N mice. Data points represent individual mice (n = 4 BAT, n = 2 iWAT, n = 3 eWAT, n = 3 pancreas, n = 3 kidneys, n = 2 skeletal muscle, n = 3 liver). **e**, Absolute expression (Cq value) of Adcy, Adcy3-at and housekeeping genes Hprt and Gapdh in hypothalamy of lean C57BL/6N male mice (n = 3 as determined by qPCR analysis. ND = Not detected. **f**–**h**, Absolute mRNA expression (Ct threshold levels) of Adcy3-at (**f**), Adcy3 (**g**) and Ucp1 (**h**) in BAT-derived SVF at indicated stages of brown adipogenesis and after stimulation with 1 mM db-cAMP or 10 µM CL316,243 as determined by qPCR. Replicates depict experiments performed in SVF isolated from individual mice (n = 4). Bar graphs represent mean + SEM with all data points. **i**,**j**, Expression of indicated BAT marker mRNAs in BAT (**i**) or iWAT (**j**) of chow diet or HFD-fed male C57BL/6N mice, each housed either at 22 °C or exposed to 24 h for 5 °C cold exposure as determined by qPCR. **k**, Expression of Adcy3-at in BAT of chow diet fed male C57BL/6N mice at 2.5 months or 25 months of age (n = 8). Bar graphs represent mean + SEM with all data points plotted. **i**–**k**, Bar graphs represent mean + SEM with all data points plotted. To test for statistical significance, non-parametric (ranked) Kruskal–Wallis one-way analysis of variance (ANOVA) tests with Dunn’s correction for multiple testing were performed. *P* values are indicated in the panel. Source data

Chromatin profiling by chromatin immunoprecipitation coupled to sequencing (ChIP–seq) allows genome-wide mapping of DNA-regulatory elements. Importantly, changes in cellular energetic activation and/or differentiation are parallelled by profound remodelling of promoter (trimethylated histone H3 Lys4 (H3K4me3)) and enhancer (acetylated histone H3 Lys27)-associated histone posttranslational modifications in BAs^47–49^. When interrogating published H3K4me3 data from BAT of 24-h cold-exposed male mice^50^, we observed a H3K4me3-marked promoter in intron 2 of the *Adcy3* gene that arose during cold (Extended Data Fig. 4a), which we verified by chromatin immunoprecipitation coupled to quantitative PCR (ChIP–qPCR; Extended Data Fig. 4b). This promoter marked a cold-dependent, new transcriptional start site giving rise to a 5′-truncated *Adcy3* mRNA isoform (Extended Data Fig. 4a), which we termed ‘*Adcy3-at*’ to discern it from GENCODE annotated full-length *Adcy3*. When combining Illumina short-read with Oxford Nanopore Technologies (ONT) full-length RNA-seq^28^, we validated that *Adcy3-at* represents a contiguous transcript spanning from the *Adcy3-at* transcription start site (TSS) to exon 2b-22 in cold-activated BAT (Fig. 3a). According to structural predictions, *Adcy3-at*-encoded mAC3-AT protein is N-terminally truncated compared to mAC3 (Fig. 3b) and distinct from hAC3 loss-of-function protein isoforms reported in human populations^17–19^. Thus, CE activates an intronic *Adcy3* promoter that produces a 5′-truncated, hitherto unknown *Adcy3-at* mRNA isoform in thermogenic fat.

When analysing our RNA-seq data separately for *Adcy3-at* and *Adcy3* isoform expression, we found that predominantly *Adcy3-at* was induced during CE, thus constituting the major upregulated *Adcy3* isoform in BAT after CE (Fig. 3c). In a cell-intrinsic manner, *Ucp1* and *Adcy3-at*, but not *Adcy3*, were induced after 1 h of CL316,243 treatment in primary brown adipocytes (1°BAs; Fig. 3d–f).

Other thermogenic depots like iWAT also induced *Adcy3-at* expression in cold (Fig. 3g), which was less pronounced in eWAT (Fig. 3h). Of note, other metabolic tissues did not express appreciable amounts of *Adcy3-at* or *Adcy3* (Extended Data Fig. 4c,d), suggesting predominant roles for mAC3-AT in thermogenic adipocytes. To investigate the role of mAC3-AT on brown adipogenesis, we first analysed *Adcy3* expression during adipocyte differentiation. *Adcy3-at* expression was low throughout adipogenesis of cells, isolated from the BAT stromal vascular fraction (SVF) of male mice, into BAs and was substantially upregulated after dibutyryl cAMP (db-cAMP) and CL316,243 stimulation, whereas *Adcy3* levels remained unchanged (Extended Data Fig. 4e,f). *Ucp1* expression was analysed as control (Extended Data Fig. 4g). When comparing the different fat depots, *Adcy3-at* expression was predominantly increased in 1°BAs (Fig. 3i) and primary inguinal white adipocytes (1°iWAs; Fig. 3j) but only slightly in primary epididymal white adipocytes (1°eWAs; Fig. 3k).

**Extended Data Fig. 5:**
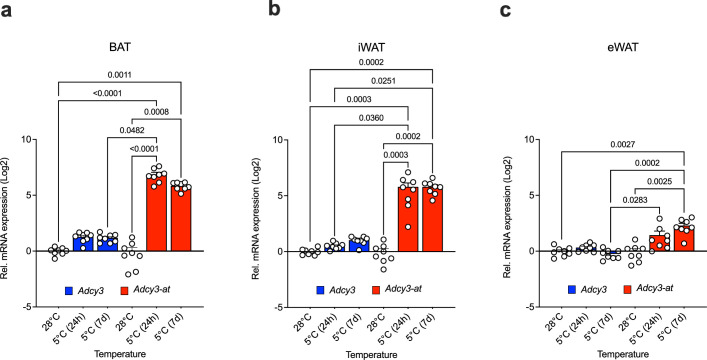
Activated adipocytes express truncated Adcy3. Activated thermogenic adipocytes express a truncated Adcy3 (Adcy-AT) transcript and protein isoform. **a**–**c**, Relative expression of Adcy (blue) and Adcy3-at (red) as determined by qPCR analysis in BAT (**a**), iWAT (**b**) and eWAT (**c**) from chow diet fed, male C57BL/6N mice housed either at 28 °C (thermoneutrality) or exposed to 5 °C for 24 h and 7 days (n = 8). Bar graphs represent mean + SEM with all data points plotted. To test for statistical significance, non-parametric (ranked) Kruskal–Wallis one-way analysis of variance (ANOVA) tests with Dunn’s correction for multiple testing were performed. *P* values are indicated in the panel. Source data

In mouse models of obesity-associated and aging-associated BAT dysfunction and inactivation, induction of *Adcy3-at* expression persisted in BAT of HFD-fed C57BL/6N male mice (Extended Data Fig. 4h), while cold-evoked *Adcy3-at* induction was blunted in iWAT of obese mice, a finding also observed for BAT markers like *Ucp1*, iodothyronine deiodinase 2 (*Dio2*) and ELOVL fatty acid elongase 3 (*Elovl3*; Extended Data Fig. 4i). Aged BAT from 25-month-old C57BL/6N male mice also expressed unaltered levels of *Adcy3-at* compared to young, 2-month-old mice (Extended Data Fig. 4j). Further, regarding mice transitioning from TN to cold temperatures, *Adcy3-at* expression was induced in BAT, iWAT and, to a lesser extent, in eWAT (Extended Data Fig. 5a–c). Thus, several mouse models of functional NST decline^51^ showed no differences in BAT but showed a blunted induction of iWAT *Adcy3-at* and other thermogenic markers.

The predicted amino acid sequences of mAC3-AT and mAC3 are in large parts identical but differ at the N terminus: mAC3-AT lacks the 146 amino acids of mAC3 and instead contains a unique 28 amino acid-long N terminus (Fig. 3b and Supplementary Table 6). This part contains the first block of transmembrane domains that constitute integral structural hallmarks and functional features of most ACs, suggesting that mAC3-AT specifically lacks the first transmembrane block (Fig. 3b). When performing immunoblots using a C-terminal AC3 antibody, we detected not only mAC3, but also an additional signal at 70 kD, which likely represented mAC3-AT, that only appeared after 24 h, but not 6 h, of db-cAMP or CL316,243 stimulation in 1°BAs and 1°WAs (Fig. 3l,m). Thus, beta-adrenergic and cold-mediated activation of thermogenic adipocytes induces expression of a new, intronic *Adcy3-at* promoter that encodes a truncated mAC3-AT protein 24 h after *Adcy3-at* induction.

## AC3-AT inhibits oxidative metabolism in vitro and in vivo

**Fig. 4:**
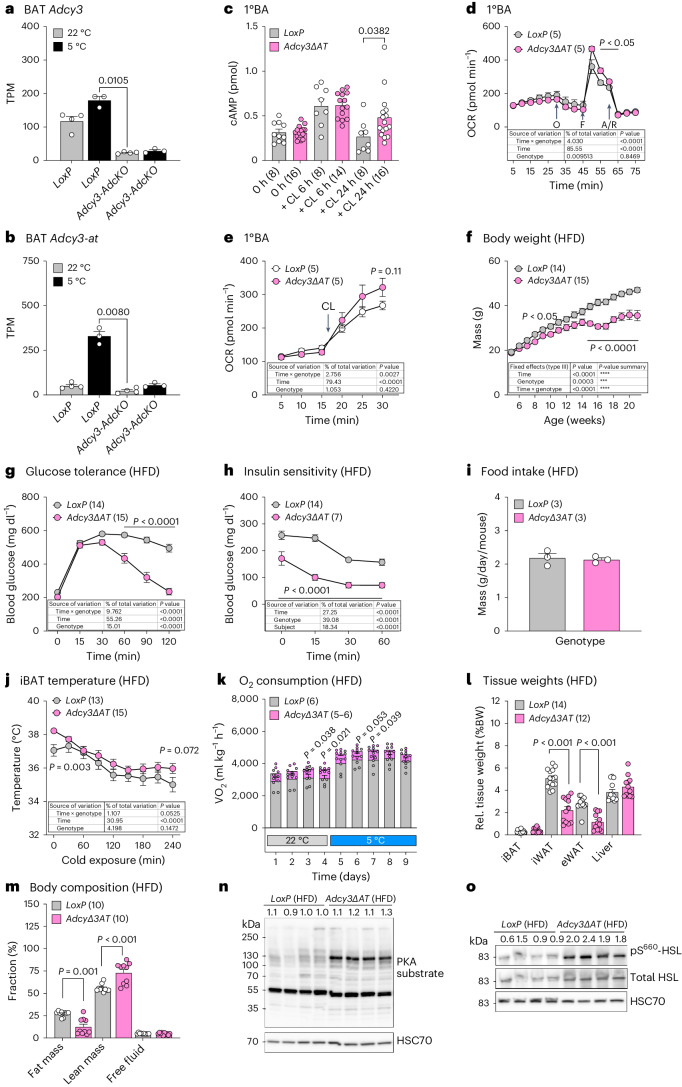
mAC3-AT inhibits oxidative metabolism in vitro and in vivo. **a**,**b**, Expression of *Adcy3* (**a**) and *Adcy3-at* (**b**) in BAT of chow diet-fed male mice housed at 22 °C (*n* = 4) and for 24 h at 5 °C (*n* = 3) as determined by RNA-seq. **c**, Quantification of intracellular cAMP levels in 1°BAs. 1°BAs, differentiated from the SVF of *LoxP* (*n* = 5) and *Adcy3-atΔKO* (*n* = 5) female and male mice, were stimulated with 10 μM CL for 6 or 24 h. Bar graphs represent the mean + s.e.m. with all data points plotted (*n* numbers indicated in parentheses). Statistical significance was determined using an unpaired, non-parametric and two-tailed Mann–Whitney test; *P* value is indicated. **d**,**e**, Oxygen consumption rate (OCR) in 1°BAs derived from the SVF of *LoxP* or *Adcy3∆AT* mice and stimulated with oligomycin (O), FCCP (F) and antimycin A plus rotenone (A/R) (**d**) or 1°BAs stimulated with 10 µM CL at the indicated time point (**e**); *n* = 5. Unpaired, two-tailed Student’s *t*-tests were performed to assess statistical significance. *P* values are indicated. **f**, BWs in HFD-fed, male *LoxP* (*n* = 14) and *Adcy3∆AT* (*n* = 15) mice. **g**, Blood glucose levels during intraperitoneal glucose tolerance tests in HFD-fed, male *LoxP* (*n* = 14) and *Adcy3∆AT* (*n* = 16) mice. **h**, Blood glucose levels during intraperitoneal insulin tolerance tests in HFD-fed, male *LoxP* (*n* = 14) and *Adcy3∆AT* (*n* = 7) mice. **f**–**h**, Graphs represent the mean + s.e.m. Statistical significance was determined by performing two-way ANOVA with repeated measurements for *x* values (mixed models). Post hoc *P*-value correction to account for multiple testing was performed using Bonferroni adjustment. The source of variation, percentage of the variation and exact *P* values are given in the table insets. Genotype *P* = 0.85 (**d**), *P* = 0.422 (**e**), *P* = 0.0003 (**f**), *P* = 0.0001 (**g**) and *P* = 0.0001 (**h**). **i**, Daily mean food intake in HFD-fed, male *LoxP* (*n* = 3) and *Adcy3∆AT* (*n* = 3) mice as determined using custom-made food hoppers averaged over 4 days. Unpaired, two-tailed and non-parametric Mann–Whitney tests were performed to assess statistical significance. **j**, iBAT proximal temperature measurements in HFD-fed, male *LoxP* (*n* = 13) and *Adcy3∆AT* (*n* = 15) mice exposed to 5 °C. Temperatures were recorded using implanted subdermal probes and telemetry devices. Genotype *P* = 0.147. **k**, Indirect calorimetry measurement of oxygen consumption in HFD-fed, male *LoxP* (*n* = 6) and *Adcy3∆AT* (*n* = 6) mice. **l**, Relative (BW adjusted) tissue weights from indicated adipose tissue depots and liver of *LoxP* (*n* = 14) and *Adcy3∆AT* (*n* = 12) mice fed a HFD and housed at 22 °C. **m**, Fractional body composition of HFD-fed *LoxP* (*n* = 10) and *Adcy3∆AT* (*n* = 10) mice, determined by NMR. **l**,**m**, Bar graphs represent the mean + s.e.m. with all data points plotted. Unpaired, two-tailed, and non-parametric Mann–Whitney tests were performed to assess statistical significance between genotypes within each tissue. **n**,**o**, Western blot analyses of BAT from HFD-fed male *LoxP* and *Adcy3* mice (*n* = 4) after 5 °C for 6 days. Anti-phospho-PKA substrate antibodies were used for detection of PKA phosphorylation substrates and anti-phospho-HS levels were normalized to total HSL protein. anti-HSC70 served as the loading control. Densitometric quantification was performed, and relative values are indicated above the blots. Source data

**Extended Data Fig. 6:**
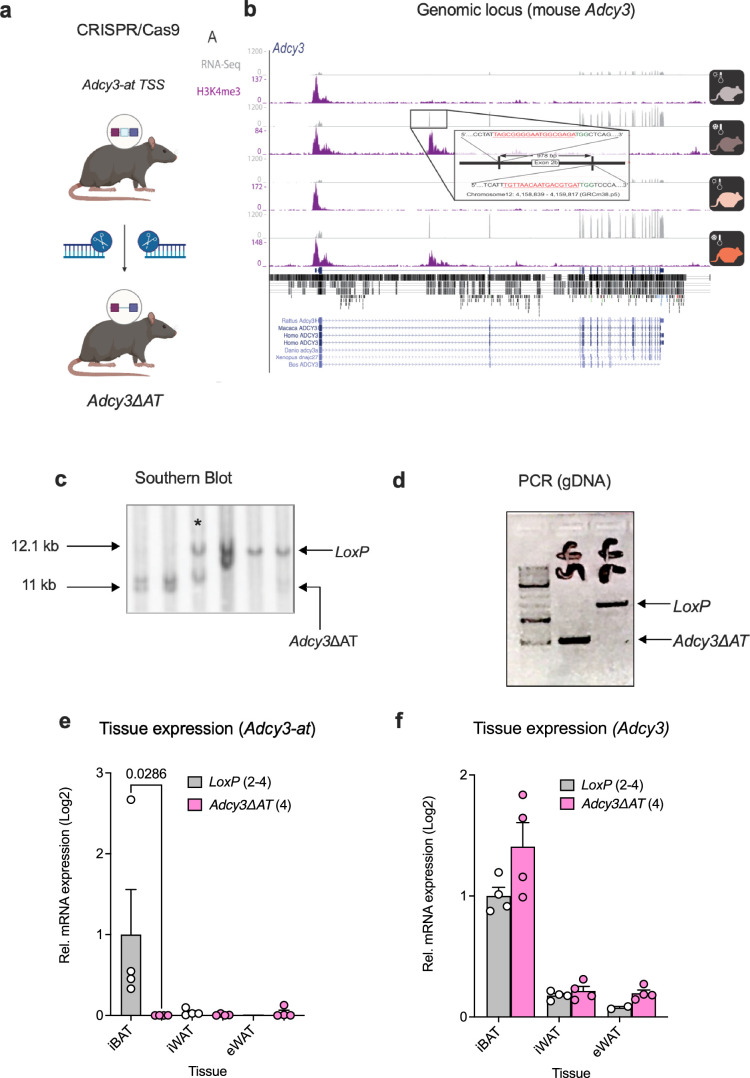
Generation of mice deficient for Adcy3-at. Generation of transgenic mice deficient for Adcy3-at at exon 1 and TSS using CRISPR/CAS9 gene. **a**, Illustration of CRISPR-Cas9 mediated excision of Adcy3-at specific Exon 2b, including Adcy3-at TSS (light blue), yielding Adcy3-at specific knockout (Adcy3∆AT) mice. **b**, USCS genome browser track depicting mRNA (grey) and H3K4me3 signals (magenta) in the murine Adcy3 locus from chow diet fed, male C57BL/6N mice either housed at 22 °C (light grey) and exposed to 5 °C for 24 h (dark grey) and from HFD-fed, male C57BL/6N mice housed at 22 °C (light orange) and exposed to 5 °C for 24 h (dark orange) as determined by RNA-Seq and H3 K4me3 ChiP-Seq. Location and sequence of sgRNAs used for CRISPR/Cas9 mediated 978-nt deletion of Adcy3-at TSS are indicated. **c**, Southern blot of a control (C57BL/6N wildtype) and from Adcy3∆AT embryonic stem cell (ESC) genomic DNA. The asterisk depicts a heterozygous Adcy3∆AT/wild-type ESC clone. **d**, PCR from genomic DNA isolated from wild-type and Adcy3∆AT ESC genomic DNA. **e**,**f**, Relative expression of Adcy3-at (**e**) and Adcy3 (**f**) as determined by qPCR analysis in BAT, iWAT, and eWAT from chow diet fed, male LoxP (n = 4 for BAT and iWAT, n = 2 for eWAT) and Adcy3∆AT mice (n = 4) housed at 22 °C. Bar graphs represent mean + SEM with all data points plotted. An unpaired, two-tailed, and non-parametric Mann-Whitney test was performed to assess statistical significance. *P* values are indicated within the panel. Created with BioRender.com. Source data

*Adcy3-AdcKO* mice do not allow us to specifically study the role of mAC3 versus mAC3-AT as those mice are deficient for both *Adcy3* isoforms (Fig. 4a,b). Thus, we aimed to delineate the specific roles for mAC3-AT in cellular and metabolic homeostasis of BAs in vivo and in vitro by generating *mAdcy3-at*-deficient transgenic mice: We performed CRISPR–Cas9-mediated gene deletion using single-guide RNAs (sgRNAs) flanking the H3K4me3-marked *Adcy3-at* promoter and excised a 978 base pairs (bp)-long DNA fragment including the *Adcy3-at* TSS in embryonic stem (ES) cells (Extended Data Fig. 6a,b). Excision of the *Adcy3-at* TSS and promoter region was confirmed by Southern blot (Extended Data Fig. 6c) and genomic PCR (Extended Data Fig. 6d). *Adcy3-at* null animals, termed *Adcy3∆AT*, exhibited no overt phenotypes and were fertile for both sexes, and *Adcy3∆AT* alleles were inherited at Mendelian ratios, thus arguing against confounding developmental defects following *Adcy3-at* deletion. As predicted, *Adcy3∆AT* mice were devoid of *Adcy3-at* (Extended Data Fig. 6e) but expressed *Adcy3* (Extended Data Fig. 6f).

**Extended Data Fig. 7:**
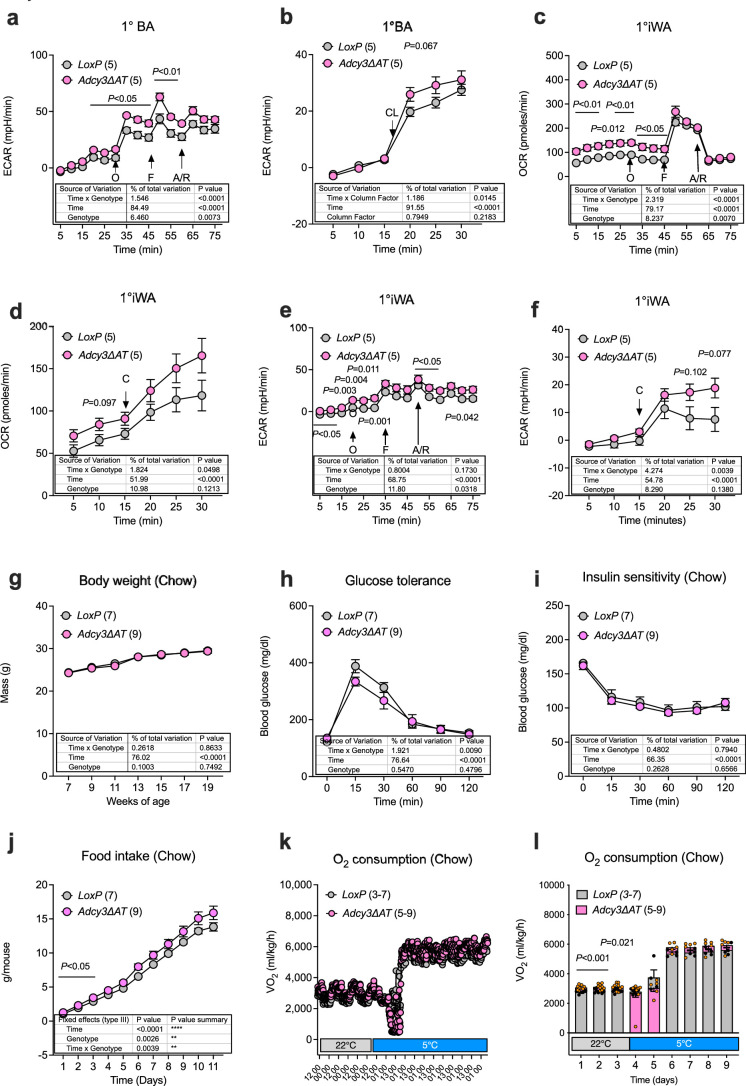
mAC3-AT inhibits oxidative metabolism and protects from obesity. mAC3-AT inhibits oxidative metabolism in vitro and in vivo and protects from diet-induced obesity. **a**,**b**, Extracellular acidification rates in 1°iBA derived from SVF of LoxP (n=5) or Adcy3∆AT (n = 5) and stimulated with oligomycin (O), FCCP (F) and Antimycin A plus Rotenone (A/R) (**a**), or 10 µM CL316,243 (CL) (**b**) at the indicated time points. The numbers of measured wells are indicated in the panel. **c**,**d**, Oxygen consumption rates in 1°iWA derived from SVF of LoxP (n = 5) or Adcy3∆AT (n = 5) and stimulated with oligomycin (O), FCCP (F) and Antimycin A plus Rotenone (A/R) (**c**) or 10 µM CL316,243 (CL) (**d**) at the indicated time points. The numbers of measured wells are indicated in the panel. **e**,**f**, Extracellular acidification rates in 1°iWA derived from SVF of LoxP (n = 5) or Adcy3∆AT (n = 5) and stimulated with oligomycin (O), FCCP (F) and Antimycin A plus Rotenone (A/R) (**e**), or 10 µM CL316,243 (CL) (**f**) at the indicated time points. The numbers of measured wells are indicated in the panel. **a**–**f**, Bar graphs represent mean + SEM. Unpaired, two-tailed Student’s T-tests were performed to assess statistical significance. *P* values are indicated within the panel. Genotype P = 0.0072 (**a**), P = 0.218 (**b**), P = 0.007 (**c**), P = 0.1213 (**d**), P = 0.032 (**e**), P = 0.138 (**f**). **g**, Body weights in chow diet-fed, male LoxP (n = 7) and Adcy3∆AT (n = 9) mice. **h**, Blood glucose levels during intraperitoneal glucose tolerance tests in chow diet-fed, male LoxP (n = 7) and Adcy3∆AT (n = 9) mice. **i**, Blood glucose levels during intraperitoneal insulin tolerance tests in chow diet-fed, male LoxP (n = 7) and Adcy3∆AT (n = 9) mice. **j**, Indirect calorimetry mediated quantification of cumulative food intake in chow diet-fed, male LoxP (n = 7) and Adcy3∆AT (n = 9) mice. **g**–**j**, Graphs represent mean + SEM. Statistical significance was determined by performing Two-Way ANOVA with repeated measurements for x values (mixed models). Post-hoc *P* value correction to account for multiple testing was performed using Bonferroni adjustment. The source of variation, % of the variation and exact *P* values are given in the table insets. Genotype P = 0.749 (**g**), P = 0.48 (**h**), P = 0.66 (**i**), P = 0.003 (**j**). **k**, Indirect calorimetry measurement of oxygen consumption in chow-diet fed, male LoxP (n = 7) and Adcy3∆AT (n = 9) mice. **l**, Indirect calorimetry determination of mean daily oxygen consumption in chow diet fed, male LoxP (n = 7) and Adcy3-AdcKO (n = 8) mice. Bar graphs represent mean + SEM with all data points plotted and parametric, unpaired, two-tailed Student’s t-tests were performed to assess statistical significance. *P* values are indicated within the panel. Source data

Next, we investigated whether loss of mAC3-AT affects cellular cAMP levels. cAMP levels were increased in 1°BAs after 24 h, but not 6 h, of CL316,243 stimulation (Fig. 4c), thereby parallelling the kinetics of mAC3-AT protein expression (Fig. 3l,m). These results suggest that mAC3-AT limits cAMP biogenesis and intracellular responses after prolonged beta-adrenergic stimulation. To study the cell-intrinsic roles of mAC3-AT in thermogenic adipocytes, we performed Seahorse bioenergetic analyses in 1°BAs and 1°iWAs and observed elevated maximal oxygen consumption rates after carbonyl cyanide-4(trifluoromethoxy)phenylhydrazone (FCCP) treatment in mitochondrial stress tests (Fig. 4d) and after CL316,243 stimulation (Fig. 4e) in *mAdcy3-at*-deficient adipocytes. Combined with higher extracellular acidification rates in Adcy3∆AT 1°BAs (Extended Data Fig. 7a,b), our results indicate that mAC3-AT regulates cellular metabolism through a combination of increased mitochondrial dissipation potential and glucose oxidation in Adcy3∆AT 1°BAs and 1°WAs (Extended Data Fig. 7c–f).

Next, we tested the role of mAC3-AT in regulating energy homeostasis in lean mice in vivo: Although BWs remained unchanged (Extended Data Fig. 7g), *Adcy3∆AT* male mice exhibited slightly improved glucose tolerance (Extended Data Fig. 7h), whereas insulin sensitivity remained unchanged (Extended Data Fig. 7i) and food intake was slightly increased (Extended Data Fig. 7j), suggesting negative energy balances in *Adcy∆AT* mice. In line with this, *Adcy3∆AT* mice exhibited increased oxygen consumption during ambient temperatures (Extended Data Fig. 7k,l).

**Extended Data Fig. 8:**
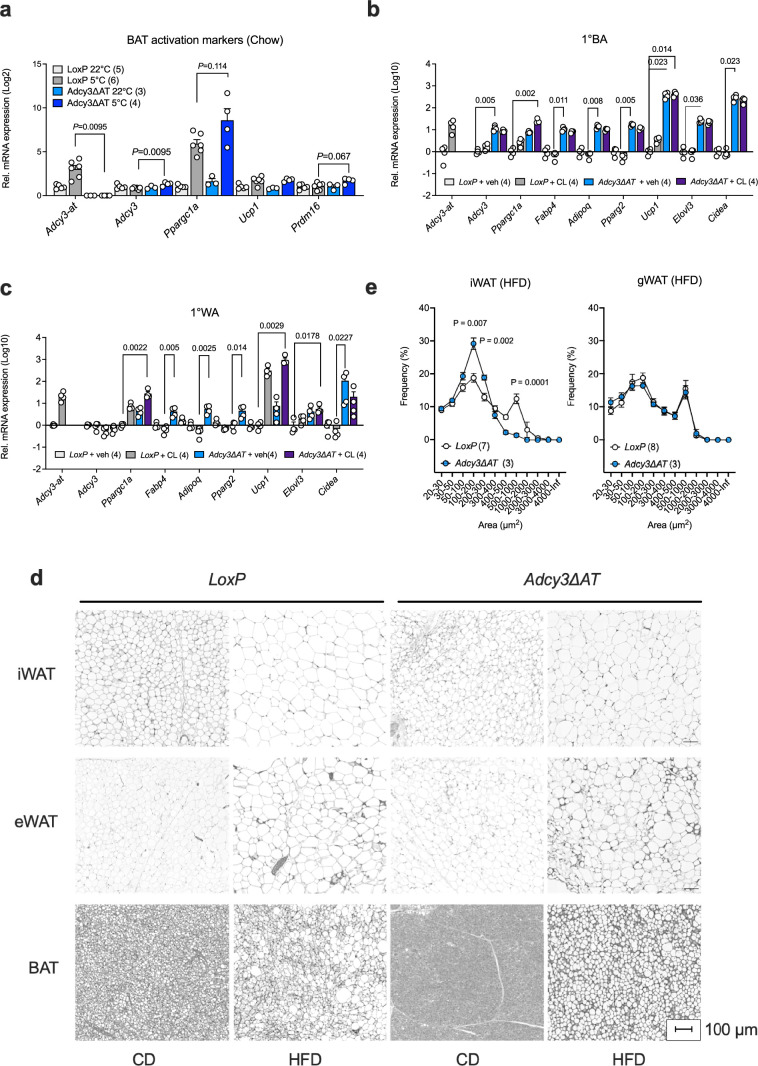
mAC3-AT inhibits oxidative metabolism and protects from obesity. mAC3-AT inhibits oxidative metabolism in vitro and in vivo and protects from diet-induced obesity. **a**, relative expression of indicated thermogenic BAT marker mRNAs in chow diet-fed, male LoxP (n = 5 for 22 °C, n = 6 for 5 °C) and Adcy3∆AT (n = 3 for 22 °C, n = 4 for 5 °C) mice. Bar graphs represent mean + SEM with all data points plotted and unpaired, two-tailed, non-parametric Mann-Whitney tests were performed for indicated comparisons to assess statistical significance. *P* values are given within the panel. **b**,**c**, Relative expression of indicated thermogenic BAT marker mRNAs in 1°BA (**b**) and 1°iWA (**c**) from LoxP (n = 4 individual SVF preparations) and Adcy3∆AT (n = 4 individual SVF preparations). Primary adipocytes were stimulated with 10 μM CL316,243 for 6 h. Bar graphs represent mean + SEM with all data points plotted. To test for statistical significance, non-parametric, ranked Kruskal–Wallis one-way analysis of variance (ANOVA) tests with Dunn’s correction for multiple testing were performed. *P* values are indicated in the panel. **d**, Images of HE-stained adipose tisue, extracted from male LoxP and Adcy3ΔAT mice fed CD or HFD. Scale bars: 100 µm. **e**, Adipocyte size distribution quantified using images as shown in (**d**). iWAT adipocytes size distribution was analyzed for n = 7 LoxP and n = 3 Adcy3ΔAT animals. gWAT adipocyte size distribution was analyzed for n = 8 LoxP and n = 3 Adcy3ΔAT animals. Mean ± SD of the distributions from all mice per group; *P* values indicated for unpaired, two-sided t tests with Welch correction between LoxP and Adcy3ΔAT at the size range; *p ≤ 5 × 10–2; **p. Source data

To test for a BAT-specific involvement in the improved metabolism seen in *Adcy3∆AT* mice, we performed qPCR analysis and observed trends towards increased *Ppargc1a* and *Prdm16* expression, suggesting elevated thermogenic activation of cold-acclimatized *Adcy3∆AT* BAT (Extended Data Fig. 8a). Regarding cell-intrinsic mechanisms, absence of mAC3-AT caused precocious expression of BAT activity markers, such as *Ppargc1a*, *Fabp4*, *Adipoq*, *Pparg2*, *Ucp1*, *Elovl3* and *Cidea* in 1°BA and 1°iWA thermogenic adipocytes (Extended Data Fig. 8b,c). Based on this profound thermogenic gene induction in *Adcy3-at*-deficient primary adipocytes, we hypothesized that loss of mAC3-AT might protect against obesity and obesity-induced metabolic alterations due to precocious BAT activation already under RT. To test this, we subjected *LoxP* and *Adcy3∆AT* male mice to HFD feeding and observed that *Adcy3∆AT* were indeed refractory to HFD-induced BW gains (Fig. 4f). Concomitantly, *Adcy3-at*-deficient animals exhibited improved glucose tolerance (Fig. 4g), reduced glucose levels during intraperitoneal insulin challenges (Fig. 4h), no difference in food intake (Fig. 4i), increased BAT thermogenic activity as measured using subdermal thermometry telemetry probes (Fig. 4j) and increased oxygen consumption at 22 °C and during 4 °C of CE, supporting the notion of ADCY3-AT serving as a negative regulator of energy expenditure. Alongside the pronounced BW loss, iWAT and eWAT but not liver and BAT tissue weights were reduced in *Adcy3∆AT* mice (Fig. 4k), which was parallelled by selective loss of fat, but not lean or fluid mass, as determined by nuclear magnetic resonance (NMR; Fig. 4l). H&E staining across *Adcy3∆AT* and *LoxP* adipose tissue depots followed by automated cell morphometric analyses revealed a reduction in adipocyte size in iWAT, but not eWAT, corroborating the selective role for mAC3-AT in thermogenic fat depots (Extended Data Fig. 8d,e). Thus, *mAdcy3-at* deficiency improves energy metabolism in vitro and in vivo, especially during DIO.

## mAC3-AT alters AC3 subcellular localization and thereby limits cAMP biosynthesis

**Extended Data Fig. 9:**
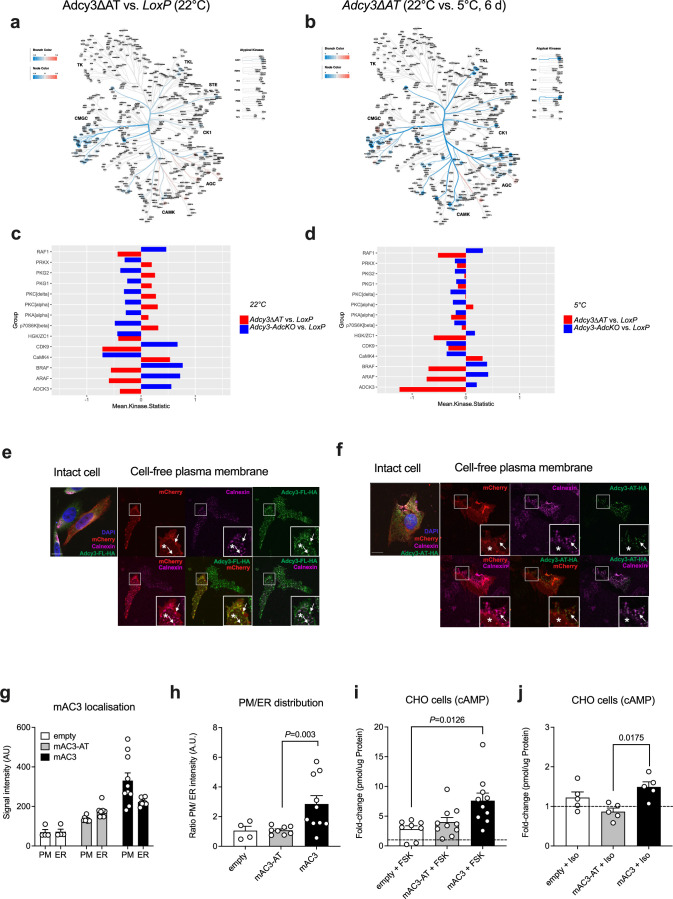
mAC3-AT alters mAC3 localization and limits cAMP. mAC3-AT alters mAC3 subcellular localization and, thereby, limits cAMP bio synthesis. **a**,**b**, analysis of STK activity in Adcy3∆AT BAT. Kinome trees depict the mean kinase statistic of differentially activated STKs in Adcy3 vAT versus LoxP (22 °C) (**a**) and Adcy3∆AT (22 °C) versus Adcy3∆AT (5 °C, 6 days) (**b**). **c**,**d**, Comparison of Adcy3∆AT versus LoxP at 22 °C (**c**) and Adcy3∆AT versus LoxP after 6 days at 5 °C (**d**). At 22 °C, a set of kinases is inversely regulated in BAT of Adcy3∆AT and Adcy3AdcKO mice compared to LoxP mice. The mean kinase statistic of these kinases is shown for 22 °C (**c**) and for 5 °C (**d**). Data includes n = 4 biological replicates for all conditions. **e**, CHO cells stably expressing AC3-HA and transiently transfected with mCherry-CAAX were sonicated and labeled with a Calnexin antibody (ER-marker, magenta) and an HA antibody (green). mCherryCAAX fluorescence is indicated in red. Single channels are shown at the top, merged images at the bottom row. White boxes are shown as magnified view at the bottom-right. Arrows point to endoplasmic reticulum-plasma membrane (ER-PM) contact sites, asterixis indicate contact-free parts within the membrane sheet. Scale bar: 10 μm. **f**, See (**e**) for CHO cells stably expressing AC3-AT-HA. The images represent at least four independent experiments, with 5-10 cells analysed per condition per experiment. **g**, Quantification of the average HA-signal intensity within the PM (co-localization with mCherry-CAAX) and the ER (co-localization with calnexin). Data shown as mean + SEM (empty vector: n = 4; AC3-AT: n = 8, AC3: n = 10). **h**, Ratio PM/ER distribution (empty vector: n = 4; mAC3-AT: n = 8, mAC3: n = 10). Bar graphs represent mean + SEM with all data points plotted. An unpaired, two-tailed, and non-parametric Mann-Whitney tests were performed to assess statistical significance; p-values are indicated within the panel. **i**,**j**, Quantification of intracellular cAMP levels. CHO cells stably expressing mAC3-AT or mAC3, or empty vector transfected CHO cells were stimulated with buffer or 2 μM Forskolin for 30 min (**i**) or buffer or 1 μM Isoproterenol for 15 min (**j**). Values were normalized to the respective buffer control and to the respective amount of total protein (empty vector: n = 4; AC3-AT: n = 5, AC3-FL: n = 5). **g**–**j**, Data is shown as mean + SEM with all data points plotted. To test for statistical significance, non-parametric (ranked) Kruskal–Wallis one-way analysis of variance (ANOVA) tests with Dunn’s correction for multiple testing were performed; p-values are indicated in the panel. Source data

Our in vitro and in vivo data indicated that solely deleting expression of the cold-induced mAC3-AT protein isoform is sufficient to increase oxidative metabolism and thermogenic activation of BAs. To understand the underlying molecular mechanisms, we first analysed STK activities using PamGene Array in BAT from *Adcy3∆AT* versus *LoxP* mice. At 22 °C, *Adcy3∆AT* BAT exhibited an activation of thermogenesis-associated AGC kinase family members such as PKA, PKC and PKG, presumably reflecting an ectopic activation of thermogenesis after mAC3-AT loss already at RT (Extended Data Fig. 9a); this phenomenon was not further exaggerated by additional CE (Extended Data Fig. 9b). When juxtaposing patterns of STK kinase activation between BAT from *Adcy3∆AT* and *Adcy3-AdcKO* mice, ACG kinases were inversely activated, suggesting that deleting both mAC3 and mAC3-AT proteins evokes opposing effects as compared to ablating mAC3-AT alone (Extended Data Fig. 9c,d).

**Fig. 5:**
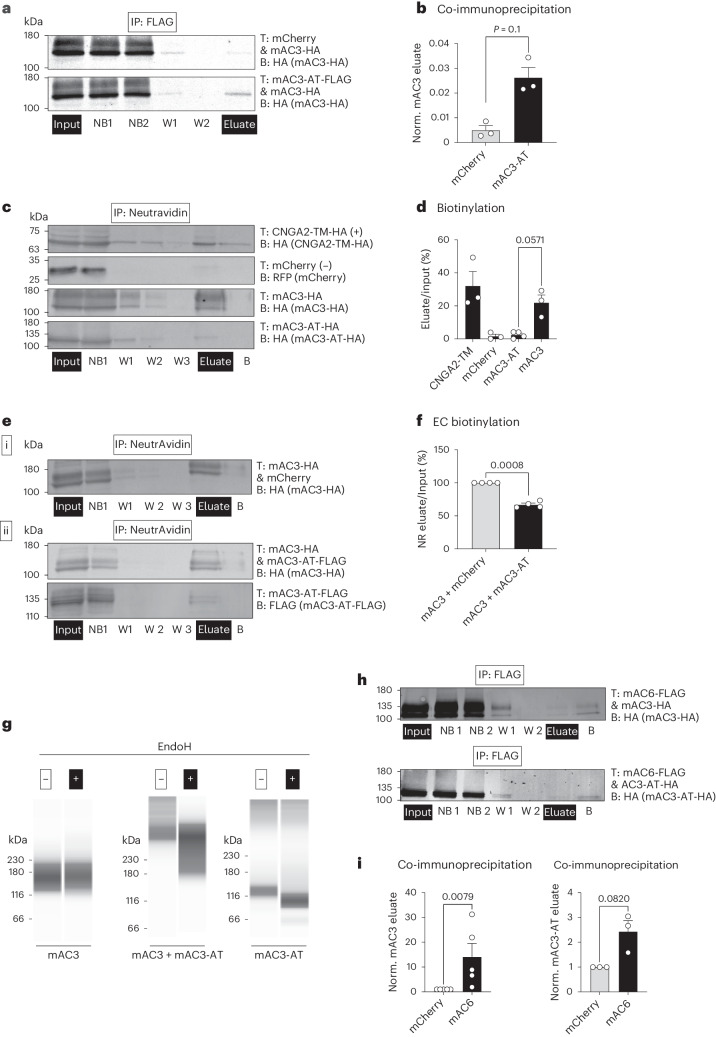
mAC3-AT alters mAC3 subcellular localization and, thereby, limits cAMP biosynthesis. **a**, Co-immunoprecipitation of mAC3/mAC3-AT in CHO cells. CHO cells stably expressing AC3-HA were transiently transfected (T) with mCherry (upper row) or AC3-AT-FLAG (bottom row). Total protein lysates were incubated with anti-FLAG magnetic beads, purified, and the different fractions were analysed by western blot using an HA antibody (B) (input; NB, non-bound; W, wash; eluate). **b**, Quantification of immunoblots. The ratio of mAC3 protein density in the eluate compared to the total protein input for each condition (mCherry and mAC3-AT-FLAG, *n* = 3 per condition) was determined from **a**. Bar graphs represent the mean + s.e.m. with all data points plotted. An unpaired, two-tailed and non-parametric Mann–Whitney test was performed to assess statistical significance. The *P* value is indicated. **c**, Western blot analysis of biotinylation assay. Total protein lysates isolated from CHO cells transfected with CNGA2-TM, mCherry, mAC3-HA and mAC3-AT-HA. Cells were treated with the biotinylation reagent sulfo-NHS-SS-biotin, lysed (input) and purified using a NeutrAvidin-agarose-resin (NB; W; eluate; B, beads). Immunoblots were incubated with a CNGA2, HA or RFP (mCherry) antibody (B). **d**, Quantification biotinylation assay for single transfections shown in **c** using densitometric analysis of immunoblots. Values were normalized to the respective input sample. The ratio of eluate to input as a percentage is shown as the mean + s.e.m. with all data points plotted. An unpaired, two-tailed and non-parametric Mann–Whitney test was performed to assess statistical significance. The *P* value is indicated; *n* = 3. **e**, Western blot analysis of biotinylation assay for mAC3/mAC3-AT. Total protein lysates isolated from CHO cells transfected (T) with AC3-HA alone (i) or together with AC3-AT-FLAG (ii). Cells were treated with the biotinylation reagent Sulfo-NHS-SS-Biotin, lysed (input) and purified using a NeutrAvidin-agarose-resin (NB, W, eluate, B). Immunoblots were incubated with an HA or FLAG antibody (B). **f**, Quantification of biotinylation assay AC3-HA/AC3-AT using densitometric analysis of immunoblots shown in **e**. Values were normalized to the respective input sample and to the mAC3/mCherry control. Data are shown as a percentage as the mean + s.e.m. with all data points plotted. A paired, two-tailed Student’s *t*-test was performed. The *P* value is indicated; *n* = 4. **g**, EndoH digestion assay. CHO cells expressing mAC3 (HA), mAC3-AT (HA) or both (mAC3-HA/mAC3-AT-FLAG) were subjected to EndoH treatment and analysed by a capillary immunoassay system using an HA antibody, thereby detecting the individual proteins in the single transfections and mAC3-HA in the double transfection. **h**, Co-immunoprecipitation of mAC3 or mAC3-AT with mAC6 in CHO cells. CHO cells stably expressing mAC3-HA or mAC3-AT were transiently transfected (T) with mAC6. Total protein lysates were incubated with anti-FLAG magnetic beads, purified, and the different fractions were analysed by western blot using an HA antibody (B) (input, NB, W, eluate, B). **i**, Quantification of western blot analyses. The ratio of mAC3 or mAC3-AT protein density in the eluate compared to the total protein input for both conditions (mCherry and mAC6) was determined from **h**. Bar graphs represent the mean + s.e.m. with all data points plotted. A paired, two-tailed and non-parametric Mann–Whitney test was performed to assess statistical significance. The *P* values are indicated; *n* = 3–5. IP, immunoprecipitation. Source data

Both mAC3-AT and mAC3 are expressed in cold-activated BAT (Fig. 4a,b) and mAC3-AT dampens beta-adrenergic receptor-dependent cAMP biosynthesis (Fig. 4c). However, the underlying molecular and cellular mechanism remains elusive. Transmembrane ACs assemble as homodimers but can also form heterodimers with other AC isoforms^52^. We hypothesized whether mAC3-AT forms heterodimers with mAC3 and could thereby affect the magnitude of mAC3-evoked cAMP synthesis. To test this in vitro, we coexpressed FLAG-tagged mAC3-AT and HA-tagged mAC3 in Chinese Hamster Ovary (CHO) cells and demonstrated that mAC3-AT and mAC3 indeed interact (Fig. 5a,b). The N terminus is essential for controlling AC translocation to the PM, thereby regulating AC localization and, concomitantly, enzymatic function^53^. As mAC3-AT lacks the N-terminal domain and interacts with mAC3, we wondered whether mAC3-AT is intrinsically unable to translocate to the PM and, additionally, might even impair PM translocation of mAC3. To this end, we performed biotinylation assays to selectively label transmembrane proteins facing the extracellular environment: When individually overexpressing the two different AC3 isoforms, only mAC3, but not mAC3-AT, was biotinylated (Fig. 5c,d), demonstrating that mAC3-AT is incapable of localizing to the PM. Importantly, when coexpressing both mAC3-AT and mAC3, mAC3 biotinylation was reduced (Fig. 5e,f), demonstrating that expression of mAC3-AT impairs PM transport of mAC3. We confirmed these results by testing the sensitivity of mAC3-AT/mAC3 to endoglycosidase H (EndoH) digestion, an enzyme that selectively degrades asparagine-linked mannose-rich simple oligosaccharides, but not highly processed complex oligosaccharides from glycoproteins. This assay allowed us to indirectly monitor protein trafficking through the endoplasmic reticulum (ER) and Golgi, as EndoH cleaves oligosaccharides added in ER, but as soon as glycosylated proteins are further processed in the Golgi, they become resistant to EndoH treatment^54^. We subjected mAC3 and mAC3-AT proteins to EndoH treatment and revealed that mAC3, but not mAC3-AT, is resistant to EndoH (Fig. 5g). Furthermore, after coexpression of both isoforms, mAC3 also became sensitive to EndoH treatment (Fig. 5g), indicating that indeed mAC3-AT is retained in the ER and sufficient to sequester mAC3 when being coexpressed.

To complement our mechanistic insights with image-based approaches, we subjected CHO cells grown on glass coverslips to short ultrasound pulses, whereby the upper part of cells was removed, and PM and its associated complexes remained intact as membrane sheets (‘unroofing’)^55^. As a positive control for visualizing the PM compartment, we expressed membrane-anchored mCherry. mAC3 colocalized with mCherry at the PM but showed little overlap with calnexin, a protein residing in the ER membrane, marking ER–PM contact sites. In contrast, mAC3-AT showed no colocalization with mCherry but colocalized with calnexin, demonstrating that mAC3-AT is retained in ER (Extended Data Fig. 9e–h).

To probe if mAC3-AT intrinsically possesses catalytic activity, we determined cAMP levels by ELISA in CHO cells overexpressing the individual mAC3 isoforms. As expected mAC3, but not mAC3-AT activity was stimulated by forskolin, a direct transmembrane AC activator (Extended Data Fig. 9i), and by isoproterenol, a beta-adrenergic receptor agonist, which stimulates AC activity via G proteins (Extended Data Fig. 9j). These results indicate that mAC3-AT does not possess enzymatic activity or lose its interaction with G proteins due to altered subcellular localization. As cAMP levels in mAC3-AT-overexpressing cells after isoproterenol stimulation were lower compared to control cells, we next asked whether mAC3-AT can also interact with other AC isoforms, thereby controlling cAMP synthesis beyond AC3. Apart from AC3, murine BAT expressed AC6 (Fig. 1g). To test if mAC3-AT also interacts with mAC6, we coexpressed mAC3 or mAC3-AT with mAC6. The results showed that mAC6 interacted with both mAC3 and mAC3-AT (Fig. 5h,i), indicating that mAC3-AT not only controls AC3 but also can globally regulate other AC isoforms.

## hAC3-AT expression, interaction with hAC3 and sequestration in the ER are conserved in humans

**Fig. 6:**
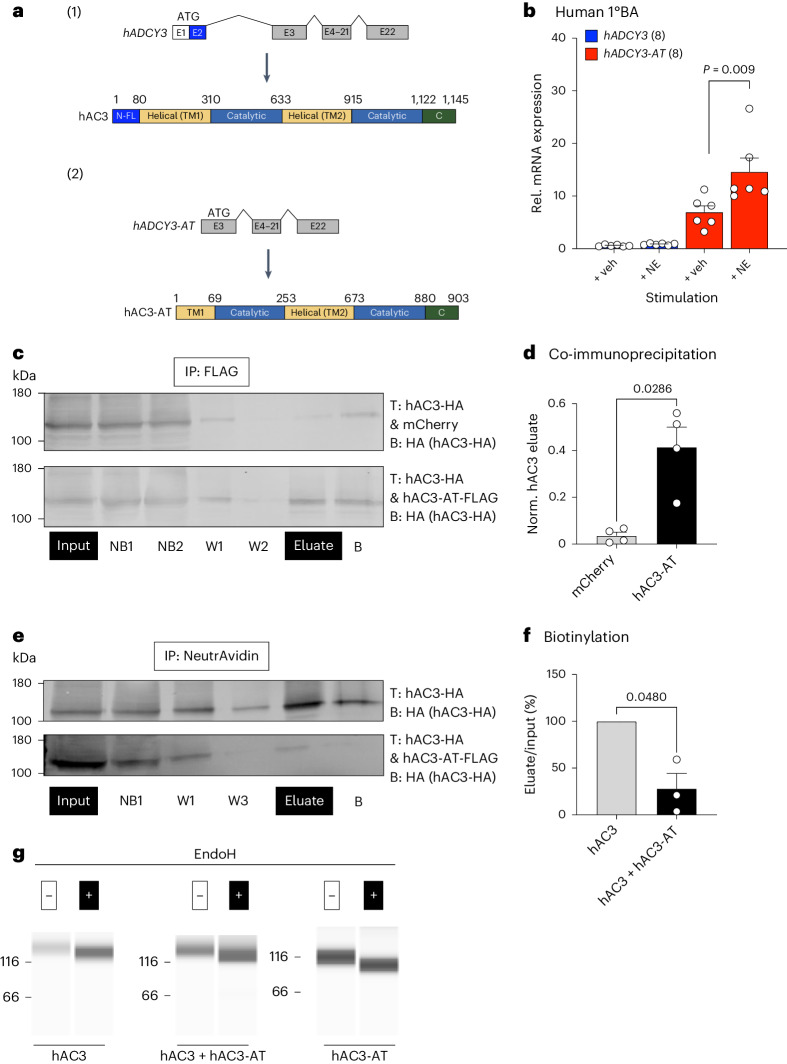
hAC3-AT expression, interaction with AC3 and sequestration in the ER are conserved in humans. **a**, Schematic of full-length *hADCY3* transcript and hAC3 protein structure (1) and *hADCY3-AT* transcript and hAC3-AT protein structure (2). Created with BioRender.com. **b**, Expression of *hADCY3-AT* in human 1°BAs derived from SVF precursors and stimulated with 1 μM NE for 16 h (*n* = 8 per condition). An unpaired, two-tailed and non-parametric Mann–Whitney test was performed to assess statistical significance. Data are shown as a percentage as the mean + s.e.m. with all data points plotted. The *P* value is indicated. **c**, Co-immunoprecipitation of hAC3/hAC3-AT in HEK293T cells: HEK293T cells were transiently transfected (T) with hAC3-HA and mCherry or hAC3-FLAG. Total protein lysates were incubated with anti-FLAG magnetic beads, purified, and the different fractions were analysed by western blot using an HA antibody (B) (input, NB, W, eluate, B). **d**, Quantification of immunoblot analyses. The ratio of hAC3 protein density in the eluate compared to total protein input for both conditions (mCherry and hAC3-AT-FLAG) was determined from **c**. Bar graphs represent the mean + s.e.m. with all data points plotted. An unpaired, two-tailed and non-parametric Mann–Whitney tests were performed to assess statistical significance; *P* value is indicated; *n* = 4. **e**, Western blot analysis of biotinylation assay for hAC3/hAC3-AT. Total protein lysates isolated from CHO cells transfected (T) with AC3-HA alone or together with AC3-AT-FLAG. Cells were treated with the biotinylation reagent Sulfo-NHS-SS-Biotin, lysed (input), and purified using a NeutrAvidin-agarose-resin (NB, W, eluate, B). Immunoblots were incubated with an HA or FLAG antibody (B). **f**, Quantification biotinylation assay hAC3-HA/hAC3-AT using densitometric analysis of immunoblots shown in **e**. Values were normalized to the respective input sample and to hAC3 only. Bar graphs represent the mean + s.e.m. with all data points plotted. A paired, two-tailed and parametric Student’s *t*-test was performed to assess statistical significance. The *P* value is indicated; *n* = 3. **g**, EndoH digestion assay. HEK293T cells expressing hAC3 (HA), hAC3-AT (FLAG) or both (hAC3-HA/hAC3-AT-FLAG) were subjected to EndoH treatment and analysed by a capillary immunoassay system using an HA antibody (double transfection) or the AC3 antibody (single transfections), detecting the individual proteins in the single transfections and hAC3-HA in the double transfection. Source data

To investigate whether the expression of a truncated AC3 isoform is conserved in humans, we bioinformatically analysed the human *ADCY3* locus and identified a shorter *ADCY3-AT* mRNA isoform also in humans (*hAC3-AT*; Fig. 6a). To test if *ADCY3-AT* expression is also induced after cold-mimicking stimulation, we isolated the SVF from deep-neck biopsy samples of humans, differentiated these into human WAs and BAs, performed qPCR for full-length and truncated *ADCY3* transcripts after NE stimulation, and observed NE-dependent induction of *ADCY3-AT* expression in human 1°BAs (Fig. 6b). To investigate whether the molecular mechanism underlying AC3-AT-dependent control of cAMP synthesis was conserved in humans, we expressed hAC3 and hAC3-AT in human (HEK293) cells. In line with our findings from mouse, hAC3-AT and hAC3 interact (Fig. 6c,d) and hAC3-AT is sufficient to sequester hAC3 in the ER, as shown by extracellular biotinylation (Fig. 6e,f) and EndoH treatment assays (Fig. 6g). Thus, the molecular mechanism of AC3 ER retention by the NE-inducible AC3-AT is conserved in human BAs.

## Conserved and cold-inducible PPARGC1A -AT drives *Adcy3-at* expression in BAs

**Fig. 7:**
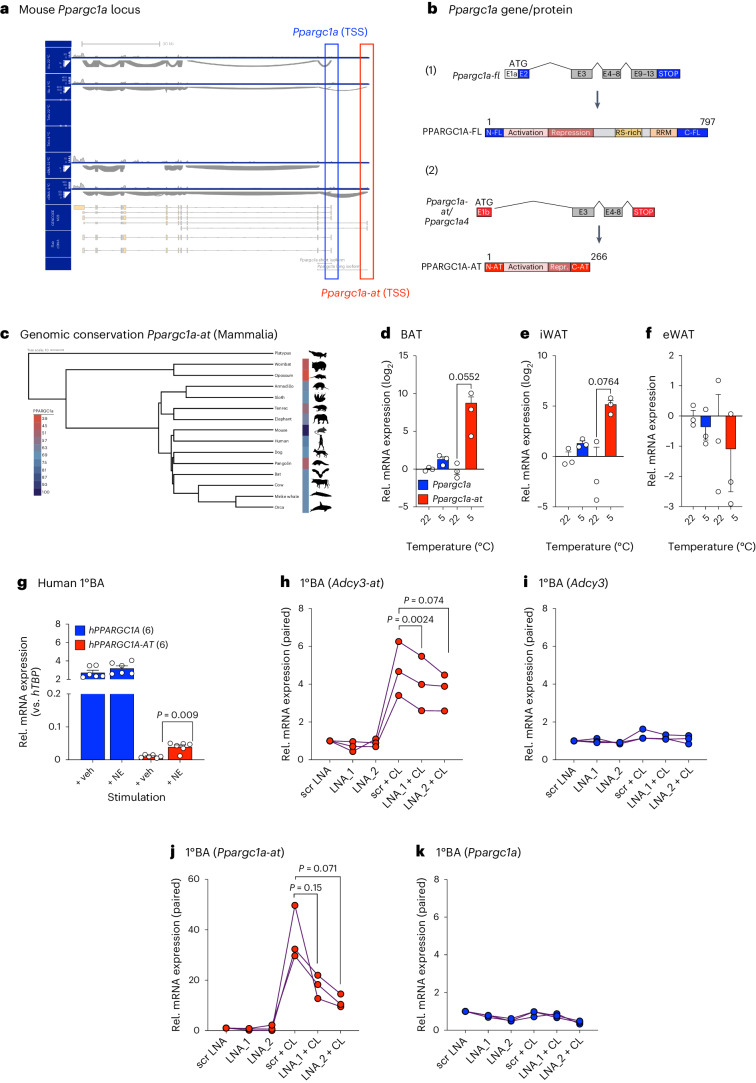
Conserved and cold-inducible PPARGC1A-AT drives *Adcy3-at* expression in BAs. **a**, Sashimi plots visualizing splicing junctions from aligned RNA-seq data in BAT in *Ppargc1* from 20-week-old male C57BL/6N mice at 22 °C or after 24 h of 5 °C CE; *n* = 3–5. Illu, Illumina short-read RNA-seq; Telo, TeloPrime full-length cDNA-seq; cDNA, direct cDNA-seq. Reads were aligned against GENCODE M29 annotation and transcript reassembly using Illumina short-read and full-length RNA-seq using FLAIR^93^. **b**, Schematic of the canonical *Ppargc1a* transcript and PPARGC1A protein structure (1) and *Ppargc1a-at* mRNA and PPARGC1A-AT protein structure (2). Created with BioRender.com. **c**, Genomic conservation of *Ppargc1a-at* among representative mammals. Colour gradient indicates the percentage of nucleotide identity of exon 1b relative to mouse. Phylogeny based on ref. ^89^. **d**–**f**, Relative expression of *Ppargc1a* (blue) and *Ppargc1a-at* (red) as determined by qPCR analysis of primary adipocytes derived from SVF cells from BAT (**d**), iWAT (**e**) and eWAT (**f**) depots. Replicates represent primary adipocytes isolated from individual mice (*n* = 3). Bar graphs represent the mean + s.e.m. with all data points plotted. To test for statistical significance, non-parametric (ranked) Kruskal–Wallis one-way ANOVA tests with Dunn’s correction for multiple testing were performed. *P* values are indicated. **g**, Expression of human *PPARGC1A-AT* in human 1°BAs derived from SVF precursors and stimulated with 1 µM NE for 16 h (*n* = 6 per condition), as described previously^89^. An unpaired, two-tailed and non-parametric Mann–Whitney test was performed to assess statistical significance. Data are shown as a percentage as the mean + s.e.m. with all data points plotted. The *P* value is indicated. **h**–**k**, Expression of *Adcy3-at* (**h**), *Adcy3* (**i**), *Ppargc1a-at* (**j**) and *Pparg1a* (**k**) in mouse 1°BAs after transfection with 25 nM scrambled (scr) LNA inhibitors targeting both *Ppargc1a* (LNA_2) isoforms or exclusively *Ppargc1a-at* (LNA_1) and stimulated with 10 µM CL316,243 for 6 h. Data represent three to four independent experiments, each performed in three technical replicates. Paired samples are represented by individual lines and scr LNA set to unity. Line graphs represent the mean + s.e.m. with all data points plotted. Paired, two-tailed Student’s *t*-tests were performed to assess statistical significance. *P* values are indicated. Source data

PPARGC1A represents a central transcriptional co-regulator of mitochondrial biogenesis^30,56^ and is imperative for BA differentiation and function^57^. Alternative splicing-mediated removal of exons 6–7 of *Ppargc1a* was shown to result in C-terminally truncated NT-PGC1α^58–60^, and NT-PGC1α loss protects against obesity^61^, suggesting negative roles for truncated PPARGC1A protein isoforms in BA differentiation and/or function. Beyond NT-PGC1α, alternative promoter usage and alternative splicing events can give rise to >10 distinct PPARGC1A proteoforms, where each isoforms coordinates specific tissue-dependent and state-dependent adaptive processes^30,62^. When combining Illumina and Nanopore RNA-seq data, we observed another, cold-induced alternative promoter, and the generation of a novel TSS 5′ of canonical, full-length *Ppargc1a* TSS (that is, the *Pgc1alpha1* isoforms reported previously^62^). This gave rise to a 5′-terminally truncated *Ppargc1a* mRNA isoform, which we, analogous to *Adcy3-at*, termed *Ppargc1a-at* (Fig. 7a). *Ppargc1a-at* encoded a C-terminally truncated PPARGC1A-AT proteoform (Fig. 7b) that represented a murine homologue to the *Ppargc1a4* isoform reported in human skeletal muscle^29^. PPARGC1A-AT exon 1 was broadly conserved across eutherian species (Fig. 7c). As PPARGC1A, together with its cognate transcription factors, controls adaptive gene expression programmes^63^, we postulated that PPARGC1A-AT could be implicated in cold-adaptive BA responses, also involving *Adcy3-at* transcriptional activation. Akin to *Adcy3-at*, *Ppargc1a-at* was induced in thermogenic adipose depots during cold, whereas canonical *Ppargc1a* remained unchanged in all depots (Fig. 7d–f). Concomitantly, we observed NE-evoked induction of *PPARGC1A-AT* expression in human BAs (Fig. 7g). To interrogate the functional relationship between PPARGC1A-AT and *Adcy3-at* expression, we designed two independent locked nucleic acid (LNA) inhibitors to silence either (i) *Ppargc1a* and *Ppargc1a-at* in combination (LNA_2) or (ii) *Ppargc1a-at* alone in 1°BAs (LNA_1). Notably, silencing *Ppargc1a/Ppargc1a-at* did not affect *Adcy3*, but prevented CL316,243-mediated *Adcy3-at* induction (Fig. 7h,i), illustrating that PPARGC1A, likely by virtue of truncated PPARGC1A-AT, is implicated in *Adcy3-at* transcriptional regulation.

**Extended Data Fig. 10:**
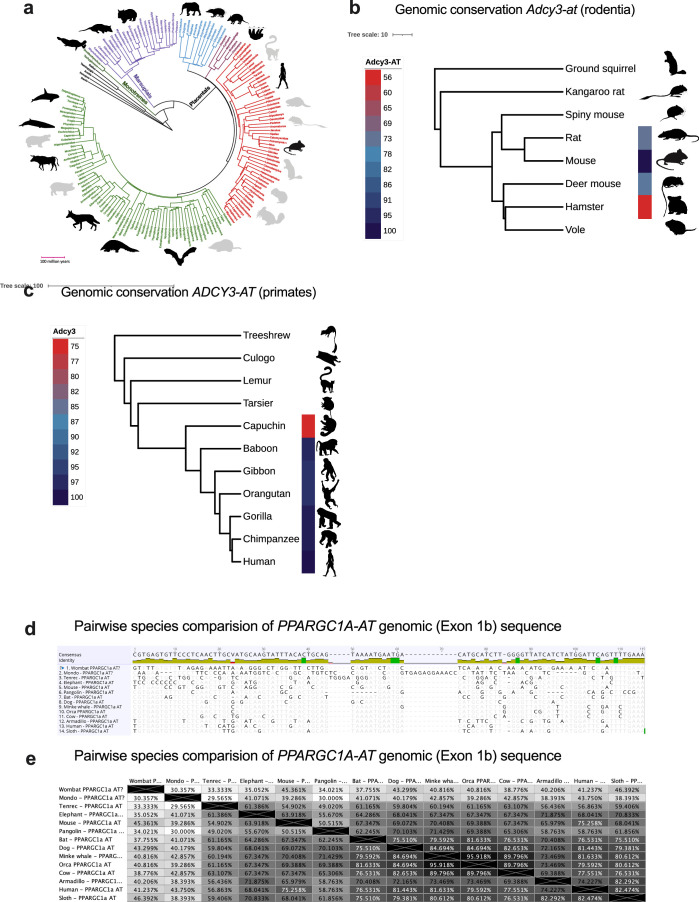
Conservation of Adcy3-at and Ppargc1a-at. Evolutionary conservation of Adcy3-at and Ppargc1a-at isoforms. **a**, Mammalian phylogeny from Meredith et al.^89^ modified in iTOL^88^, with silhouettes in black indicating representative species that were selected for comparative analyses of PPARGC1A-AT. **b**, Genomic conservation among representative rodent species (rodentia) of Adcy3-at identified in the mouse. Color gradient indicates percent nucleotide identity of the Adcy3-at relative to the mouse. Phylogeny based on Swanson et al.^91^. **c**, Genomic conservation among representative primates (primates) of Adcy3-at identified in human. Color gradient indicates percent nucleotide identity of Adcy3-at relative to the human. Phylogeny based on Perelman et al.^90^. **d**, Nucleotide sequence alignment of Ppargc1a-at (Exon 1b) from various mammalian species. **e**, Percent identity matrix of Ppargc1a-at among 14 representative mammals (exon 1b). Reproduced from ref. ^88^ under a Creative Commons license CC BY 4.0.

Finally, we investigated the broader evolutionary context of *ADCY3-AT* and *PPARGC1A-AT* by DNA sequence comparison of *Adcy3-at* and *Ppargc1a-at* genomic sequences across a panel of thermogenic and non-thermogenic species (Extended Data Fig. 10a): Intriguingly, mouse *Acy3-at* was conserved only within selected rodent species (Extended Data Fig. 10b) and thus likely arose independently from human *ADCY3-AT*, which itself arose among primates (Extended Data Fig. 10c). Despite this convergent evolution, rodent and primate *Adcy3-at* transcripts encoded sequence-related and structurally related proteins. In contrast to *ADCY3-AT*, exon 1 of *PPARGC1A-AT* was more broadly conserved across eutherian species (Fig. 7c and Extended Data Fig. 10d,e). Thus, CE uses an evolutionarily ancient signal transduction axis that couples truncated PPARGC1A-AT proteins to cold-induced, rheostatic transcriptional responses in activated BAs that, among others, involves AC3-AT.

## Discussion

Since the rediscovery of active and recruitable BAT in humans^3–5^, enhancing brown fat activity has been recognized as an innovative therapeutic approach against obesity and obesity-related health decline. Understanding BAT activity and, ultimately, increasing energy expenditure via BAT activation thus holds promise for promoting metabolic health. While the transcriptional circuitry and hormonal cues driving thermogenic activation of BAT is well understood, we lack detailed understanding of how BAT activation is kept in balance to avoid negative consequences of prolonged energy dissipation. Here, we have delineated a new, evolutionarily conserved molecular mechanism that functions as a rheostat for maintaining (and limiting) BAT function. This mechanism involves the expression of an N-terminally truncated AC3-AT protein isoform, which fine-tunes cAMP synthesis during periods of chronic CE and, thereby, maintains BAT function during alterations of energy homeostasis.

Expression of novel AC isoforms has also been demonstrated in other cell types, that is, in vascular smooth muscle cells (VSMCs): A crucial change in VSMC function is the trans-differentiation from a contractile/quiescent to a more secretory/proliferative phenotype. This process underlies atherogenesis and vascular remodelling^64^ in response to pro-atherogenic cytokines such as interleukin-1β and relies on the de novo expression of AC8 in VSMCs^65,66^. However, an increase in intracellular cAMP levels is supposed to inhibit the secretory/proliferative VSMC phenotype^67,68^. Strikingly, the AC8 isoforms expressed in VSMCs after interleukin-1β stimulation are splice variants, resulting in shorter AC8 proteins that are also catalytically inactive. N-terminally truncated AC8 proteins act in a dominant-negative manner on other AC isoforms by forming heterodimers, retaining them in the ER and reducing intracellular cAMP levels^69,70^. This is in line with our results, mirroring our observation of a stimulus-dependent de novo expression of truncated mAC3-AT in BAT. Thus, we here demonstrate that balancing cAMP levels by expressing truncated AC isoforms is a more broadly applicable mechanism that occurs not only in VSMCs but also in thermogenic fat and across different AC isoforms. Whether the regulation of de novo AC expression is regulated by common signalling pathways, but across these different cell types, is not known. We here identified a H3K4me3-marked promoter in intron 2 of the murine *Adcy3* gene which, selectively induced during cold, resulted in a novel TSS for a 5′-truncated *Adcy3-at* mRNA isoform. The regulation of epigenetic modifications such as H3K4me3 in the context of cAMP signalling is unknown, but it has been shown that expression of a histone demethylase is controlled by the transcription factor CREB (cAMP-response binding protein), which itself is activated by cAMP/PKA-dependent phosphorylation^71^. For instance, the beta-2 adrenergic receptor agonist clenbuterol engages cAMP–PKA–p-CREB signalling, which then drives the expression of Jhdm2a, a histone demethylase via direct binding of p-CREB to the CRE (cAMP-response element) site in the JHDM2a promotor^72^. For AC8, de novo expression seems to result from both transcriptional activation and alternative splicing^69^. Strikingly, the mouse promoter contains a CRE site upstream of the TSS that is required for basal and cAMP-dependent promoter activity^73,74^. Thus, cAMP-dependent control of *Adcy* isoforms, directly or indirectly by epigenetic modifications, might have emerged as a unifying mechanism.

Expression of truncated protein isoforms in metabolically active tissue, like adipose tissue or muscle, is conserved among different proteins that converge in common signalling pathways in a cell-type-specific manner. Here, we demonstrated that CE results in the expression of a truncated PPARGC1A-AT proteoform, which contributes to induction of *Adcy3-at* expression. The stimulus that induces de novo expression of the truncated proteins can, however, be different yet appropriate towards the seminal function of the tissue, that is, CE for the thermogenic BAT and endurance training for truncated PPARGC1A isoforms leading to the expression of truncated PPARGC1A isoforms PGC-1a2, PGC-1a3 or PGC-1a4 (refs. ^29,62,75^). As these stimuli, and the resulting protein expression, are crucial for the function and homeostasis of respective tissues, identifying pharmacological means to target these truncated isoforms is therapeutically relevant. Based on our results from *Adcy3∆AT* mice, pharmacologically inactivating *Adcy3-at* expression or impeding the interaction of AC3-AT with AC3 could pave the way to reactivate BAT function and mitigate obesity and obesity-related metabolic decline.

## Methods

### Animal care and research diets

Experimental animals were kept in individually ventilated cages (IVC type II long) in a specific pathogen-free animal facility with controlled temperature (22–24 °C), light–dark cycle (12 h–12 h) and humidity (50–70%). Care of animals was within institutional and animal care committee guidelines approved by (1) local (Bezirksregierung Köln) or regional (Tierschutzkommission acc. §15 TSchG of Landesamt for Natur, Umwelt und Verbraucherschutz (LANUV) North-Rhine Westphalia, Germany) authorities (internal accession no. 84-02.04.2017.A009) or (2) Ministry of Environment of Denmark (Miljø- og Fødevarestyrelsen; internal accession no. 2018-15-0201-01562). All animals were maintained and regularly backcrossed to a C57BL/6N background and housed in groups of 3–4 animals per cage and had ab libitum access to food and drinking water. All mice were euthanized by cervical dislocation or carbon dioxide asphyxiation. Unless otherwise indicated, animals were allowed ad libitum access to chow diet (Ssniff R/M-H Low-Phytoestrogen, V1554) containing 62 kJ% carbohydrates, 27 kJ% protein and 11 kJ% fat and drinking water. DIO was achieved by feeding a HFD (D12492 (I) mod; Sniff, Research Diets) containing 20 kcal% carbohydrates, 20 kcal% protein and 60 kcal% fat starting at 6–7 weeks of age. Animals were excluded from the experiments when experiencing HFD-induced skin rashes or wounds from fighting.

### Mouse husbandry

Adcy3 floxed mice were kindly provided by Chen and colleagues^24^. Herein, exon 3 of the *Adcy3* gene is flanked by two intronic *LoxP* sites, each 75 bp upstream or downstream of the exon. Deleting exon 3 of *Adcy3* causes a frameshift mutation, resulting in a premature stop codon within the *Adcy3* gene. Adipose tissue-specific deletion of both *Adcy3* and *Adcy3-at* was achieved using recombinase-mediated excision of *LoxP*-flanked (‘floxed’) gene sequences. For this, mice floxed for *Adcy3* (*Adcy3*^*LoxP/LoxP*^) were interbred with mice expressing the Adipoq-cre recombinase under the control of mature adipocyte-specific Adipoq promoter (Adipoq-cre^+/cre^)^37^. Adipoq-Cre mice were obtained from The Jackson Laboratory (Jax stock no. 010803) and backcrossed to C57BL/6N for at least five generations. Resulting *Adcy3*^*LoxP/LoxP*^, Adipoq-cre^+/cre^ mice (*Adcy3-AdcKO*) were compared to *Adcy3*^*LoxP/LoxP*^, Adipoq-cre^+/+^ littermates as controls (*LoxP*).

### Generation of Adcy3∆AT mice

*Adcy3∆AT* knockout ES cells were generated using CRISPR–Cas9 technology using a modified pX330 plasmid vector^76,77^ containing two gRNAs and a GFP-puromycin selection cassette. The plasmid was kindly provided by S. Frank and was constructed at the Max Planck Institute for Molecular Biomedicine (Münster, Germany). The knockout strategy targeted the 978-bp deletion of *Adcy3-at* starting exon (‘exon 2b’) and flanking regions from *Adcy3* genomic DNA. A CRISPR design online tool (http://crispor.gi.ucsc.edu/) was used to generate *Adcy3-at* specific sgRNA sequences. The list of sgRNAs and primers for generation of *Adcy3∆AT* mice are given in Supplementary Table 3. The quality score of the design pipeline represents the reliability of an on-target activity computed as 100%, minus a weighted sum of off-target hit-scores. A chosen gRNA with a high-quality score was cloned into the BbsI-digested pX330 vector, followed by the second gRNA, which was cloned using a SapI-mediated Golden Gate assembly method for the concatemerization of gRNA cassettes.

### Vector generation

For CRISPR–Cas9 construct generation, 1 μg of the pX330 vector was enzymatically digested with FastDigest BbsI (Thermo Fisher Scientific) for 30 min at 37 °C. The digested vector was purified using the MinElute PCR Purification kit (Qiagen) following the manufacturer’s instructions. gRNA was phosphorylated by a T4 polynucleotide kinase according to product instructions (New England Biolabs). For the amplification of phosphorylated sgRNA, it was incubated with 1 μl of forward and reverse primers (100 μM; Supplementary Table 3) for 30 min at 37 °C. Annealing was performed at 95 °C for 5 min and subsequently the reaction was cooled to 25 °C at a cooling rate of 5 °C per minute. Then, 1 μl of the oligonucleotide reaction was ligated with 50 ng of digested pX330 vector and was assessed using the Quick Ligase kit (New England Biolabs) for 20 min at RT according to the manufacturer’s instructions. Plasmid Safe DNase kit (EpiCentre) was used to degrade any unspecific recombination products for 30 min at 37 °C, following the instructions of the kit.

### Culturing mouse EFs and ES cells and ES cell transfection

Mouse embryonic fibroblasts (EFs) were used as feeder cells for mouse ES cells. EFs were passaged before reaching full confluence and EF-cell medium was changed to fresh medium every 2 days. To obtain mitotically inactive fibroblasts, cell growth was stopped by adding 10 μg ml^−1^ mitomycin C (MMC) after three generations (EF3), and the reaction was incubated for another 2–4 h. MMC was removed, and cells were rinsed with 1× PBS. The cells were afterwards stored by freezing or directly used for seeding. To seed MMC-treated EF3 cells, tissue culture dishes were gelatinized with 0.2% gelatine in advance to enhance the attachment of MMC-treated cells to the dish surface. ES cells were cultivated on top of MMC-treated EF3 cells and ES cell culture medium was exchanged every day. ES cells were passaged to new culture plates or were frozen for long-term use and to maintain pluripotency.

10 × 10^7^ ES cells were mixed with 40 μg of the CRISPR construct dissolved in PBS, resuspended in 400 µl transfection buffer (RPMI without Phenol Red, Gibco) and mixed with the plasmid construct DNA. The total volume was adjusted to 800 μl. Transfection was achieved by electroporation at 450 μFD and 240 V at RT (Gene Pulser Xcell, Bio-Rad Laboratories) in a 4-mm electroporation cuvette. The time constant was set to around 7–10 s. After 5 min of incubation, cells were resuspended in ES cell medium, diluted at a 1:20,000 ratio and plated on a gelatinized 10-cm culture dish with previously prepared MMC-treated EF3 cells. Following 2 days of incubation in ES cell medium, single clones were transferred to three 96-well plates and selected via colony PCR. In addition to PCR confirmation, Southern blot analysis was used to confirm the deletion of *Adcy3-AT* as explained below. Validated colonies were transferred in 40 μl PBS to a 96-well (round-bottom) plate containing 25 μl trypsin. Trypsinization took place for 5 min at 37 °C and the reaction was stopped by adding 100 μl ES cell medium. Cell suspension for each colony was added up to 15 ml with ES cell medium and seeded in one separate 10-cm dish per colony. To achieve higher homogeneity among targeted ES cell populations, positive colonies were again seeded out after expansion in three 96-well plates each and selection by PCR and Southern blot was repeated. Positive colonies were trypsinized and seeded on 10-cm dishes for expansion as before.

### Generation of *Adcy3∆AT* chimeric mice from genetically modified ES cell clones and genotyping

Positive *Adcy3∆AT* clones were injected into donor blastocysts, which was performed by Taconic Biosciences. Injected blastocysts were implanted into pseudo-pregnant foster mice and resulting progenies (generation F1) partially developed from cells derived from *Adcy3∆AT* ES cells. Male chimeric F1 mice were backcrossed to C57BL/6N mice to produce the F2 generation. Germline transmission was confirmed by PCR and Sanger sequencing of DNA extracted from the tails of F2 mice. Genotyping protocols for the *Adcy3∆AT* transgenic allele were conducted using the following primers, which were also used for genotyping from mouse tail DNA:

*Adcy3∆AT*_E2B_F_1: 5′-GGGACTGAGGGAGCCTAAGA-3′,

*Adcy3∆AT*_E2B_R: 5′-GGCCAGGTTACATGAGGACA-3′,

*Adcy3∆AT*_E2BF_2_R2: 5-’-GGAGAGCTTCGAGTGTGTCAAG-3′.

### Southern blot of *Adcy3∆AT* ES cells

For confirmation of *Adcy3-at* deletion in ES cells, genomic DNA (5–10 μg) derived from transfected ES cells was digested by HindIII-HF or BamHI-HF (NEB) at a high concentration. In all Southern blot analyses, wild-type Bruce4 (Br4) ES cell DNA was used as the negative control. Overnight digested DNA was loaded with loading dye onto a 0.8% agarose gel and separated at 30 V for at least 16 h. The DNA was consequently de-purinated within the gel by incubating the gel in 0.25 M HCl for 20 min under continuous shaking. DNA was then transferred into a charged Amersham Hybond-XL nylon membrane by alkaline capillary transfer using a sodium hydroxide solution (0.4 M NaOH) and the membrane was incubated in a 2× SSC buffer for 20 min and finally dried at 80 °C for 45 min to fix the DNA on the membrane. To probe for the modified *Adcy3∆AT* allele, a 394-bp probe was amplified from Bruce4 ES cell genomic DNA with primers 5′-ATCCTGTGCTGACATGGGTG-3′ and 5′-AATTCCCCACTGACCAACGG-3′ using a high-fidelity PCR Master Kit. The extracted probes were radioactively labelled with 2.5 μl (alpha-32P) dCTP (Amersham) according to the instructions of the kit (Ladderman Labeling Kit, Takara). The probe was mixed with 100 μl TE buffer (10 mM, pH 8.5) and purified via spin-column chromatography using Illustra MicroSpin S-200 HR columns. The dried membrane was incubated in a 2× SSC buffer to prepare for pre-hybridization. Pre-hybridization was performed in pre- hybridization buffer (1 M sodium chloride, 50 mM Tris-CL pH 7.5, 10% dextran sulfate, 1% SDS, 250 μg ml^−1^ salmon sperm DNA) at 65 °C for at least 4 h. After pre-hybridization, radioactively labelled probes were added to the membrane and the reaction was incubated overnight at 65 °C. After several washing steps, the membrane was placed in direct contact with Kodak MS hypersensitive films for 4–24 h to detect the radioactive signal. Expected product sizes were 4.75 kb for *Adcy3* wild-type and 3.75 kb for *Adcy3∆AT* constructs.

### Plasmid vector cloning

Overexpression constructs (mAC3-AT, 3,201 bp and mAC3, 3,792 bp) were PCR amplified from synthetic oligonucleotides by Thermo Fisher Scientific. The DNA fragment was inserted into pcDNA3.1(+) vector backbones using NheI/XhoI restriction enzymes. For mAC3-AT, the first ATG in-frame within exon 2 was used as the start codon. Sequences encoding Kozak sequences (GCCACC) and a 295-bp artificial intron sequence were included upstream of ATG. To generate pc3.1(+)-mAC3-AT-FLAG, the FLAG tag was added by PCR and the coding sequence cloned into the vector backbone using EcoRI/XhoI. Additional details on cloning procedures are available upon reasonable request. Overexpression constructs (hAC3-HA, 3,474 bp and hAC3-AT-FLAG, 2,748 bp) were PCR amplified from synthetic oligonucleotides by Gene Link. The DNA fragment was inserted into the pRP vector backbone^78^ using AscI/XhoI restriction enzymes.

### Illumina and Nanopore RNA-seq and data analysis

Paired-end libraries were constructed using the NEBNext Ultra II RNA Library Prep Kit (New England Biolabs) following the manufacturer’s protocol and sequenced on a NovaSeq 6000 (Illumina) in 2 × 50-bp paired-end reads. Transcriptional expression was quantified using Salmon^79^ by mapping RNA-seq reads to the ENSEMBL m38 (mm10) transcriptome. *Adcy3-at* transcript identified in the de novo ONT/Illumina transcriptome assembly (see below), was added to the ENESEML m38 transcriptome and counted as an independent gene. Transcript counts were imported and summarized to ENSEMBL gene IDs using tximport^80^ and differential expression analyses were conducted with edgeR^81^ using glmQLFTest. KEGG pathway enrichment analyses were conducted using the clusterProfiler package for R^82^.

### Nanopore RNA-seq and data processing

RNA was isolated by phenol-chloroform extraction and alcohol precipitation followed by two consecutive rounds of poly-A selection using oligo(dT) beads (GenElute mRNA Miniprep Kit, Sigma, MRN10) following the manufacturer’s recommendations. Subsequently, RNA was alcohol precipitated using sodium acetate and glycogen following the protocol from the Ribo-Zero rRNA Removal Kit (Illumina). Nanopore sequencing libraries were prepared using the TeloPrime Full-Length cDNA Amplification Kit (Lexogen), the SQK-DCS109 direct cDNA sequencing kit (ONT) and the SQK-RNA002 direct RNA-seq kit (ONT) according to the manufacturer’s protocols. Sequencing was performed using FLO-MIN106 R9 flow cells on the GridION platform (ONT). Reads were mapped against the murine genome (GRCm38.p6) using minimap2 (ref. ^83^) and transcriptomes reassembled using FLAIR75. Tracks were visualized using gviz^84^.

### snRNA-seq data processing

Preprocessed data from snRNA-seq from 10x sequencing of 1°BAs, isolated from male C57BL/6N mice housed at RT, CE and/or TN were adopted from Sun34 (ArrayExpress using accession code E-MTAB-8562). Data were normalized (SCTransform), analysed and plotted using Seurat 4.0.1 package for R^79–82^.

### ChIP–seq and ChIP–qPCR

Before ChIP–seq, BAT was dissociated using a gentleMACS Dissociator (Miltenyi Biotec). The cell suspension was crosslinked with 1% formaldehyde for 10 min at RT, and the reaction was quenched with 0.125 M glycine for 5–10 min at RT. Cells were washed 2× with cold PBS and phenylmethyl sulfonyl fluoride and snap frozen in liquid nitrogen before storing at −80 °C. For histone posttranslational modification sequencing, BAT of two mice was used and steps were performed on ice. First, frozen pellets were thawed on ice for 30–60 min. Pellets were resuspended in 5 ml LB1 (50 mM Hepes, 140 mM NaCl, 1 mM EDTA, 10% glycerol, 0.5% NP-40, 0.25% Triton X-100) by pipetting and then rotated vertically at 4 °C for 10 min. Pellets were centrifuged for 5 min at 1,350*g* at 4 °C (GH-3.8 rotor = 2,400 rpm) and supernatant was aspirated. Pellets were resuspended in 5 ml LB2 (10 mM Tris, 200 mM NaCl, 1 mM EDTA, 0.5 mM EGTA) and incubated at vertical rotation and at RT for 10 min. Samples were centrifuged for 5 min at 1,350*g* at 4 °C and supernatant was carefully aspirated. Then, samples were resuspended in 3 ml LB3 (10 mM Tris, 100 mM NaCl, 1 mM EDTA, 0.5 mM EGTA, 0.1% sodium deoxycholate, 0.5% *N*-lauroylsarcosine) and were separated into 2 × 1.5 ml in 15-ml polypropylene tubes, in which they were sonicated with the following settings by Bioruptor Plus sonication: Power = high, ‘on’ interval = 30 s, ‘off’ interval = 45 s and total time = 10 min (18 cycles of ‘on’/’off’). Sonicated samples were transferred to a 1.5-ml microcentrifuge tube and were centrifuged for 10 min at 16,000*g* at 4 °C to pellet cellular debris. Around 10% of the sample solution was stored to be used as input control, while the rest was used for ChIP. To capture different H3K4me3 modifications, 3 µl of anti-H3K4me3 antibodies (Active Motif, 39159) was added to the sonicated ChIP reaction and rotated vertically at 4 °C overnight. The next day, 100 μl Dynabeads (protein A or protein G) for each ChIP sample was prepared according to the manufacturer’s instructions, mixed with 1 ml of antibody-bound chromatin, and rotated vertically at 4 °C for at least 2–4 h. Bound beads were washed at least five times in 1 ml cold RIPA and once in 1 ml cold TE buffer containing 50 mM sodium chloride. Samples were eluted for 15 min with elution buffer at 65 °C and continuously shaken at 700 rpm. Beads were separated using a magnet, and 200 μl supernatant was transferred to fresh microcentrifuge tubes. Previously stored input samples were thawed and mixed with 300 μl elution buffer. ChIP/input samples were incubated at 65 °C in a water bath overnight to reverse the crosslinking reaction. On the third day, one volume of TE buffer was added at RT to dilute SDS in both ChIP and input samples. For digestion of RNA and protein contamination, RNase A was added to the samples and incubated in a 37 °C water bath for 2 h; then proteinase K was added to a 0.2 mg ml^−1^ final concentration and incubated in a 55 °C water bath for 2 h. Finally, DNA was extracted using a standard phenol-chloroform extraction method at RT, and DNA concentrations were measured using a NanoDrop ND-1000 spectrophotometer or Qubit dsDNA HS Assay Kit and stored at −80 °C until sequencing or ChIP–qPCR.

H3K4me3-immunoprecipitated genomic DNA was diluted at a 1:100 ratio. ChIP–qPCR was performed with a LightCycler 480II machine (Roche) using technical duplicates, and ChIP–qPCR signals were calculated as a percentage of input. Standard deviations were calculated from technical duplicate reactions and represented as error bars. qPCR primer sequences were:

*Adcy3-fl* TSS_F: 5′-GATGGACTTCCACGAGGCTG-3′,

*Adcy3-fl* TSS_R: 5′-TACTTCCTTTCCCCCACCCA-3′,

*Adcy3-at* TSS_F: 5′-GGAGAGCTTCGAGTGTGTCAAG-3′,

*Adcy3-at* TSS_R: 5′-GTCTCACCTTAAGGCTCCTCCT-3′,

*Adcy3* exon 3_F: 5′-CTGTGCCAGATTGTCTCCGT-3′,

Adcy3 exon 3_R: 5′-GTCATGGACTTGGGCTTCCA-3′.

### Mouse tissue RNA isolation

RNA from indicated tissues and 1°BAs and 1°iWAs was isolated using TRIzol according to the manufacturer’s protocols for total RNA isolation.

### CHO K1 cell culture

CHO K1 WT cells (American Type Culture Collection, CCL-61) were maintained in F-12 Nut Mix + GlutaMAX (31765-027, Thermo Fisher) at a subconfluent level and passaged every 4 days. Cells stably expressing ADCY3-FL-HA were kept in medium containing 0.8 mg ml^−1^ Geneticin (G418 Sulfate, Gibco).

### HEK293T cell culture

HEK293T (American Type Culture Collection, CRL-3216) cells were maintained in DMEM + GlutaMAX (3196602, Thermo Fisher) supplemented with 10% FCS (Biochrome) at 37 °C and 5% CO_2_ atmosphere.

### Isolation of depot-specific SVF-derived primary adipocytes

Inguinal white and intrascapular BAT from 6- to 8-week-old male mice was dissected after carbon dioxide asphyxiation, minced and digested with collagenase II (2 mg ml^−1^), DNAse I (15 kU ml^−1^), Dispase II (for BAT: 1.5 mg ml^−1^) and 3.4% BSA-supplemented serum-free SVF culture media DMEM/F-12 pre-supplemented with 1% penicillin–streptomycin, 0.1% biotin and 0.1% pantothenic acid. Minced tissues were incubated in a 37 °C shaker at 120 rpm per minute for 45 min with pipetting every 15 min until the minced tissue particles were completely dissolved. SVF fraction cells were enriched in the pellet of the tubes by sequential filtering and washing steps with 250-µm and 70-µm sterile cell strainers in 10% FBS-supplemented SVF culture media. Enriched SVF cells were seeded in six-well plates (one well per mouse) with 20% FBS-supplemented SVF culture media and incubated in a cell culture incubator for the required amount of time to enable expansion of the culture.

### Induction of SVF adipogenesis

iWAT and BAT depot-specific SVF at 80% confluency were induced for adipogenic differentiation with induction cocktail (1 M rosiglitazone, 850 nM insulin, 1 M dexamethasone, 250 M IBMX and, for only BAT, 125 M indomethacin and 1 nM triiodothyronine (T3)) in 10% FBS-supplemented SVF culture media. After incubation for 2 days with induction media, culture media were replenished with the differentiation media (1 M rosiglitazone, 850 nM insulin and for only BAT with 1 nM T3 in 10% FBS-supplemented culture media) on days 2, 4 and 6. Cells were treated with compounds (10 µM CL316,243; 2 M forskolin) on day 8 for 6 h and harvested the same day after compound stimulations.

### LNA-mediated gene knockdown in primary adipocytes

For transfection of LNA GapmeRs in 1°BAs and 1°iWAs, 80,000 cells were seeded per well in six-well plates and grown to confluence in growth medium. Immediately before transfection, medium was changed to fresh differentiation medium without antibiotics. In the meantime, antisense LNA GapmeRs (Exiqon A/S) were diluted to the concentration of 100 nM in OptiMEM and transfected using Lipofectamine 2000 according to the manufacturer’s instructions. LNA sequences are provided in Supplementary Table 5.

### AAV-mediated cre transduction in primary adipocytes

On day 4 of iWA and BA differentiation, committed adipocytes were trypsinized, counted and seeded into multiwell plates with the experimental setup (180.000 cells per cm^2^ growth area) with the differentiation media containing AAV viruses. Primary adipocytes were transduced with AAV8-CMV-Cre (pENN.AAV.CMVs.Pl.Cre.rBG, Addgene, 105537-AAV8-Cre) and AAV8-CMV-eGFP (pAAV.CMV.PI.EGFP.WPRE.bGH, Addgene, 105530-AAV8 eGFP) viruses at a multiplicity of infection of 100,000. Virus containing media were replenished on day 6 with fresh differentiation media and cells were treated with compounds (10 µM CL316,243) on day 7 for 6 h and harvested the same day after compound stimulations.

### Determination of oxygen consumption rates and measurement of glycolytic activity

*Adcy3-at*-deficient SVFs from indicated adipose depots of transgenic mice were seeded into Agilent Seahorse XFe96 Bioanalyzer microplates. Per well, 50,000 cells were seeded and incubated in DMEM/Ham’s F-12 medium plus 10% FCS, 1% penicillin–streptomycin, 0.1% biotin and 0.1% pantothenic acid (growth medium) at 37 °C and 5% CO_2_ at a standard incubator until confluency is reached. To induce commitment of SVFs into mature adipocytes within Xfe96 microplates, freshly prepared 0.05% insulin, 0.005% dexamethasone, 0.001% rosiglitazone and 0.05% IBMX (1°iWA) or 0.1% indomethacin, 0.001% T3 (1°BAs) in growth medium (induction medium) were added. After 48 h of induction, differentiation was initiated using freshly prepared 0.001% rosiglitazone (1°iWAs) or 0.001% T3 (1°BAs) in growth medium (differentiation medium). Differentiation was achieved after 3–4 days of incubation in the differentiation medium. For each seahorse plate, the corresponding calibration plate was prepared 24 h before experiments using 200 µl XF Seahorse Calibrant Agilent per well. The plate was incubated for 24 h in a non-CO_2_ incubator at 37 °C and the instrument set to 37 °C 24 h before the experiment. One hour before measurements, plates were washed with 1× PBS and the medium changed according to the corresponding experiment analyte kits (MitoStressKit or GlycoStressKit, provided by the manufacturer). Before measurement, calibration was started using calibration plates, measuring O_2_ and pH LED value/emission/initial reference delta for each well. After calibration, cartridges were kept within the machine and measurement of adipocyte-containing microplates commenced. Measurement parameters were: Mix 3 min, wait 0 min, measure 3 min with each reagent’s effect assessed within three (MitoStressKit) or four (GlycoStressKit) consecutive measurement cycles with a total duration of 18 min or 24 min per reagent injection. All measurements started with measuring basal values, followed by injection of oligomycin, FCCP and rotenone plus antimycin A (MitoStressKit) or glucose, oligomycin and 2-deoxy-glucose (GlycoStressKit). For MitoStressKit, corresponding media were prepared before the experiment and consisted of Basal Seahorse Medium supplemented with 25 mM glucose, 1 mM glutamine, 2 mM sodium pyruvate, set to a pH of 7.4 and filtered sterile. Around 25 ml of MitoStress Medium per plate was needed and Seahorse cell plates were changed to 180 µl MitoStress medium 1 h before calibration in a non-CO_2_ incubator at 37 °C. The calibration plate possessed a cartridge with four pockets per well. Before the measurement, pocket A was filled with 20 µl 10 µM oligomycin, pocket B with 22 µl 10 µM FCCP and pocket C with 25 µl 5 µM antimycin A and rotenone. For GlycoStressKit, the cell plate was washed with 1× PBS, and the medium was changed to filtered 180 µl GlycoStressKit medium. GlycoStressKit medium consisted of Basal Seahorse Medium supplemented with 1 mM glutamine and 2 mM sodium pyruvate, set to a pH of 7.4 and stored for 1 h in a non-CO_2_ incubator at 37 °C. The calibration plate possessed a cartridge with four pockets per well. Shortly before the measurement, pocket A was filled with 20 µl 10 mM glucose, pocket B with 22 µl 10 µM oligomycin and pocket C with 25 µl 50 mM 2-deoxy-glucose.

### Food intake determination

Food intake was monitored after 7 weeks of diet intervention. Mouse cages (*n* = 4–5 male mice) were equipped with food hoppers, and food was weighed before being provided to the cages and daily during the span of 4 days. Mice had ad libitum access to food and water. Food intake was calculated by subtracting the amount of food available on the respective day to the previous measurement and divided by the number of mice present in the cage.

### Intraperitoneal glucose tolerance test and insulin tolerance test

Glucose tolerance tests were carried out at 00:00 after a 6-h fast starting in the morning. After determining basal blood glucose levels (0 min), animals received an intraperitoneal bolus of 2 g glucose per kilogram of BW (20% glucose, Delta select). Blood glucose levels were determined 15, 30, 60 and 120 min after injection using an automatic glucose monitor (Contour, Bayer Diabetes Care). Insulin tolerance tests were carried out after a 4-h fast of mice at 00:00 in fresh cages with bedding, free access to drinking water but no food. After determining basal blood glucose levels (0 min), each animal received 0.75 IU per kg of BW of insulin (Actrapid; Novo Nordisk). Blood glucose levels were recorded after 15, 30, 60 and 120 min. Those animals that showed no increase/decrease of blood glucose levels after intraperitoneal injection of glucose or insulin, assuming injection outside the peritoneal cavity as required for the assay, were excluded from analysis.

### Indirect calorimetry (PhenoMaster)

Indirect calorimetry (PhenoMaster, TSE Systems) was used to evaluate energy expenditure, locomotor activity, RERs as well as food and water intake in (1) the animal facility of Max Planck Institute for Metabolism Research, (2) the Biomedical Laboratories (University Hospital Odense) or (3) in the iFET animal facility of the BMZ-II, Medical Faculty, University of Bonn. Mice were allowed to acclimatize to single housing conditions in metabolic cages for 2 to 4 days. Food and water were provided ad libitum during acclimatization and throughout all experimental measurements. All parameters of indirect calorimetry were measured for a period of 1–10 days depending on the duration of CE. Oxygen consumption and carbon dioxide production were measured in units of volume every 10 min for indicated amounts of days (including measurements for both cold and warm ambient temperatures) to determine the respiratory quotient (RQ = VCO_2_/VO_2_ 2/VO_2_))) × 4.1868). Locomotor activity was measured by a multidimensional infra-red light beam system. Integrated scales assessed water and food intake at 2-min intervals and automatically calculated cumulative water and food intake. During measurements in the PhenoMaster System, mice were initially kept at a constant temperature of 23 °C for 72 h, followed by 4 °C for 1–6 days depending on the experimental setup.

### BW and body composition monitoring using NMR

Mouse BW was measured weekly. Body composition was assessed on the last intervention day by NMR using Bruker MiniSpec LF50 (Bruker).

### Implant of electronic transponders

Electronic ID transponders (2175429, Bio Medic Data Systems) were implanted subdermally, in the mouse’s interscapular depot. Mice were anaesthetized by continuous isoflurane exposure: using a calibrated anaesthetic delivery machine, mice were induced into anaesthesia at a dose of 4% isoflurane, then maintained at a surgical plane by continuous inhalation of 2% isoflurane. Once anaesthesia was established, electronic ID transponders were injected into the interscapular depot, and mice were transferred individually to cages for recovery.

### Cold exposure

Mice were housed individually and with reduced cage bedding and kept at 6 °C for 4 h. Interscapular temperature was measured from the injected transponders every 30 min using a handheld reader XPT/IPTT (110090, Bio Medic Data Systems).

### Tissue fixation and histology

iWAT, gWAT and iBAT were fixed in 4% paraformaldehyde for 24 h before further processing using the automated Epredia Excelsior AS Tissue Processor (Thermo Fisher Scientific). Tissues were dehydrated in increasing ethanol concentrations during six dehydration steps (70–100% at 30 °C for 1 h each, UKB Pharmacy), followed by three steps in the clearing agent xylene (30 °C, 1 h each, AppliChem) to clear out the ethanol before incubation of samples in molten paraffin wax for three times (62 °C, 80 min each, Labomedic) to replace the xylene by infiltrating the samples. In the following, infiltrated samples were casted into moulds together with liquid paraffin (65 °C) and cooled to a solid block with embedded tissues (Leica EG1150 H Paraffin Embedding Station and Leica EG1150 C Cold Plate). After embedding, adipose tissue samples were sliced into 5-µm sections using a Thermo Scientific HM 355S Automatic Microtome and mounted on Surgipath X-tra Microscope Slides (Leica Biosystems). Mounted sections were stained for histological analysis with Mayer’s hemalum solution (Sigma-Aldrich) and eosin Y solution (1% in water, Roth) using Leica ST5020 Multistainer combined with Leica CV5030 fully Automated Glass Coverslipper. Afterwards, deparaffinization of paraffin-embedded slices was performed in two heating steps (60 °C; 6 min each) to melt the wax, followed by incubation in xylene (three times, 1 min each). Rehydration of samples took place in a graded ethanol series (100–70% ethanol, 80 s each) before rinsing the samples in sterile distilled water (dH_2_O, 80 s). Staining with Mayer’s hemalum solution (30 s) was followed by washing of samples under tap water (5 min) and counterstaining with eosin (25 s) and rinsing in dH_2_O (80 s). Samples were dehydrated in a graded alcohol series (70–100% ethanol, 80 s each) and processed in xylene (two times, 60 s each). Stained slides were mounted with CV Mount (Leica Biosystems).

Paraffin embedding, slicing and staining were conducted by the histology platform, Cluster of Excellence, ImmunoSensation^2^ at the Medical Faculty, University of Bonn.

### Microscopy of tissue sections

Stained sections were imaged with the Zeiss Axio Scan.Z1 Slide Scanner at the Microscopy Core Facility of the Medical Faculty at the University Bonn.

### Image analysis

Images of histological stainings of adipose tissue were analysed via the AdipoQ pipeline (as previously described in ref. ^85^). Briefly, custom preferences for AdipoQ Preparator and AdipoQ were used to identify and analyse adipocytes.

### mRNA isolation and RT–qPCR analysis

mRNA was isolated from primary adipocytes and adipose tissues were lysed within a monophasic solution of phenol and guanidine isothiocyanate reagent (TRI Reagent, Sigma-Aldrich, T9424) and eluted with RNase/DNase-free water following silica-based RNA spin-column enrichment. RNA concentrations were measured by spectrophotometer and 500 ng RNA was used for reverse transcription (High-Capacity cDNA Reverse Transcription Kit, Thermo Fisher, 4368813). Abundance levels of Adcy3-at, Adcy3-fl, Adipoq, Cidea, Dio2, Elovl3, Emr1, Fabp4, Lsgals3, Pparg isoform 2 (Pparg2), Ppargc1a-at, Ppargc1a-fl, Prdm16 and Ucp1 were quantified using SYBR Green-based quantification method (FastStart Universal SYBR Green Master Mix, Roche Life Science, 4913914001) and mRNA abundance was calculated using relative quantification methods (2 − ΔΔCT). Transcript levels of mRNAs were normalized to hypoxanthine phosphoribosyltransferase 1 (Hprt1) or general transcription factor IIB (Gtf2b) expressions. Primer sequences for SYBR Green-based quantification are provided in Supplementary Table 4.

### Protein isolation

To isolate total protein from 1°BAs and 1°iWAs, 100–250 μl RIPA buffer (50 mM Tris-HCl pH 7.4, 150 mM NaCl, 2 mM EDTA, pH 8.0, 0.1% SDS, 0.1% sodium deoxycholate, 1% NP-40, 1 mM sodium orthovanadate (Na3VO4), 1 mM sodium fluoride (NaF) with protease/phosphatase inhibitor cocktail (Cell Signaling, 5872)) was added to frozen cell pellets harvested from cell culture plates. In case of adipose tissue, Precellys zirconium oxide beads and 200–400 μl RIPA buffer was used to disrupt and homogenize adipose tissue using a FastPrep-24 5 G Homogenizer (mouse muscle programme: 3 × 90 s). To disrupt the cell membrane of the tissue and primary cells, tubes were snap frozen in liquid nitrogen and thawed on ice three times. The samples were centrifuged for 20 min at 13,000*g* and at 4 °C. The supernatant was transferred to a new reaction tube and samples were stored at −80 °C. Quick Start Bradford Protein Assay Kit and Pierce BCA Protein Assay Kit were used for the quantification of protein concentrations according to the instructions of the kits. A CLARIOstar Plus (BMG LABTECH, Germany) microplate reader was used to measure protein concentrations at 595 nM.

### SDS–PAGE and immunoblot analysis

Protein samples (SVF, tissue) were diluted in 4× Laemmli Buffer (1610747, Bio-Rad Laboratories) containing 50 mM dithiothreitol (DTT) and were boiled at 96 °C for 10 min (unless when probing for ADCY3). Protein lysates were separated based on their molecular weight by SDS–PAGE. Samples and PageRuler Prestained Protein Ladder (26619, Thermo Fisher Scientific) were loaded onto precast polyacrylamide (4–20%) gradient gels (Bio-Rad Laboratories), and proteins were separated in an electric field of 200 V for varying time spans in Tris/Glycine/SDS Running Buffer (25 mM Tris base, 192 mM Glycine, 0.1% SDS).

SDS–PAGE-separated proteins were transferred to PVDF or nitrocellulose membranes by wet transfer. Wet transfer was performed in a blotting chamber with an ice-cold transfer buffer (25 mM Tris base, 0.192 mM glycine, 0.1% SDS and 20% ethanol) for 2 h at 100 V. After protein transfer, membranes were incubated in Ponceau solution (59803S, Cell Signaling) to confirm equal loading and transfer. Afterwards, membranes were rinsed once in TBS-T (500 mM Tris base, 1.5 M NaCl, 1% Tween-20, pH 8) and incubated for 1 h in blocking solution (5% milk powder in TBS-T). Membranes were incubated overnight at 4 °C with the respective primary antibody solution (antibodies diluted in 5% BSA in TBS-T). Primary antibodies were anti-HSC70 (sc-7298, Santa Cruz Biotechnology; dilution 1:10,000), anti-UCP1 (14670, Cell Signaling Technology; dilution 1:1,000), anti-ADCY3 (Ab14778, Abcam; dilution 1:500, anti-phospho-PKA Substrate (9624, Cell Signaling Technology; dilution 1:1,000), anti-phospho-CREB (Ser133), (9198, Cell Signaling Technology; dilution 1:1,000), anti-CREB (9197, Cell Signaling Technology, dilution 1:1,000), ani-phospho-HSL (Ser660; 45804, Cell Signaling Technology; dilution 1:1,000) and anti-HSL (4107, Cell Signaling Technology; dilution 1:1,000). Membranes were washed three times for 10 min with TBS-T and incubated for 1 h at RT with the respective horseradish peroxidase (HRP)-coupled secondary antibody (anti-rabbit, 7074, anti-mouse 7076, Cell Signaling Technology). Wash steps were repeated, and 1 ml of chemiluminescent substrate (1705061, Bio-Rad) was applied on the membrane and visualized by Amersham Imager 680.

For CHO K1 and HEK293T cells, 4× sample buffer (200 mM TRIS/HCl pH 6.8, 8% (wt/vol) SDS, 4% (vol/vol) mercaptoethanol, 50% (vol/vol) glycerine, 0.04% (wt/vol) bromphenol blue) was added to the samples at a final concentration of 1×, directly loaded on an 8.75% SDS–PAGE gel, and separated by molecular weight. Subsequently, proteins were transferred onto a methanol-activated PVDF membrane by semi-dry transfer. The membrane was blocked using PBS Intercept blocking buffer (LI-COR) for 30 min at RT. The membrane was then incubated with the primary antibody solution overnight at 4 °C. The following primary antibodies were used: anti-FLAG (dilution 1:2,000; F1804, Sigma-Aldrich) and anti-HA (dilution 1:1,000; Roche, clone 3F10). Following primary antibody incubation, the membrane was washed three times with PBS-T for 10 min at RT. The membrane was then incubated with the respective IRDye secondary antibodies (dilution 1:20,000, LI-COR) for 1 h at RT. After washing the membrane three times with PBS-T and once with PBS, protein bands were imaged using a LI-COR imaging system. Protein bands were quantified using ImageJ.

### Protein detection by Simple Western Analysis

Whole protein lysates of EndoH-treated membrane fractions from CHO K1 and HEK293T cells were analysed according to the manufacturer’s protocol using the capillary immunoassay WES system (ProteinSimple). Lysates were mixed with a fluorescent master mix (ProteinSimple) to a final concentration of 1× sample buffer, 1× fluorescent master mix and 40 mM DTT. Samples, blocking reagents, primary antibodies and HRP-conjugated secondary antibodies, as well as the chemiluminescent substrate (ProteinSimple), were loaded into a 13-well plate and automatically analysed in the capillary immunoblotting system. Primary antibodies directed against the C terminus of AC3 (dilution 1:1:5,000; Thermo Fisher Scientific, PA5-35382) and against the HA-tag (dilution 1:1,000; Roche, 11867423001) were prepared in antibody diluent 2 (ProteinSimple). Samples, blocking reagents, primary antibodies and HRP-conjugated secondary antibodies, as well as the chemiluminescent substrate (ProteinSimple), were loaded into a 13-well plate and automatically analysed in the capillary immunoblotting system.

### Co-immunoprecipitation

2.1 × 10^6^ CHO K1 cells stably expressing mAC3-HA or mAC3-AT-FLAG were seeded on 10-cm cell culture dishes (Greiner). The following day, the cells were transfected with pc3.1-Adcy3-AT-FLAG, pc3.1-mAdcy6-FLAG or pc3.1Zeo-mCherry using polyethylenimine (Sigma-Aldrich) or Lipofectamine 2000 Transfection Reagent (Thermo Fisher Scientific). To increase expression of the transfected constructs in polyethylenimine-transfected cells, 5 h after the transfection sodium butyrate was added to the cells to a final concentration of 5 µM. Next, 2.1 × 10^6^ HEK293T cells were seeded on 10-cm cell culture dishes (Greiner). The following day, the cells were transfected with pRP-hAdcy3-HA and pRP-hAdcy3-AT-FLAG, or pc3.1Zeo-mCherry. To increase expression of the transfected constructs in polyethylenimine-transfected cells, 5 h after the transfection sodium butyrate was added to the cells to a final concentration of 5 µM. The following day, or 24 h later, both CHO K1 and HEK293T cells were washed with 10 ml PBS and scraped in 1 ml PBS. The cell solution was then transferred into a precooled tube and centrifuged at 500*g* for 5 min at 4 °C. The supernatant was aspirated, and the cell pellet was resuspended in 300 µl lysis buffer (20 mM TRIS/HCl pH 8, 137 mM NaCl, 2 mM EDTA, 1% NP-40, 1:500 dilution of mammalian protease inhibitor cocktail (Sigma-Aldrich)). Following 60 min of incubation on ice, the samples were centrifuged at 10,000*g* for 10 min at 4 °C. The supernatant was transferred into a new tube, and the protein concentration was determined using the Pierce BCA Protein Assay Kit (Thermo Scientific). Protein concentration was adjusted to 0.5 µg µl^−1^ using lysis buffer and 45 µl were stored at −80 °C (‘input’). To reduce the amount of nonspecific binding, the lysate was incubated with uncoupled NHS-activated magnetic beads (Thermo Fisher). Around 40 µl NHS-activated beads were equilibrated in 400 µl equilibration buffer (10 mM TRIS/HCl pH 7.4, 150 mM NaCl) and separated from the supernatant using a magnet. The beads were resuspended in 500 µl of the 0.5 µg µl^−1^ lysate and incubated for 1 h at 4 °C end over end. Beads and lysate were then magnetically separated, and 45 µl of the supernatant was stored at −80 °C (‘non-bound 1’). Magnetic anti-FLAG-M2 beads (Sigma-Aldrich) were equilibrated in 400 µl equilibration buffer (10 mM Tris/HCl pH 7.4, 150 mM NaCl), resuspended in the supernatant, and incubated in end over end overnight at 4 °C. The next day, beads and supernatant were magnetically separated, 45 µl of the supernatant was stored at −80 °C (‘non-bound 2’), and the rest of supernatants were discarded. The beads were washed four times with 375 µl wash buffer (20 mM TRIS/HCl, pH 8, 137 mM NaCl, 2 mM EDTA, 0.2% NP-40, 1.500 dilution of mammalian protease inhibitor cocktail). Around 45 µl of wash fractions 1 and 4 was saved for immunoblot analysis. Proteins bound to the beads were eluted by incubating the beads in 75 µl elution buffer (0.1 M glycine, pH 3.0) for 3 min at RT. Subsequently, 20 µl 1 M TRIS/HCl, pH 8, was added for neutralization. Then, 45 µl of the neutralized elution fraction was analysed by imunoblot analysis.

### Biotinylation assay

The biotinylation assay was performed using the Pierce Cell Surface Biotinylation and Isolation kit (A44390, Thermo Scientific). Cells were treated with the biotinylation reagent Sulfo-NHS-SS-Biotin according to the manufacturer’s protocol, harvested and lysed. The lysate was transferred to a column containing a NeutrAvidin-agarose-resin and incubated end over end overnight. The column was centrifuged and the flow-through (sample F) was collected. The column was washed three times (samples W1, W2 and W3) with 500 µl wash buffer, and the labelled protein was eluted (sample E) using 200 µl 4× SDS sample buffer containing 50 mM DTT. After elution, beads of the agarose matrix were scraped off the column, boiled in 100 µl 4× SDS probe buffer and centrifuged. For each fraction, 40 µl was loaded. To quantify and analyse the western blots from the biotinylation assay, we determined the band intensities by densitometric analysis for input and eluate using ImageJ by taking both bands of the double band into account. As we loaded 40 µl from 300 µl (13%) of the input and 40 µl from 200 µl (20%) of the elution sample, intensity values were normalized accordingly, and the ratio of eluate to the input sample was calculated.

### Membrane sheets and immunofluorescence labelling

CHO cells were seeded on poly-l-lysine (0.1 mg ml^−1^; Sigma-Aldrich, P1399-100MG)-coated 13-mm glass coverslips (VWR) in a four-well dish (VWR) and cultured for 24 h. Before sonification, cells were washed with PBS and sonicated in 500 µl sonication buffer (120 mM glutamate, 20 mM potassium acetate, 10 mM HEPES, 10 mM EGTA, pH 7.2, using the VibraCell Sonifier (0.1 s pulse, distance to coverslip of 3 mm, amplitude of 1–20%). Cells were fixed with 4% paraformaldehyde (Alfa Aesar, Thermo Fisher Scientific, 43368) for 10 min at RT. After washing thrice with PBS, cells were blocked with CT (0.5% Triton X-100 (Sigma-Aldrich, X-100) and 5% ChemiBLOCKER (Merck Millipore, 2170) in 0.1 M sodium phosphate, pH 7.0) for 30 min at RT. Primary and secondary antibodies were diluted in CT and incubated for 60 min each at RT, respectively. Coverslips were mounted with one drop of Aqua-Poly/Mount (Tebu-Bio, 07918606-20). The following primary antibodies were used: anti-calnexin (rb, dilution 1:200; Sigma-Aldrich, C4731), anti-HA (rt, dilution 1:1,000, Roche, clone 3F10) and anti-CNG channel (ms, dilution 1:100; clone 3B10; ref. ^86^). As a DNA counterstain, cells were labelled with DAPI together with the secondary antibody (4’,6-diamidino-2-phenylindole, dihydrochloride, dilution 1:10,000; Thermo Fisher Scientific, D1306). The following secondary antibodies were used: goat-anti-rabbit-Alexa 647 (dilution 1:500; Thermo Fisher Scientific, A21245), goat-anti-mouse-Alexa Fluor 594 (dilution 1:500; Thermo Fisher Scientific, A21125) and goat-anti-rat-Alexa Fluor 488 (dilution 1:500; Thermo Fisher Scientific, A11006).

### PamGene STK kinome arrays

Frozen BAT was homogenized in 150 µl cold M-Per (Thermo Fisher) containing a 1:50 dilution of HALT Protease and Phosphatase Inhibitor Cocktail (EDTA-free, 100×, Thermo Fisher) using a small, precooled pistil. Following 10 min of incubation on ice, samples were centrifuged at 10,000*g* for 5 min at 4 °C. The liquid phase was carefully transferred into a new precooled tube, and this process was repeated at least twice (or until no lipid phase was left). Subsequently, the cleared lysates were aliquoted in 15-µl aliquots, frozen in liquid nitrogen, and stored at −80 °C. Not more than four samples were handled simultaneously to avoid prolonged handling times. After the protein concentration was determined using Pierce BCA Protein Assay Kit (Thermo Scientific), 10 µg of protein was used as input and the STK assay was performed according to the standard protocol provided by PamGene. Kinome trees were generated using software available at http://phanstiel-lab.med.unc.edu/CORAL/.

### CatchPoint cAMP assay (ELISA)

BAT and differentiated 1°BA cAMP levels were determined using the CatchPoint cAMP Fluorescent Assay Kit (Molecular Devices). A small piece of frozen BAT (2–5 mg) was transferred into a BeadBug tube (Sigma-Aldrich) containing 1.0-mm zirconium beads (Merck) and 300 µl cold CatchPoint lysis buffer. The tissue was disrupted using a BeadBug microtube homogenizer (Sigma-Aldrich) at 400 rpm for 30 s. Subsequently, samples were placed on ice for 1 min and homogenization was repeated twice. Following 15 min of incubation at 4 °C, each sample was sonicated three times for 1 min with 1 min of incubation on ice in between each cycle. To clear the lysate from debris and insoluble lipids, samples were centrifuged at 10,000*g* for 10 min at 4 °C. The liquid fraction of the lysate was then transferred into a new tube and the process was repeated twice. The protein concentration of the cleared lysate was determined using the Pierce BCA Protein Assay Kit (Thermo Scientific). 1°BAs were harvested in 300 µl of cold CatchPoint lysis buffer and incubated for 15 min at 4 °C. To clear the lysate from cell debris and insoluble lipids, samples were centrifuged at 10,000*g* for 10 min at 4 °C. The CatchPoint cAMP ELISA was then performed according to the manual using 0.8 µg protein as input. Colorimetric measurement was performed after a 30-min final incubation using a FLUOstar Omega (BMG Labtech).

### Human primary brown and white adipocytes and study characteristics

Deep-neck BAT biopsy samples were acquired from a 52-year-old, nondiabetic female donor (body mass index of 24.1) undergoing thyroid surgery after giving written informed consent and approval by the ethics committee of the University Hospital Bonn (Vote 076/18). 1°BAs were isolated according to protocols described recently (Jespersen et al., 2013) and cultured in 60-mm culture dishes containing DMEM/F-12, 10% FBS, 1% penicillin–streptomycin (all from Invitrogen) and 1 nM acidic FGF-1 (ImmunoTools)^87^. Cells were incubated at 37 °C with 5% CO_2_. Adipocytes were induced 2 days after full confluence with DMEM/F-12 containing 1% penicillin–streptomycin, 0.1 mM dexamethasone (Sigma-Aldrich), 100 nM insulin, 200 nM rosiglitazone (Sigma-Aldrich), 540 mM isobutylmethylxanthine (Sigma-Aldrich), 2 nM T3 (Sigma-Aldrich) and 10 mg ml^−1^ transferrin (Sigma-Aldrich). After 3 days of differentiation, isobutylmethylxanthine was removed from the cell culture media. The cell cultures were left to differentiate for an additional 9 days. Cells were treated with and without 1 µM NE for 16 h. Total RNA was isolated using TRIzol (Invitrogen). Reverse transcription was performed using ProtoScript II (NEB). qPCR reactions were assembled with Luna Master Mix (NEB), and qPCR was performed using a HT7900 (Applied Biosystems). Expression levels were calculated as delta Ct values relative to housekeeping gene hTBP (human TATA-box binding protein) serving as control.

### Comparative sequence conservation analyses of *Adcy3-at* and *Ppargc1a-at* genomic sequences

Analyses of alternative transcription (AT) start sites sequence conservation were conducted by first acquiring genomic contigs spanning either *Ppargc1a* or *Adcy3* via NCBI BLASTs using mouse or human transcript variants (accession numbers NM_001377131.1, NM_138305.3 and NR_132764.1) as a query against whole-genome shotgun contigs from representative species. Accession numbers of acquired contigs are listed in Supplementary Table 2. Contigs were imported into Geneious software version 9.1.8 (Dotmatics), and conserved exons were annotated using the ‘transfer annotations’ function. Alternative start sites from the mouse or human were then used to search for homologous sequences among contigs of various species using either the ‘transfer annotations’ function or EMBOSS 6.5.7 dotmatcher dot plots. MUSCLE alignments of conserved putative AT regions were assembled in Geneious and the pairwise percentage of nucleotide identities was calculated relative to AT sites of either the mouse or human. These conservation levels were then mapped to the respective phylogenetic trees using the colour gradient function on the Interactive Tree of Life webserver^88^. The phylogenetic relationships and branch lengths illustrating the conservation of *Ppargc1a-at* were based on ref. ^89^, while phylogenetic relationships and branch lengths for *Adcy3-at* conservation analyses were based on ref. ^90^ for primates and rodents^91^.

### Statistical considerations

No statistical methods were used to predetermine sample sizes, but our sample sizes are similar to those reported in previous publications and empirical data from our labs^40,92^. No normality tests were performed for samples sizes *n* ≤ 5, but non-parametric tests were chosen, and individual data points plotted. For sample sizes ≥6, data distribution was assumed to be normal, but these were not formally tested. Data collection for animal experiments was not randomized due to the need to house litters of male mice within the same cage.

### Reporting summary

Further information on research design is available in the Nature Portfolio Reporting Summary linked to this article.

## Supplementary information

Reporting Summary

Supplementary Tables 1–6 Multi-tabs workbook with Supplementary Tables 1–6. Supplementary Table 1: Adipose depot-specific RNA-seq from cold-exposed C57BL/6 mice. Supplementary Table 2: Gene accession numbers for species conservation analyses. Supplementary Table 3: List of sgRNAs and primers for the generation of *Adcy3∆AT* mice. Supplementary Table 4: List of SYBR qPCR primers. Supplementary Table 5: List of LNA GapmeR inhibitors. Supplementary Table 6: Alignment mAC3/mAC3-AT.

## Source data

Source Data Fig. 1 Statistical source data.

Source Data Fig. 2 Statistical source data.

Source Data Fig. 3 Statistical source data.

Source Data Fig. 4 Statistical source data.

Source Data Fig. 5 Statistical source data.

Source Data Fig. 6 Statistical source data.

Source Data Fig. 7 Statistical source data.

Unprocessed blots main figures Uncropped blots for Figs. 1–7.

Unprocessed blots extended data figures Uncropped blots for Extended Data Figs. 1–9

Source Data Extended Data Fig./Table 1 Statistical source data.

Source Data Extended Data Fig./Table 2 Statistical source data.

Source Data Extended Data Fig./Table 3 Statistical source data.

Source Data Extended Data Fig./Table 4 Statistical source data.

Source Data Extended Data Fig./Table 5 Statistical source data.

Source Data Extended Data Fig./Table 6 Statistical source data.

Source Data Extended Data Fig./Table 7 Statistical source data.

Source Data Extended Data Fig./Table 8 Statistical source data.

Source Data Extended Data Fig./Table 9 Statistical source data.

## Data Availability

Illumina datasets from RT and cold-activated eWAT and iBAT are available at the Gene Expression Omnibus under accession GSE212574. H3K4me3 ChIP–seq data from BAT of chow diet-fed and HFD-fed male C57BL/6N mice housed at 22 °C or exposed to 5 °C for 24 h were downloaded from the Gene Expression Omnibus (GSE20065). snRNA-seq data from Adipoq-tdTomato-positive adipocyte nuclei were downloaded from ArrayExpress (E-MTAB-8562). All immunofluorescence images have been uploaded on figshare 10.6084/m9.figshare.c.6725808.v1Source data are provided with this paper.
