# Publisher Correction: Cold-induced expression of a truncated adenylyl cyclase 3 acts as rheostat to brown fat function

**DOI:** 10.1038/s42255-025-01292-z

**Published:** 2025-04-04

**Authors:** Sajjad Khani, Hande Topel, Ronja Kardinal, Ana Rita Tavanez, Ajeetha Josephrajan, Bjørk Ditlev Marcher Larsen, Michael James Gaudry, Philipp Leyendecker, Nadia Meincke Egedal, Aylin Seren Güller, Natasa Stanic, Phillip M. M. Ruppert, Isabella Gaziano, Nils Rouven Hansmeier, Elena Schmidt, Paul Klemm, Lara-Marie Vagliano, Rainer Stahl, Fraser Duthie, Jens-Henning Krause, Ana Bici, Christoph Andreas Engelhard, Sabrina Gohlke, Peter Frommolt, Thorsten Gnad, Alvaro Rada-Iglesias, Marta Pradas-Juni, Tim Julius Schulz, Frank Thomas Wunderlich, Alexander Pfeifer, Alexander Bartelt, Martin Jastroch, Dagmar Wachten, Jan-Wilhelm Kornfeld

**Affiliations:** 1 Institute for Genetics, University of Cologne, Cologne, Germany; 2 Max Planck Institute for Metabolism Research, Cologne, Germany; 3 Department for Biochemistry and Molecular Biology, University of Southern Denmark, Odense, Denmark; 4 Novo Nordisk Foundation Center for Adipocyte Signaling (Adiposign), University of Southern Denmark, Odense, Denmark; 5 Institute of Innate Immunity, Medical Faculty, University of Bonn, Bonn, Germany; 6 Department of Molecular Biosciences, The Wenner-Gren Institute, Stockholm University, Stockholm, Sweden; 7 Institute for Cardiovascular Prevention (IPEK), Ludwig-Maximilians-University, Munich, Germany; 8 Centre for Physical Activity Research, Department of Infectious Diseases, Rigshospitalet, Faculty of Health Sciences, University of Copenhagen, Copenhagen, Denmark; 9 Department of Adipocyte Development and Nutrition, German Institute of Human Nutrition Potsdam-Rehbrücke, Nuthetal, Germany; 10 Institute of Human Genetics, University Medical Center Hamburg-Eppendorf, Hamburg, Germany; 11 Institute of Pharmacology and Toxicology, University Hospital, University of Bonn, Bonn, Germany; 12 Institute of Biomedicine and Biotechnology of Cantabria (IBBTEC), CSIC/University of Cantabria, Santander, Spain; 13 Novo Nordisk Foundation Center for Basic Metabolic Research (CBMR), Copenhagen, Denmark; 14 German Center for Diabetes Research (DZD), München-Neuherberg, Germany; 15 Institute for Diabetes and Cancer (IDC), Helmholtz Center Munich, German Research Center for Environmental Health, Neuherberg, Germany; 16 German Center for Cardiovascular Research (DZHK), Partner Site Munich Heart Alliance, Munich, Germany; 17 Department of Molecular Metabolism and Sabri Ülker Center for Metabolic Research, Harvard T.H. Chan School of Public Health, Boston, MA USA

**Keywords:** Metabolic diseases, Molecular medicine, Metabolism, Fat metabolism, Enzyme mechanisms

Correction to: *Nature Metabolism* 10.1038/s42255-024-01033-8, published online 29 April 2024.

This article was originally published under standard Springer Nature license (© The Author(s), under exclusive licence to Springer Nature Limited). It is now available as an open-access paper under a Creative Commons Attribution 4.0 International license, © The Author(s). The error has been corrected in the HTML and PDF versions of the article.

