# Supplementary material for: Cold-induced expression of a truncated adenylyl cyclase 3 acts as rheostat to brown fat function
Source: Nat Metab. 2024 Apr 29;6(6):1053–75. doi: 10.1038/s42255-024-01033-8 (PMC11971047; doi:10.1038/s42255-024-01033-8)
Supplement: Supplementary file 1 — Reporting Summary [file 42255_2024_1033_MOESM1_ESM.pdf]

nature portfolio

Corresponding author(s): Jan-Wilhelm Kornfeld, Dagmar Wachten

Last updated by author(s): 6/3/2024

Reporting Summary

Nature Portfolio wishes to improve the reproducibility of the work that we publish. This form provides structure for consistency and transparency in reporting. For further information on Nature Portfolio policies, see our Editorial Policies and the Editorial Policy Checklist.

Statistics

For all statistical analyses, confirm that the following items are present in the figure legend, table legend, main text, or Methods section.

n/a	Confirmed	
☐	☒	The exact sample size (*n*) for each experimental group/condition, given as a discrete number and unit of measurement
☐	☒	A statement on whether measurements were taken from distinct samples or whether the same sample was measured repeatedly
☐	☒	The statistical test(s) used AND whether they are one- or two-sided<br>*Only common tests should be described solely by name; describe more complex techniques in the Methods section.*
☒	☐	A description of all covariates tested
☐	☒	A description of any assumptions or corrections, such as tests of normality and adjustment for multiple comparisons
☐	☒	A full description of the statistical parameters including central tendency (e.g. means) or other basic estimates (e.g. regression coefficient) AND variation (e.g. standard deviation) or associated estimates of uncertainty (e.g. confidence intervals)
☐	☒	For null hypothesis testing, the test statistic (e.g. *F*, *t*, *r*) with confidence intervals, effect sizes, degrees of freedom and *P* value noted<br>*Give P values as exact values whenever suitable.*
☒	☐	For Bayesian analysis, information on the choice of priors and Markov chain Monte Carlo settings
☒	☐	For hierarchical and complex designs, identification of the appropriate level for tests and full reporting of outcomes
☒	☐	Estimates of effect sizes (e.g. Cohen's *d*, Pearson's *r*), indicating how they were calculated

Our web collection on statistics for biologists contains articles on many of the points above.

Software and code

Policy information about availability of computer code

Data collection	All NGS datasets are being uploaded to Gene Expression Omnibus as Gene Expression Omnibus (GEO) series GSE212574
Data analysis	Analysis of NGS datasets (mRNA-seq, Oxford Nanopore Technology full-length RNA/cDNA-seq and ChiP-seq) uses publicly available algorithms widely used in the field. Specific information relevant to the software packages are provided in the Methods section of the paper.

For manuscripts utilizing custom algorithms or software that are central to the research but not yet described in published literature, software must be made available to editors and reviewers. We strongly encourage code deposition in a community repository (e.g. GitHub). See the Nature Portfolio guidelines for submitting code & software for further information.

Data

Policy information about availability of data

All manuscripts must include a data availability statement. This statement should provide the following information, where applicable:

- Accession codes, unique identifiers, or web links for publicly available datasets
- A description of any restrictions on data availability
- For clinical datasets or third party data, please ensure that the statement adheres to our policy

All NGS datasets are being uploaded to Gene Expression Omnibus as Gene Expression Omnibus (GEO) series GSE212574

Human research participants

Policy information about studies involving human research participants and Sex and Gender in Research.

Reporting on sex and gender	Human brown adipocytes were obtained from deep neck BAT biopsies. These were acquired from a 52-year-old, non-diabe Deep neck BAT biopsies were acquired from a 52-year-old, non-diabetic female donor (BMI 24.1) undergoing thyroid surgery after giving written informed consent and approval by the ethics committee of the University Hospital Bonn (Vote 076/18).
Population characteristics	Not applicable
Recruitment	Not applicable
Ethics oversight	Approval by the ethics commitee of the University Hospital Bonn (Vote 076/18).

Note that full information on the approval of the study protocol must also be provided in the manuscript.

Field-specific reporting

Please select the one below that is the best fit for your research. If you are not sure, read the appropriate sections before making your selection.

☒ Life sciences ☐ Behavioural & social sciences ☐ Ecological, evolutionary & environmental sciences

For a reference copy of the document with all sections, see nature.com/documents/nr-reporting-summary-flat.pdf

Life sciences study design

All studies must disclose on these points even when the disclosure is negative.

Sample size	We performed in vivo studies in sample size of at least n=3 experimental animals per genotype and diet group, but typically more.<br>In vitro experiments were performed in at least biological triplicates, typically each in technical triplicates.
Data exclusions	Animals were excluded from glucose tolerance tests when blood glucose levels did not rise after injecting glucose into the peritoneal cavity. According to our experience this is typically due to malinjection of glucose, e.g. into visceral organs. Animals were excluded from the experiments when suffering under HFD-induced skin rashes or wounds from fighting.<br><br>Those animals that showed no increase/decrease of blood glucose levels after i.p. injection of glucose or insulin, assuming injection outside of the peritoneal cavity as required for the assay, were excluded from analysis (intraperitoneal insulin tolerance test)
Replication	All attempts at replication were successful at the sample group level.
Randomization	Animals were not randomized before the study due to the necessity to continue housing littermate males. Randomization and new combinations of males in a cage often lead to stress and fighting to establish social hierarchy.
Blinding	The experimentators were not blinded to the genotype of the animals. Diet effects included body weight gains after high-fat feeding and thus the experimentator could not be blinded.

Reporting for specific materials, systems and methods

We require information from authors about some types of materials, experimental systems and methods used in many studies. Here, indicate whether each material, system or method listed is relevant to your study. If you are not sure if a list item applies to your research, read the appropriate section before selecting a response.

Materials & experimental systems

n/a	Involved in the study	
☐	☒	Antibodies
☐	☒	Eukaryotic cell lines
☒	☐	Palaeontology and archaeology
☐	☒	Animals and other organisms
☒	☐	Clinical data
☒	☐	Dual use research of concern

Methods

n/a	Involved in the study	
☐	☒	ChIP-seq
☒	☐	Flow cytometry
☒	☐	MRI-based neuroimaging

Antibodies

Antibodies used	Primary antibodies were anti-HSC70 (sc-7298, Santa Cruz Biotechnology, dilution 1:10,000), anti-UCP1 (#14670, Cell Signaling Technology, dilution 1:1000), anti-ADCY3 (#Ab14778, Abcam, dilution 1:500, and anti-phospho-PKA Substrate (#9624, Cell Signaling Technology, 1:1000). anti-phospho-HSL(Ser660) (#45804, Cell Signaling Technology, dilution 1:1000) and anti-HSL (#4107, Cell Signaling Technology, dilution 1:1000). anti-calnexin (#C4731, Sigma Aldrich, dilution 1:200), anti-CNG channel (clone 3B10) (Pichlo et al, JCB 2014), dilution 1:200), anti-C-terminus of AC3 (PA5-35382, Thermofisher Scientific, dilution 1:5000) ,anti-HA-tag (11867423001, Roche, dilution 1:1000), anti-FLAG (F1804, Sigma-Aldrich, dilution 1:2000) , anti-RFP (600-401-379, Rockland Inc., dilution 1:1000). These antibodies are routinely used in the lab and are referenced in several scientific publications.
Validation	The antibodies were not validated.

Eukaryotic cell lines

Policy information about cell lines and Sex and Gender in Research

Cell line source(s)	Chinese Hamster Ovary (CHO) cells: ATCC, #CCL-61, Human Embryonic Kidney (HEK293T) (ATCC, #CRL-3216)
Authentication	Not applicable
Mycoplasma contamination	Regularly tested for mycoplasm
Commonly misidentified lines (See ICLAC register)	Not applicable

Animals and other research organisms

Policy information about studies involving animals; ARRIVE guidelines recommended for reporting animal research, and Sex and Gender in Research

Laboratory animals	Experimental animals were kept in individually ventilated cages (IVC Type II long) in a pathogen-free (SPF) animal facility with controlled temperature (22-24 °C), light/dark cycle (12h/12h) and humidity (50-70%). All animals were maintained and regularly backcrossed to a C57BL/6N background and housed in groups of 3-4 animals per cage and had ab libitum access to food and drinking water. All mice were sacrificed by cervical dislocation or carbon dioxide asphyxiation. Unless otherwise indicated, animals were allowed ad libitum access to chow diet (ssniff® R/M-H Low-Phytoestrogen, V1554) containing 62 kJ% carbohydrates, 27 kJ% protein and 11 kJ% fat and drinking water. Diet-induced obesity (DIO) was achieved by feeding a high-fat diet (HFD, D12492 (I) mod; Sniff) containing 22 kJ% carbohydrates, 24k J% protein and 54 kJ% fat from starting at 6-8 weeks of age.<br><br>Adcy3 floxed mice were kindly provided by Chen and colleagues. Herein, exon 3 of the Adcy3 gene is flanked by two intronic LoxP sites, each 75 base pairs upstream or downstream of the exon. Deleting exon 3 of Adcy3 causes a frame shift mutation, resulting in a premature stop codon within the Adcy3 gene. Adipose tissue-specific deletion of both, Adcy3-fl and Adcy3-at, isoforms of Adcy3 was achieved using cre recombinase-mediated excision of LoxP-flanked ('floxed') gene sequences. For this, mice floxed for Adcy3 (Adcy3LoxP/LoxP) were interbred with mice expressing the Adipoq-cre recombinase under control of mature adipocyte specific Adipoq promote (Adipoq-cre+/cre). AdipoQ-Cre mice were obtained from Jax (Stock no. #010803) and backcrossed to C57BL/6N for at least five generations. Resulting (Adcy3LoxP/LoxP, Adipoq-cre+/cre mice (Adcy3-AdcKO) were compared to (Adcy3LoxP/LoxP, Adipoq-cre+/+) littermates as controls (LoxP).<br><br>Adcy3ΔAT knock-out ES cells were generated using CRISPR/Cas9 technology. Details about generation and validation of this novel mouse line are provided in the paper.
Wild animals	This study did not involve wild animals.
Reporting on sex	Findings were obtained exlusively in male mice. Due to the large number of experimental animals and limitations in animal handling and phenotyping, we decided to limit this study to the analysis of male animals unless otherwise indicated.
Field-collected samples	The study does not include samples collected from the field.
Ethics oversight	Care of animals was within institutional and animal-care committee guidelines approved by (1) local (Bezirksregierung Köln) or regional (Tierschutzkommission acc. §15 TSchG of Landesamt for Natur, Umwelt und Verbraucherschutz (LANUV) North-Rhine Westphalia, Germany) authorities, internal accession no. 84-02.04.2017.A009 or (2) Ministry of Environment of Denmark (Miljø- og Fødevarestyrelsen), internal accession no. 2018-15-0201-01562.

Note that full information on the approval of the study protocol must also be provided in the manuscript.

ChIP-seq

Data deposition

☒ Confirm that both raw and final processed data have been deposited in a public database such as GEO.

☐ Confirm that you have deposited or provided access to graph files (e.g. BED files) for the called peaks.

Data access links<br>*May remain private before publication.*	Data are from a published study (Engelhard, C.A. et al. Comprehensive Transcriptional Profiling and Mouse Phenotyping Reveals Dispensable Role for Adipose Tissue Selective Long Noncoding RNA Gm15551. Noncoding RNA 8 (2022))
Files in database submission	Not applicable
Genome browser session<br>(e.g. UCSC)	Not applicable

Methodology

Replicates	Not applicable
Sequencing depth	Not applicable
Antibodies	Not applicable
Peak calling parameters	Not applicable
Data quality	Not applicable
Software	Not applicable
