# Supplementary material for: Cold-induced expression of a truncated adenylyl cyclase 3 acts as rheostat to brown fat function
Source: Nat Metab. 2024 Apr 29;6(6):1053–75. doi: 10.1038/s42255-024-01033-8 (PMC11971047; doi:10.1038/s42255-024-01033-8)
Supplement: Supplementary file 10 — Uncropped blots for Figs. 1–7. [file 42255_2024_1033_MOESM10_ESM.pdf]

Figure 3L

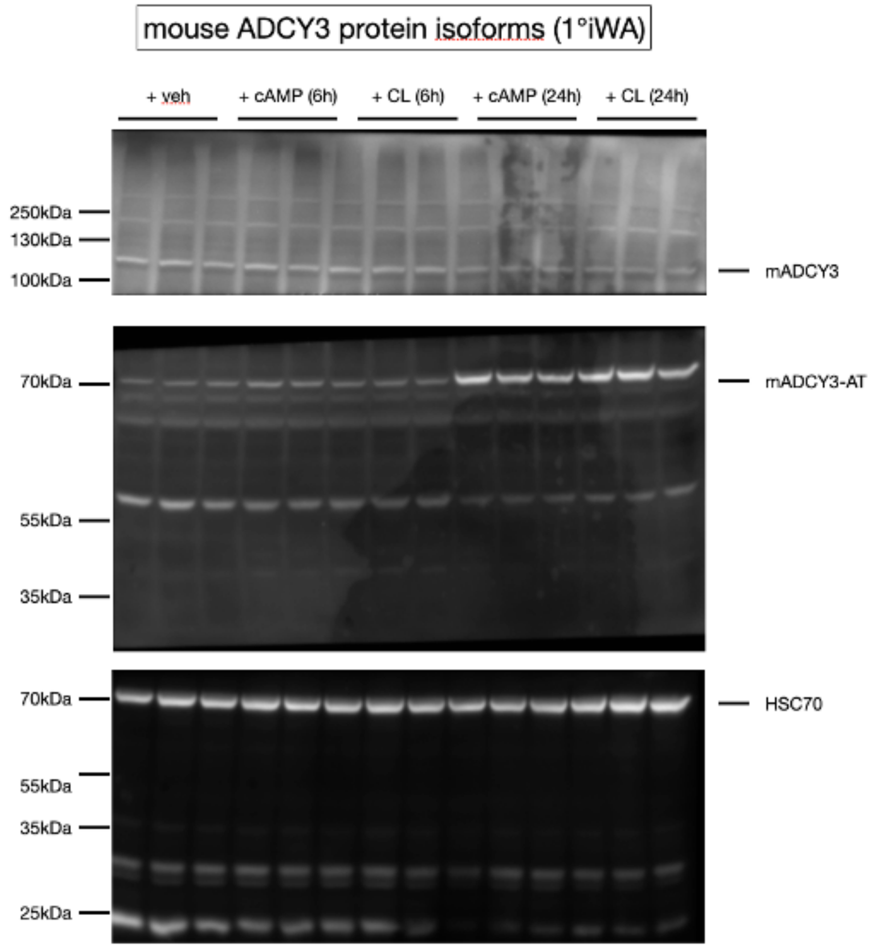

mouse ADCY3 protein isoforms (1°iWA)
+ veh
+ cAMP (6h)
+ CL (6h)
+ cAMP (24h)
+ CL (24h)
250kDa
130kDa
100kDa
mADCY3
70kDa
55kDa
35kDa
mADCY3-AT
70kDa
55kDa
35kDa
25kDa
HSC70

Figure 3M

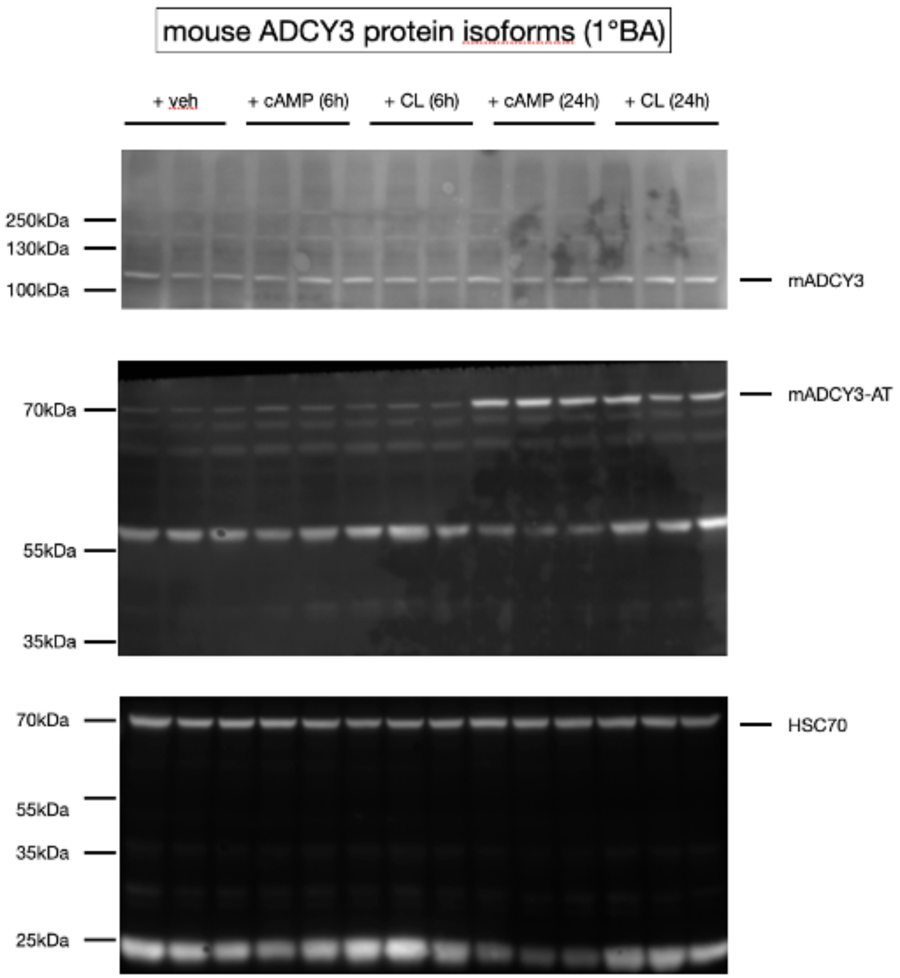
mouse ADCY3 protein isoforms (1°BA)
+ veh
+ cAMP (6h)
+ CL (6h)
+ cAMP (24h)
+ CL (24h)
250kDa
130kDa
100kDa
mADCY3
mADCY3-AT
70kDa
55kDa
35kDa
70kDa
HSC70
55kDa
35kDa
25kDa

Figure 4M

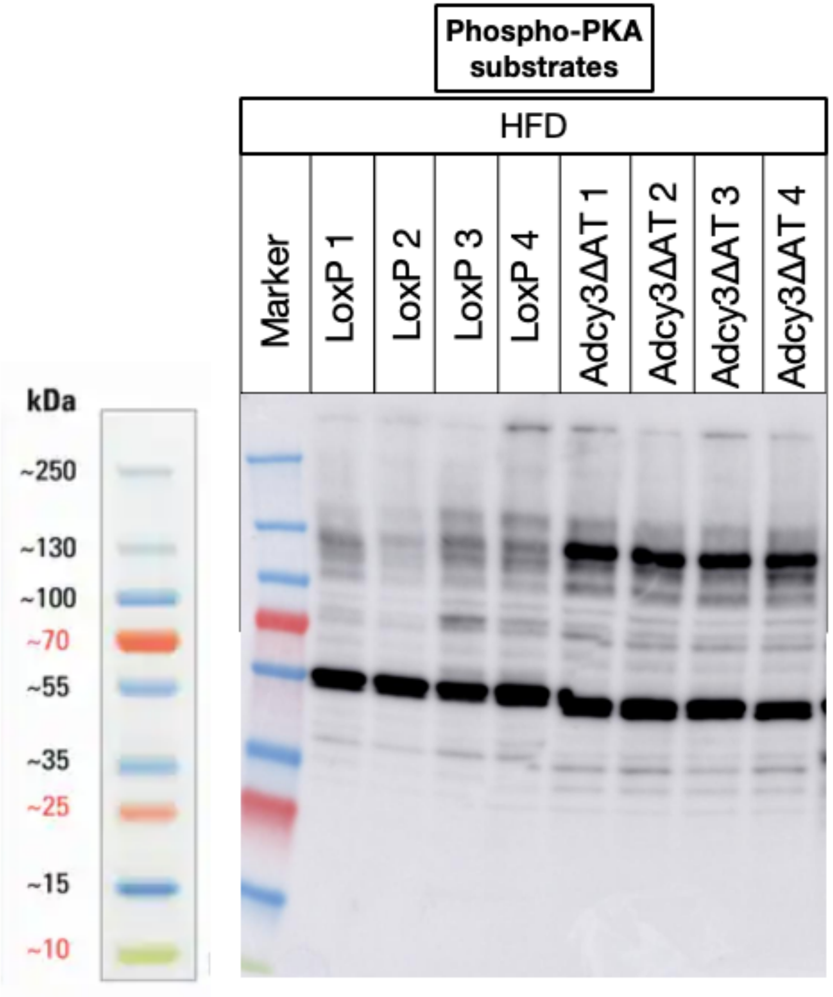

Phospho-PKA substrates
HFD
Marker
LoxP 1
LoxP 2
LoxP 3
LoxP 4
Adcy3ΔAT 1
Adcy3ΔAT 2
Adcy3ΔAT 3
Adcy3ΔAT 4
kDa
~250
~130
~100
~70
~55
~35
~25
~15
~10

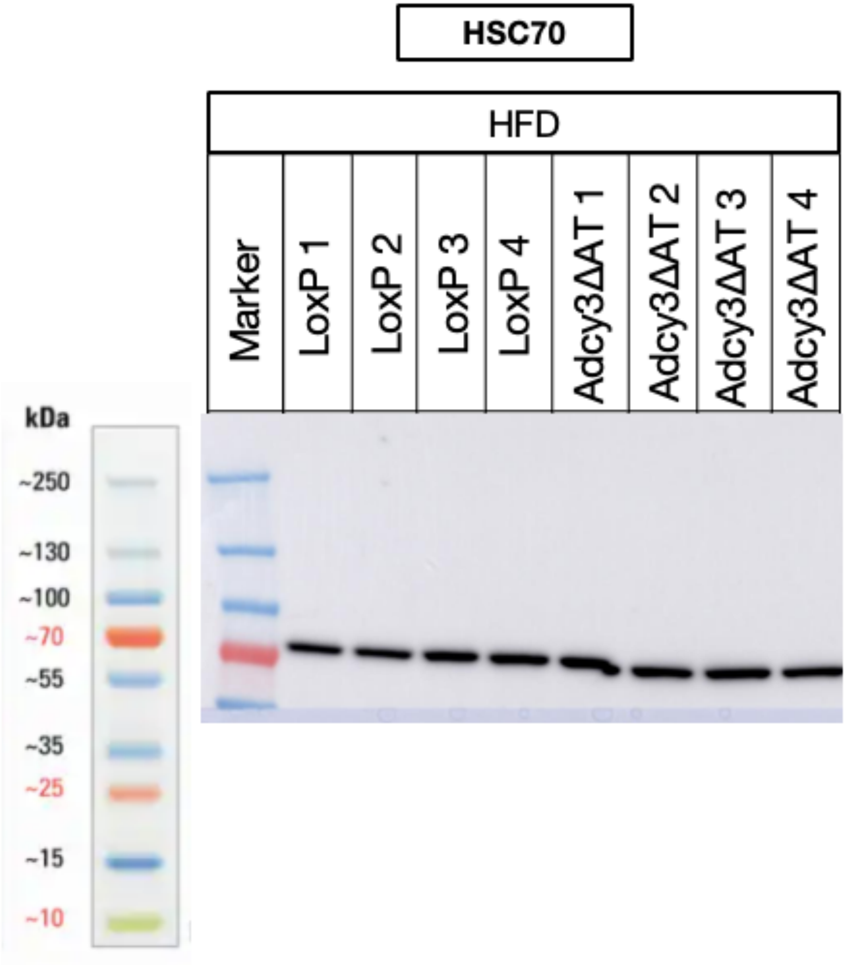

HSC70
HFD
Marker
LoxP 1
LoxP 2
LoxP 3
LoxP 4
Adcy3ΔAT 1
Adcy3ΔAT 2
Adcy3ΔAT 3
Adcy3ΔAT 4
kDa
~250
~130
~100
~70
~55
~35
~25
~15
~10

Figure 4N

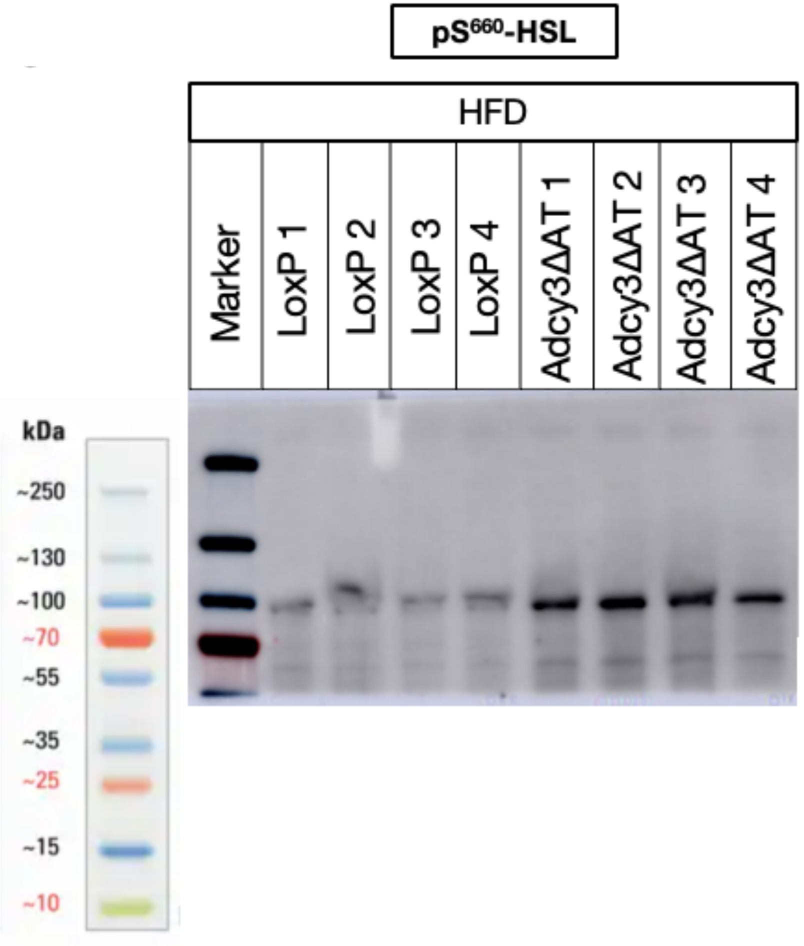
pS660-HSL
HFD
Marker
LoxP 1
LoxP 2
LoxP 3
LoxP 4
Adcy3ΔAT 1
Adcy3ΔAT 2
Adcy3ΔAT 3
Adcy3ΔAT 4
kDa
~250
~130
~100
~70
~55
~35
~25
~15
~10

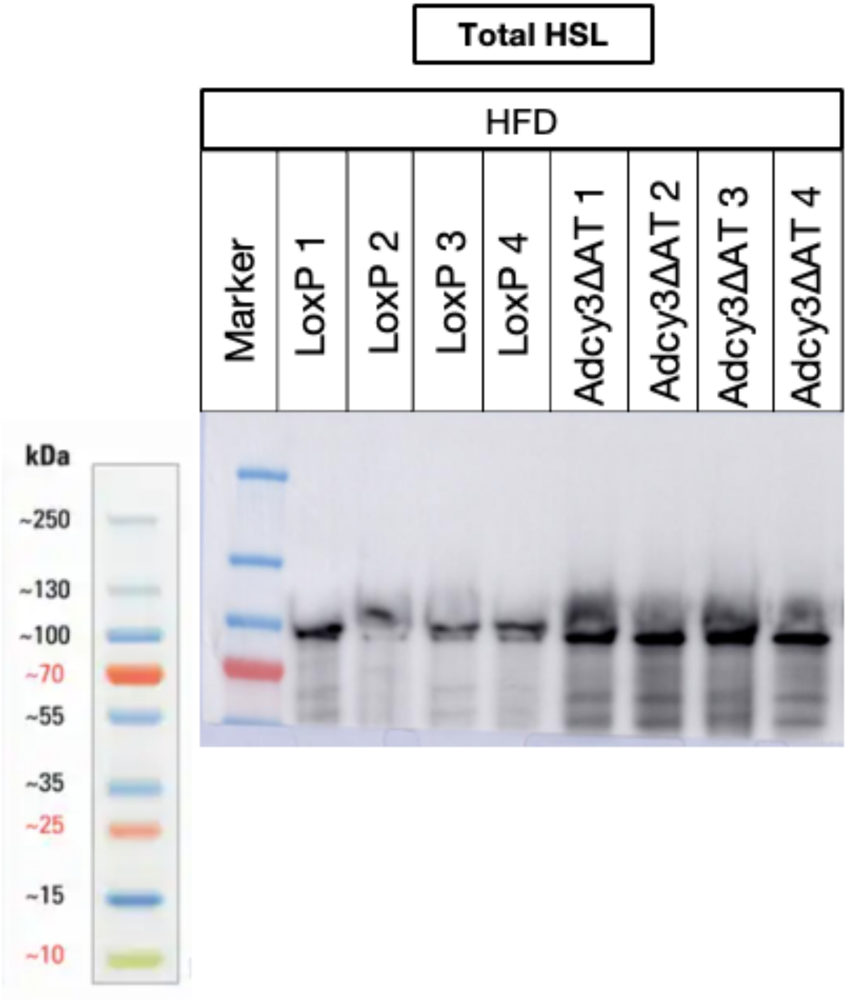
Total HSL
HFD
Marker
LoxP 1
LoxP 2
LoxP 3
LoxP 4
Adcy3ΔAT 1
Adcy3ΔAT 2
Adcy3ΔAT 3
Adcy3ΔAT 4
kDa
~250
~130
~100
~70
~55
~35
~25
~15
~10

Figure 5A

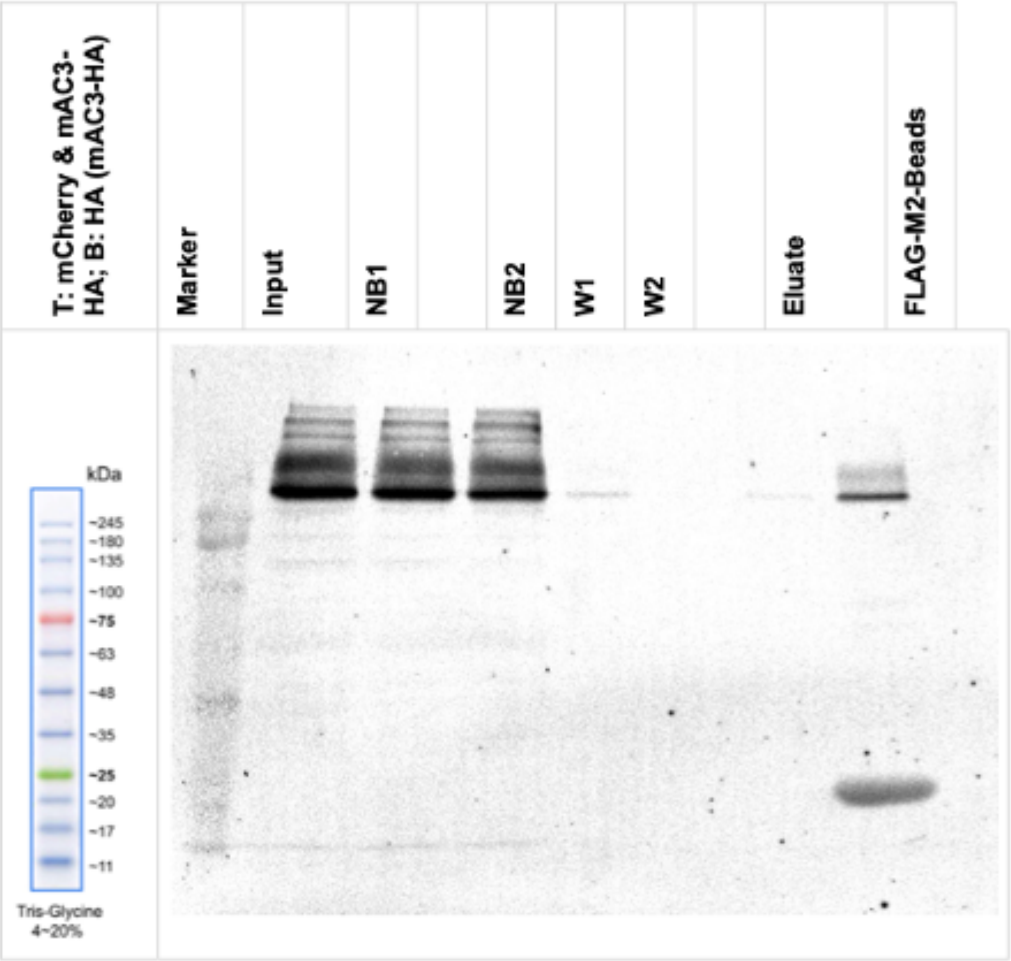

T: mCherry & mAC3-HA; B: HA (mAC3-HA)
Marker
Input
NB1
NB2
W1
W2
Eluate
FLAG-M2-Beads
kDa
~245
~180
~135
~100
~75
~63
~48
~35
~25
~20
~17
~11
Tris-Glycine 4~20%

Figure 5C

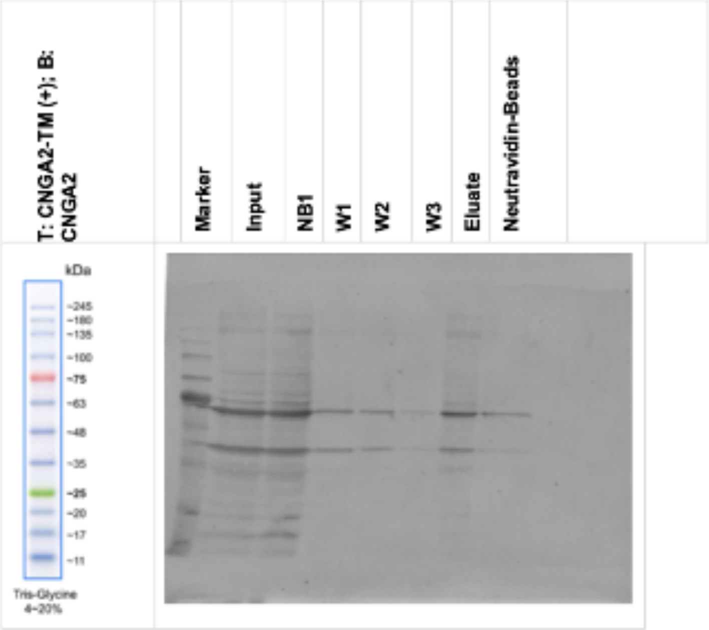
T: CNGA2-TM (+); B: CNGA2
Marker
Input
NB1
W1
W2
W3
Eluate
Neutravidin-Beads
kDa
Tris-Glycine 4~20%

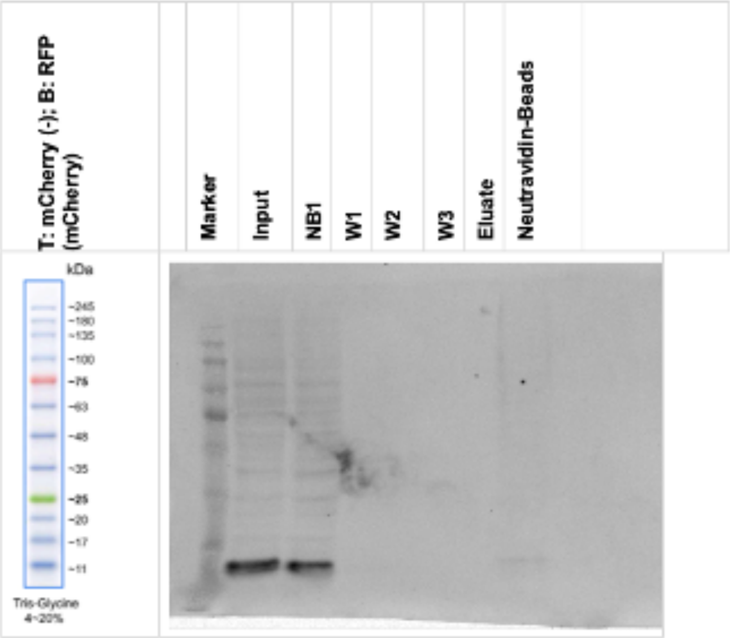
T: mCherry (-); B: RFP (mCherry)
Marker
Input
NB1
W1
W2
W3
Eluate
Neutravidin-Beads
kDa
Tris-Glycine 4~20%

Figure 5C_2

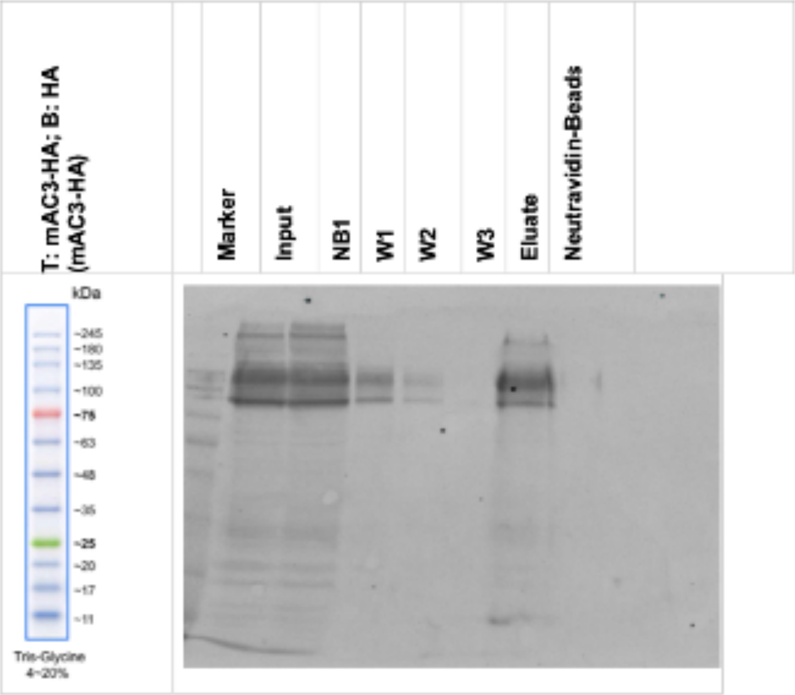
T: mAC3-HA; B: HA
(mAC3-HA)
Marker
Input
NB1
W1
W2
W3
Eluate
Neutravidin-Beads
kDa
~245
~180
~135
~100
~75
~63
~48
~35
~25
~20
~17
~11
Tris-Glycine
4~20%

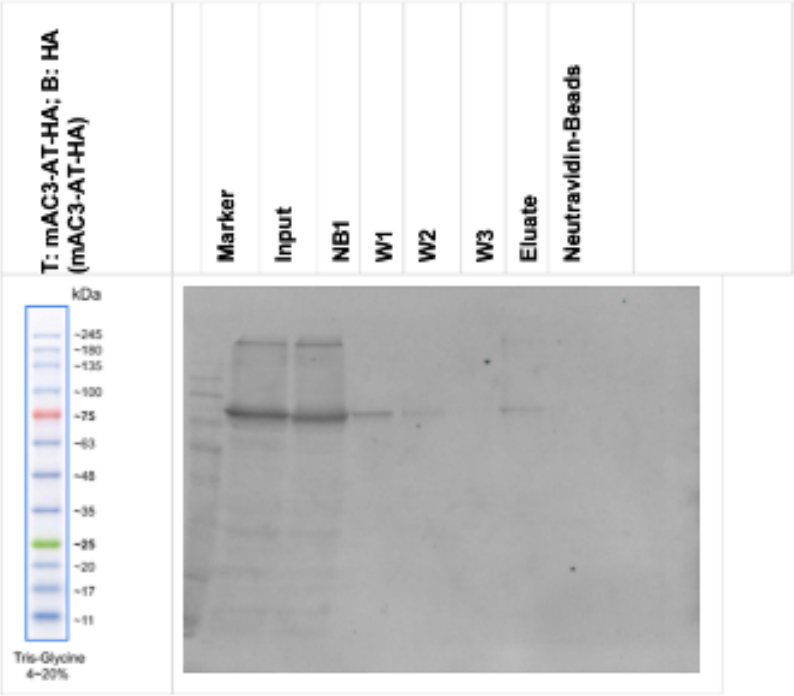
T: mAC3-AT-HA; B: HA
(mAC3-AT-HA)
Marker
Input
NB1
W1
W2
W3
Eluate
Neutravidin-Beads
kDa
~245
~180
~135
~100
~75
~63
~48
~35
~25
~20
~17
~11
Tris-Glycine
4~20%

Figure 5D-E

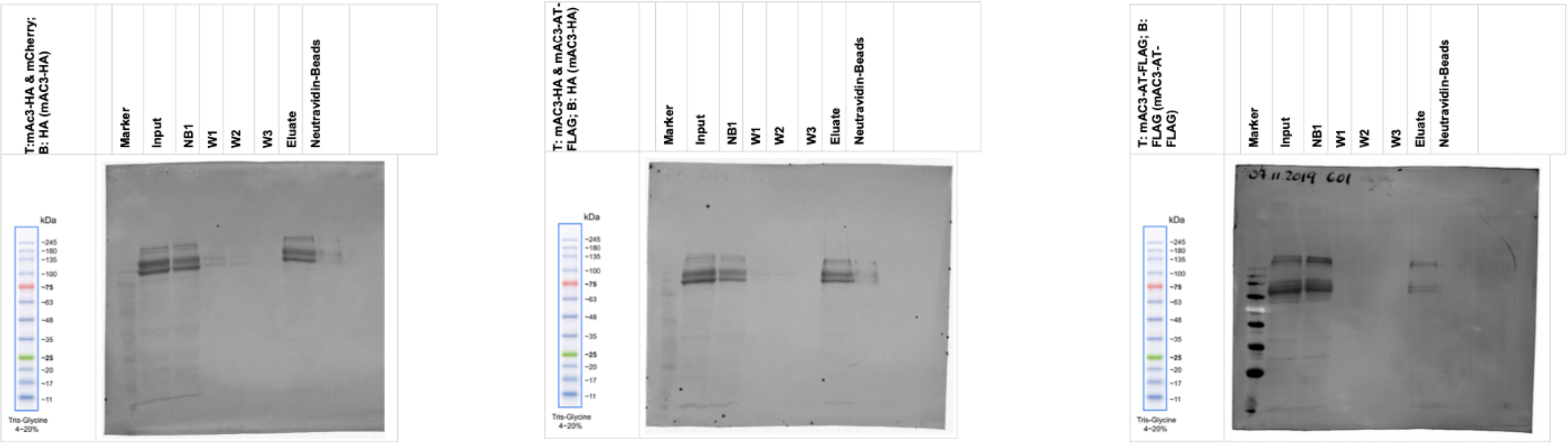
T:mAc3-HA & mCherry; B: HA (mAC3-HA)
Marker
Input
NB1
W1
W2
W3
Eluate
Neutravidin-Beads
kDa
~245
~180
~135
~100
~75
~63
~48
~35
~25
~20
~17
~11
Tris-Glycine 4~20%
T: mAC3-HA & mAC3-AT-FLAG; B: HA (mAC3-HA)
Marker
Input
NB1
W1
W2
W3
Eluate
Neutravidin-Beads
kDa
~245
~180
~135
~100
~75
~63
~48
~35
~25
~20
~17
~11
Tris-Glycine 4~20%
T: mAC3-AT-FLAG; B: FLAG (mAC3-AT-FLAG)
Marker
Input
NB1
W1
W2
W3
Eluate
Neutravidin-Beads
kDa
~245
~180
~135
~100
~75
~63
~48
~35
~25
~20
~17
~11
Tris-Glycine 4~20%

Figure 5F

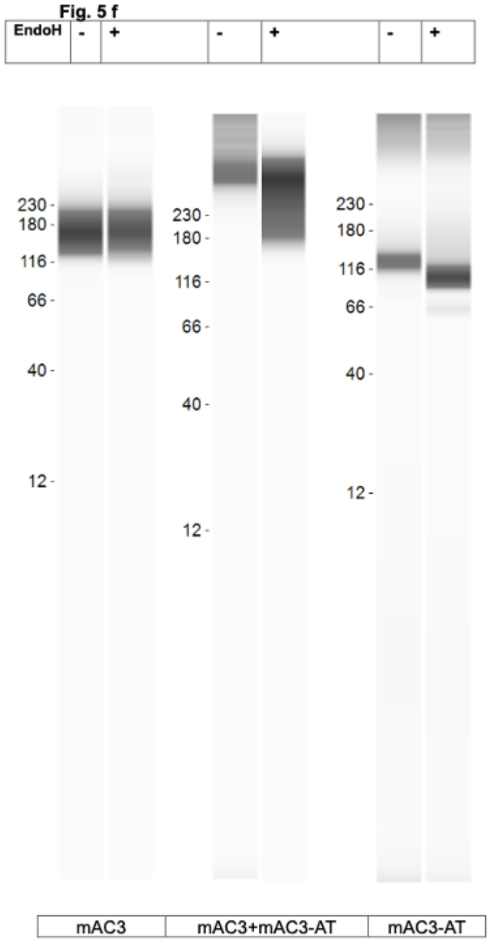

Fig. 5 f
EndoH
-
+
-
+
-
+
230
180
116
66
40
12
230
180
116
66
40
12
230
180
116
66
40
12
mAC3
mAC3+mAC3-AT
mAC3-AT

Figure 5G-H

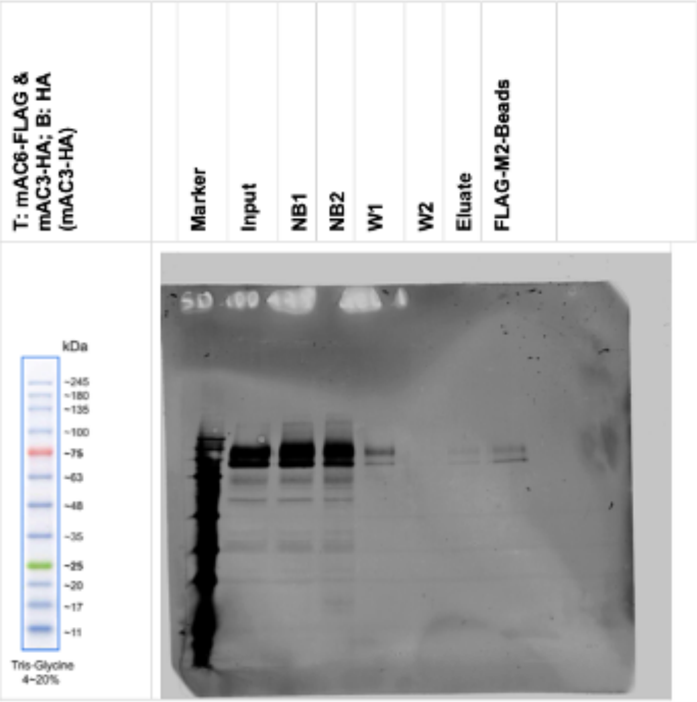
T: mAC6-FLAG & mAC3-HA; B: HA (mAC3-HA)
Marker
Input
NB1
NB2
W1
W2
Eluate
FLAG-M2-Beads
kDa
~245
~180
~135
~100
~75
~63
~48
~35
~25
~20
~17
~11
Tris-Glycine 4~20%

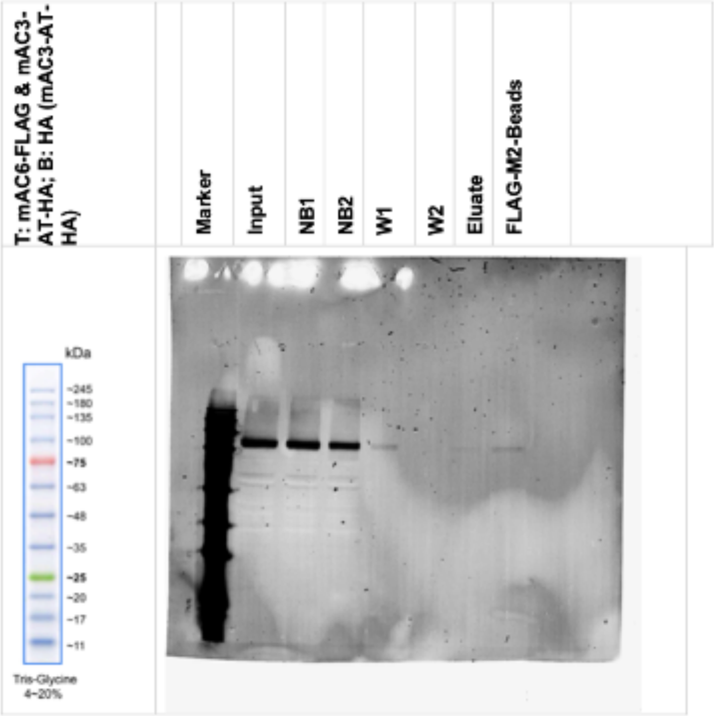
T: mAC6-FLAG & mAC3-AT-HA; B: HA (mAC3-AT-HA)
Marker
Input
NB1
NB2
W1
W2
Eluate
FLAG-M2-Beads
kDa
~245
~180
~135
~100
~75
~63
~48
~35
~25
~20
~17
~11
Tris-Glycine 4~20%

Figure 6C-D

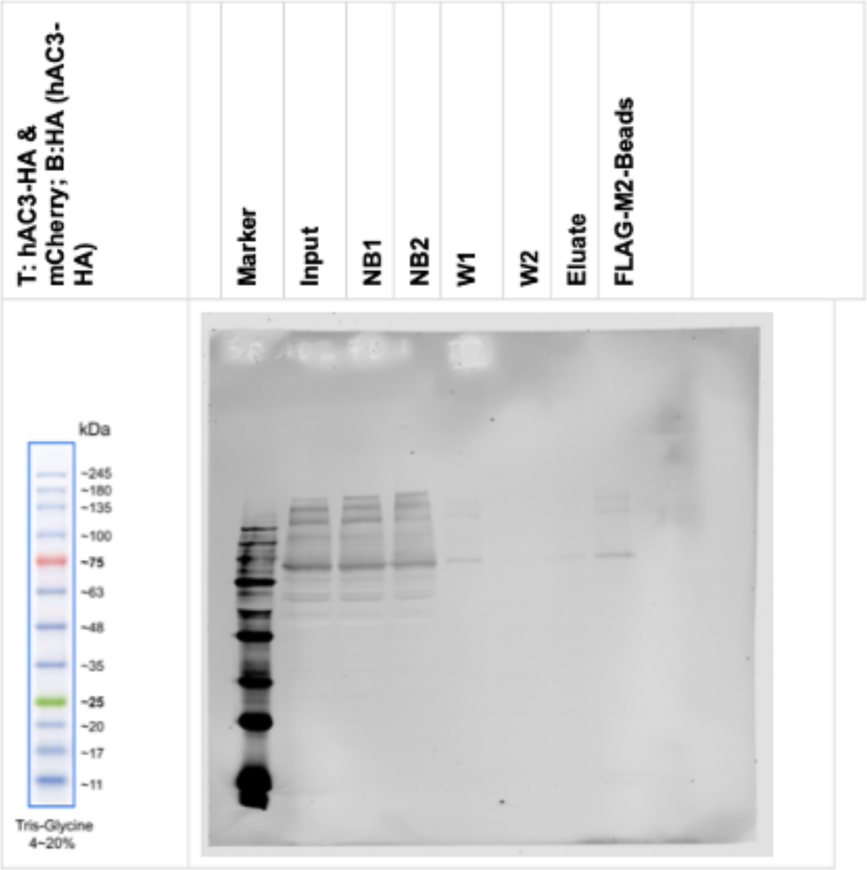
T: hAC3-HA & mCherry; B:HA (hAC3-HA)
Marker
Input
NB1
NB2
W1
W2
Eluate
FLAG-M2-Beads
kDa
~245
~180
~135
~100
~75
~63
~48
~35
~25
~20
~17
~11
Tris-Glycine 4~20%

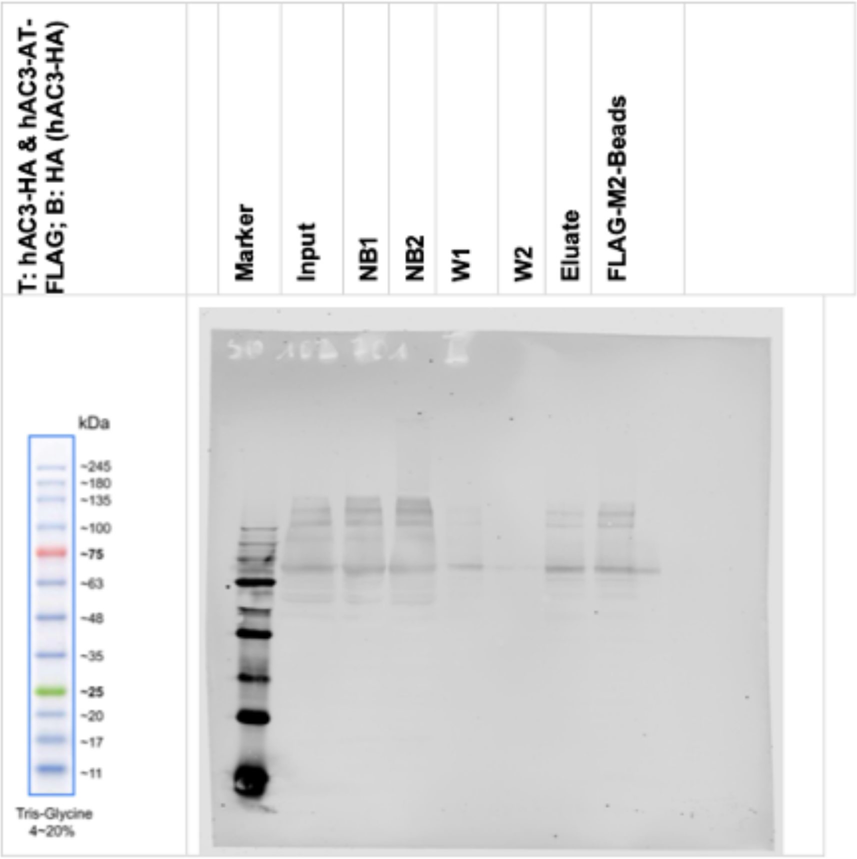
T: hAC3-HA & hAC3-AT-FLAG; B: HA (hAC3-HA)
Marker
Input
NB1
NB2
W1
W2
Eluate
FLAG-M2-Beads
kDa
~245
~180
~135
~100
~75
~63
~48
~35
~25
~20
~17
~11
Tris-Glycine 4~20%

Figure 6E-F

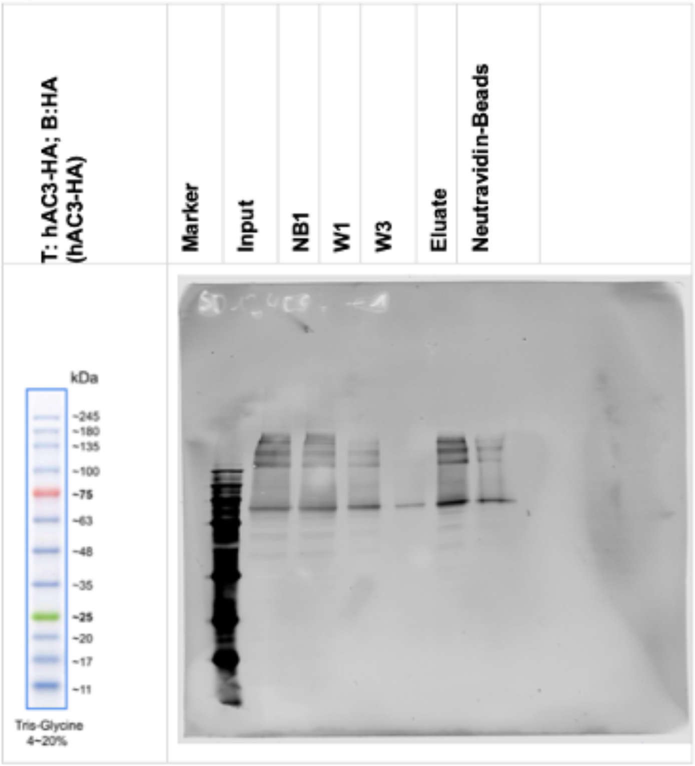

T: hAC3-HA; B:HA (hAC3-HA)
Marker
Input
NB1
W1
W3
Eluate
Neutravidin-Beads
kDa
~245
~180
~135
~100
~75
~63
~48
~35
~25
~20
~17
~11
Tris-Glycine 4~20%

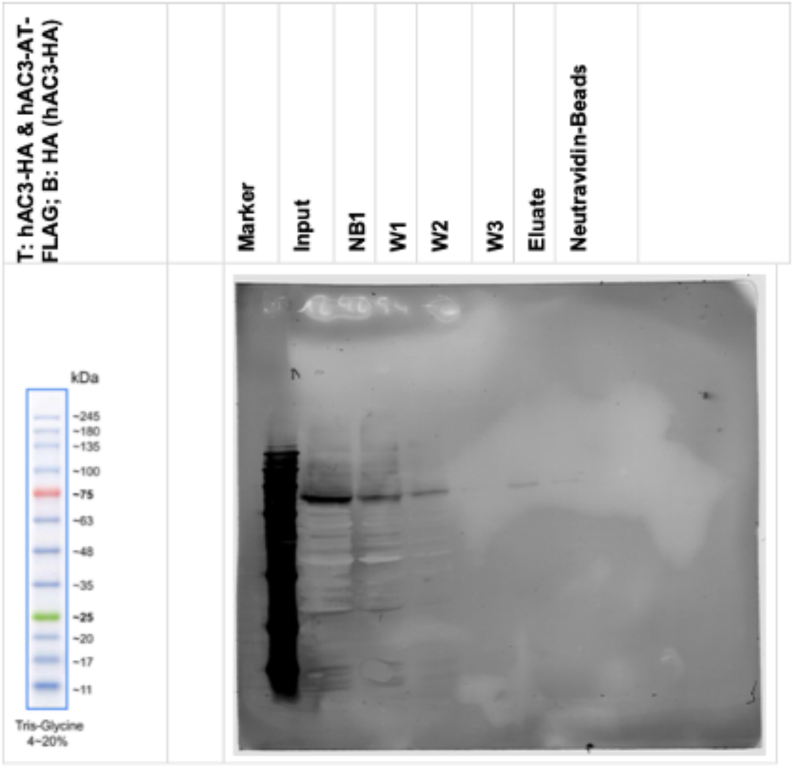

T: hAC3-HA & hAC3-AT-FLAG; B: HA (hAC3-HA)
Marker
Input
NB1
W1
W2
W3
Eluate
Neutravidin-Beads
kDa
~245
~180
~135
~100
~75
~63
~48
~35
~25
~20
~17
~11
Tris-Glycine 4~20%

Figure 6G

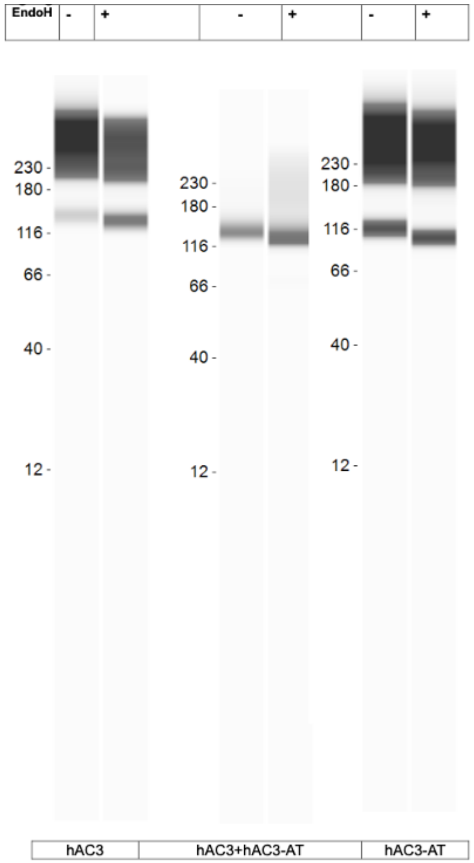

EndoH
-
+
-
+
-
+
230
180
116
66
40
12
230
180
116
66
40
12
230
180
116
66
40
12
hAC3
hAC3+hAC3-AT
hAC3-AT
