# Supplementary figures and images for: Cold-induced expression of a truncated adenylyl cyclase 3 acts as rheostat to brown fat function
Source: Nat Metab. 2024 Apr 29;6(6):1053–75. doi: 10.1038/s42255-024-01033-8 (PMC11971047; doi:10.1038/s42255-024-01033-8)

Figure S3e

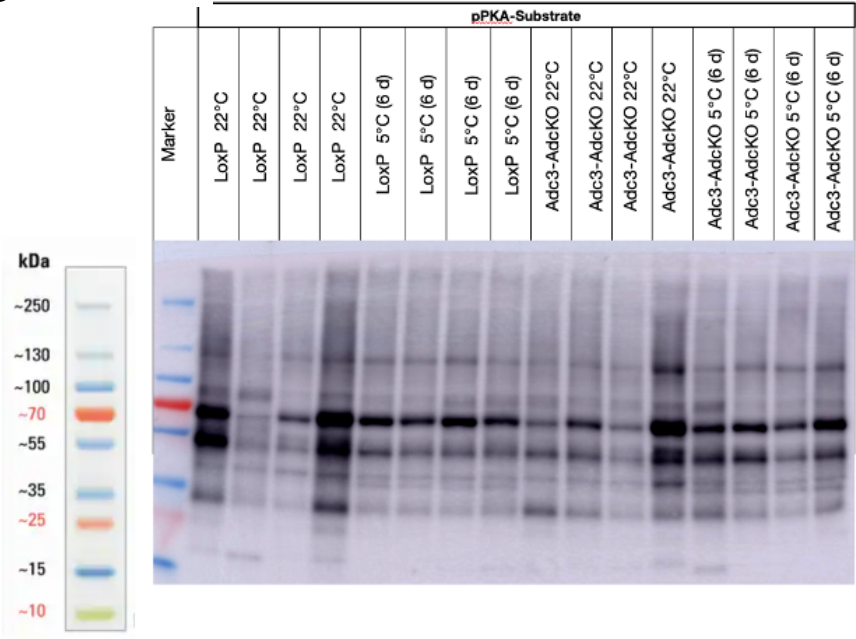

Figure S3e

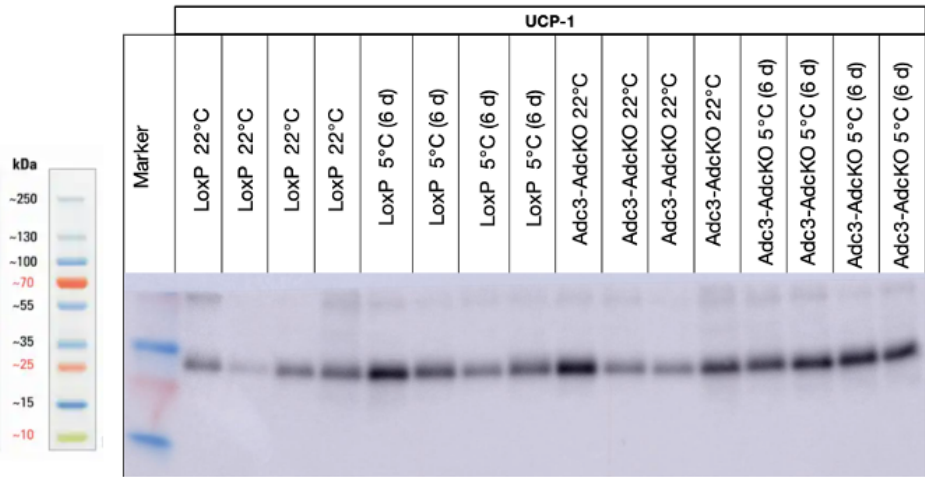

Figure S3e

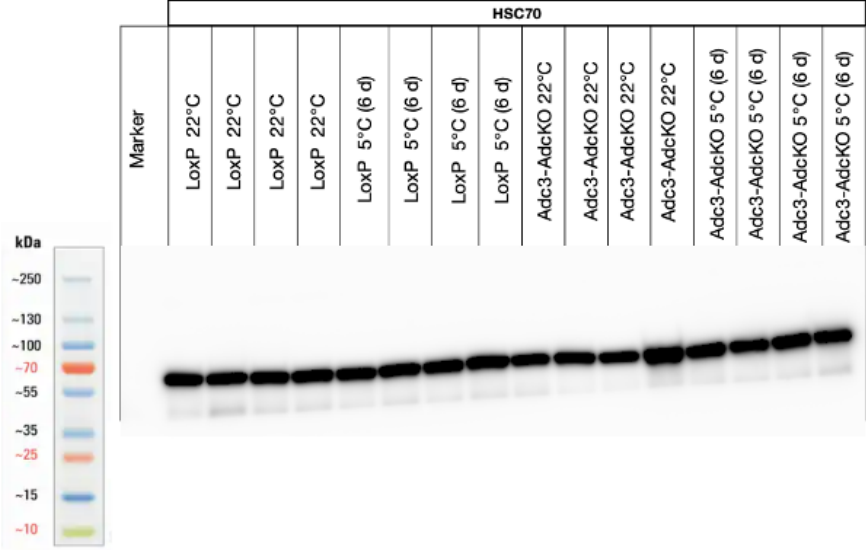

Figure S6c

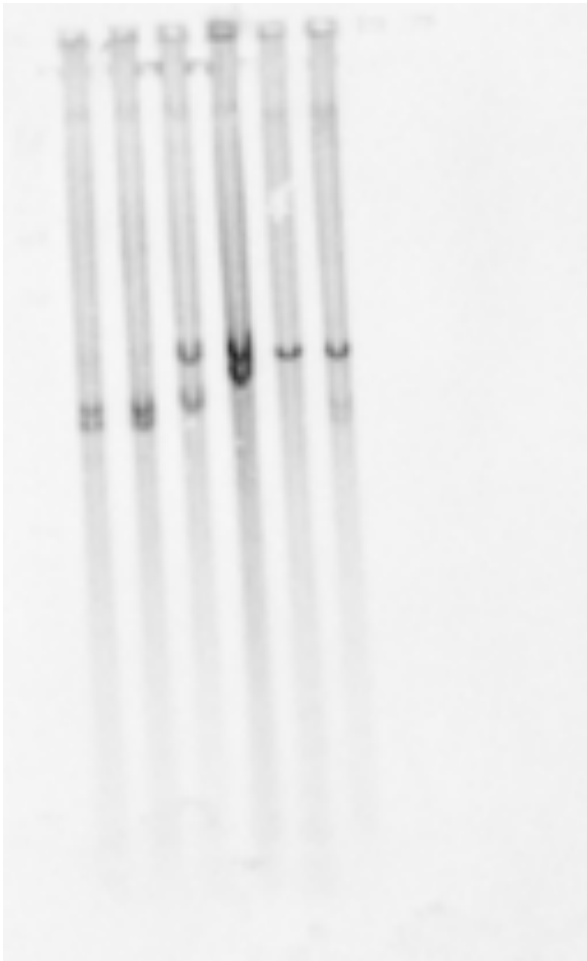

Figure S6d

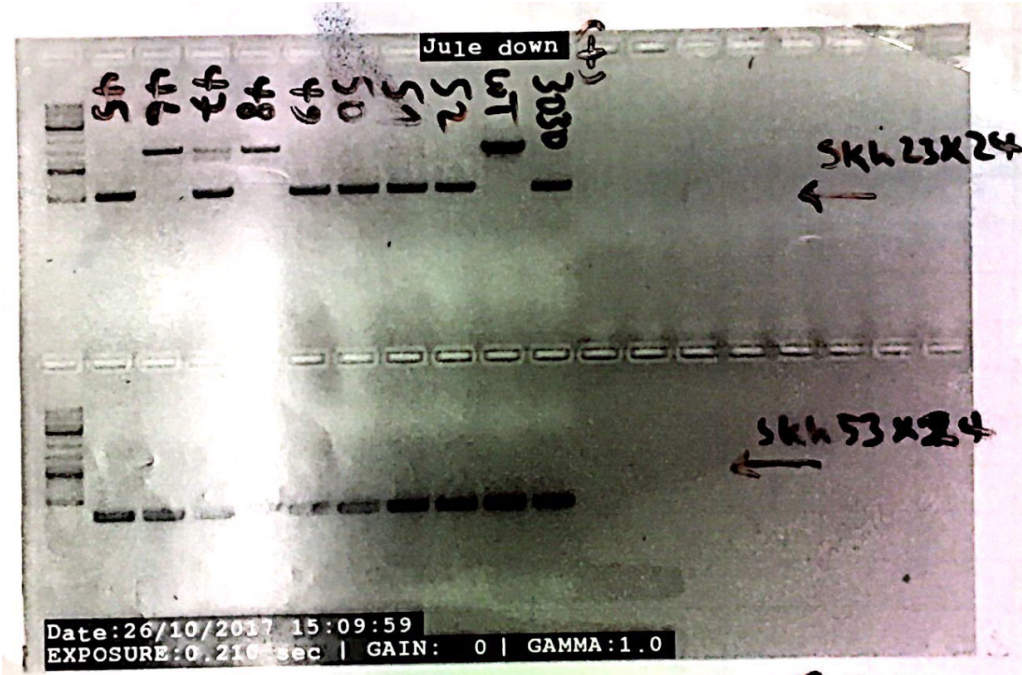

Supplement: Supplementary file 11 — Uncropped blots for Extended Data Figs. 1–9 [file 42255_2024_1033_MOESM11_ESM.pdf]
